# NRF2 coordinates cystine import and glutathione use to sustain ferroptosis resistance and formaldehyde tolerance in cancer

**DOI:** 10.1126/sciadv.aea1431

**Published:** 2026-10-23

**Authors:** Agustín E. Morellato, Natividad Alquezar-Artieda, Alejandro Guerrero-Maldonado, Rajinder Singh, Lilla Beja, Carla Umansky, Laura Perich-Pallaruelo, Mireia Pellicer, Paula Fernandez-Deu, Nicolas Aira, Nour Bourached-Silva, Uciel Chorostecki, Donald J. L. Jones, Gaël Roué, Richard J. Hopkinson, Lucas B. Pontel

**Affiliations:** ^1^ Josep Carreras Leukaemia Research Institute (IJC), 08916 Badalona, Spain.; ^2^ Instituto de Investigación en Biomedicina de Buenos Aires (IBioBA), CONICET-Partner Institute of the Max Planck Society, Buenos Aires, Argentina.; ^3^ Universitat de Barcelona, 08028 Barcelona, Spain.; ^4^ Leicester Cancer Research Centre, University of Leicester, Leicester Royal Infirmary, Infirmary Square, Leicester LE1 7LX, UK.; ^5^ Institute for Structural and Chemical Biology and School of Chemistry, University of Leicester, Lancaster Road, Leicester LE1 7RH, UK.; ^6^ Department of Biomedical Sciences, Universitat Internacional de Catalunya, Sant Cugat del Vallès, Catalunya 08195, Spain.; ^7^ Leicester van Geest Multi-Omics Facility, University of Leicester, Hodgkin Building, Lancaster Road, Leicester LE1 7RH, UK.

## Abstract

Cancer cells must balance detoxification of reactive metabolic by-products while preserving redox homeostasis, but how this trade-off is managed remains unclear. The Kelch-like ECH-associated protein 1 (KEAP1)–nuclear factor–erythroid 2–related factor 2 (NRF2) pathway, a key regulator of antioxidant defenses, is frequently hyperactivated in cancer. Formaldehyde, a genotoxic one-carbon by-product, is typically detoxified via glutathione (GSH), which is also essential for suppressing ferroptotic cell death. Here, we show that NRF2 promotes formaldehyde tolerance through up-regulation of the cystine transporter SLC7A11, enabling a protective mechanism that is largely independent of GSH, which remains necessary for ferroptosis resistance. In NRF2-active cells, formaldehyde reacts with cysteine to form a thiazolidine conjugate, and the combination of formaldehyde stress and SLC7A11 inhibition activates the integrated stress response, suppresses global protein synthesis, and markedly compromises cell viability. Together, these findings reveal that NRF2 coordinates ferroptosis resistance and formaldehyde tolerance, exposing a cancer vulnerability through combined inhibition of cystine uptake and GSH synthesis.

## INTRODUCTION

Tumor cells must adapt to the stresses imposed by their own altered metabolism. One of these challenges is the accumulation of reactive by-products, including formaldehyde (FA)—a small, highly reactive aldehyde generated from central metabolic reactions such as the one-carbon cycle, and histone and RNA demethylation reactions (*1*–*4*). If not cleared efficiently, FA can damage nucleic acids and proteins causing genome instability (*5*), redox disruption (*6*), and epigenetic dysregulation (*7*), ultimately leading to cellular toxicity and likely underlying the onset of human bone marrow failure conditions like Fanconi anemia and aldehyde degradation deficiency syndrome (ADDS, a.k.a. AMeDS) (*8*–*10*). Most cells detoxify FA through conjugation with glutathione (GSH) (*6*, *11*) (Fig. 1A), a key antioxidant that also defends against reactive oxygen species (ROS) (*12*). This dual function creates a metabolic challenge in cancer cells, which rely heavily on GSH to manage oxidative stress but must also continuously neutralize endogenously produced FA. Resolving this conflict likely involves specialized metabolic adaptations that are not yet fully defined.

**Fig. 1.**
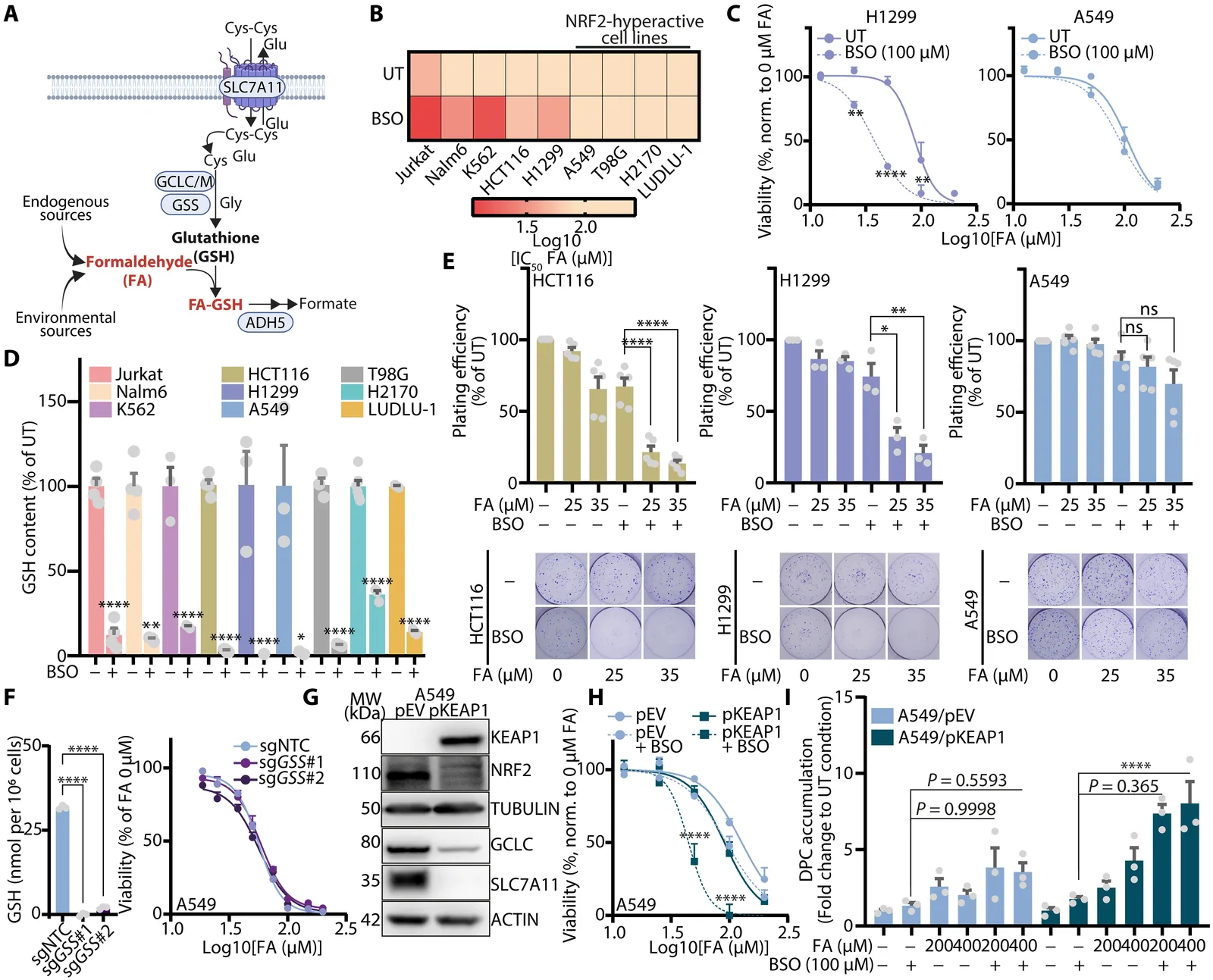
NRF2 controls the use of glutathione in formaldehyde catabolism. (**A**) Overview of cytosolic formaldehyde (FA) catabolism. (**B**) Heatmap depicting Log10 of the 50% FA inhibitory concentration (IC_50_) in the presence or absence of l-buthionine-sulfoximine (BSO) at concentrations of 25 μM (H2170 cells), 50 μM (Jurkat, Nalm6, K562, and LUDLU-1 cells) or 100 μM (HCT116, H1299, A549, and T98G cells). (**C**) Representative FA viability curves normalized (norm.) to 0 μM FA for H1299 and A549 cells [mean ± SEM, *n* = 5 (H1299), *n* = 6 (A549), and two-way ANOVA (Tukey test between untreated (UT) and 100 μM BSO); for H1299: **P = 0.0016, ****P < 0.0001, and **P = 0.0023]. (**D**) Glutathione (GSH) levels upon 24-hour exposure to BSO [same concentrations as (B)] (mean ± SEM, *n* represented as dots, *t* test comparison between UT and BSO; *P = 0.0142, **P = 0.0073, ****P < 0.0001). (**E**) Colony survival assay (CSA) in HCT116, H1299, and A549 in the presence of FA and/or 3.12 μM BSO for H1299 cells and 6.25 μM BSO for both HCT116 and A549 cells [mean ± SEM, *n* represented as dots, one-way ANOVA (Tukey test); for HCT116: ****P < 0.0001; for H1299: *P = 0.0106 and **P = 0.0033]. (**F**) Left: GSH quantification in A549 cells expressing a nontargeting-control sgRNA (sgNTC) or sgRNAs against *GSS* (sg*GSS*). Right: Viability in response to FA [mean ± SEM, *n* = 3]. (**G**) Immunoblots in A549 cells carrying an empty vector (pEV) or a vector coding for wild-type *KEAP1* (p*KEAP1*). (**H**) Viability curves for A549/pEV or A549/pKEAP1 cells exposed to different concentrations of FA in the presence of 100 μM BSO [mean ± SEM, *n* = 3 and two-way ANOVA (Tukey test between UT and 100 μM BSO); ****P < 0.0001]. (**I**) DNA-protein cross-link (DPC) accumulation in A549/pEV and A549/pKEAP1 cells exposed to 100 μM BSO for 48 hours and to the indicated concentrations of FA for 4 hours (mean ± SEM, *n* = 3, one-way ANOVA in each cell line corrected by Tukey test) (*5*). Created in BioRender. https://BioRender.com/tsuwhsf.

One major system that helps cells manage oxidative stress is the Kelch-like ECH-associated protein 1 (KEAP1)–nuclear factor–erythroid 2–related factor 2 (NRF2, coded by *NFE2L2*) pathway (*13*). Under normal conditions, KEAP1 binds NRF2 and targets it for rapid proteasomal degradation, keeping NRF2 activity low. However, in response to oxidative stress—or through somatic mutations commonly found in cancer—NRF2 becomes stabilized and activates a transcriptional program that promotes antioxidant defense (*14*). This includes up-regulation of genes involved in GSH synthesis (GCLC/GCLM) and import of cystine (xCT/SLC7A11), a relevant precursor of cysteine in cancer cells (*15*–*17*). NRF2 also cooperates with the integrated stress response (ISR) to fine-tune cellular adaptation to stress challenges (*18*, *19*). Hyperactivation of NRF2 is particularly common in non–small cell lung cancer (NSCLC) (*20*) and has also been observed in head and neck squamous cell carcinoma (HNSC), among other tumors. In these cancers, NRF2 contributes to chemotherapy resistance and suppresses ferroptosis, a regulated form of cell death driven by lipid peroxidation (*21*, *22*).

Although NRF2 is broadly recognized as a regulator of antioxidant programs, its role in coordinating GSH usage across competing metabolic demands remains less well understood (*23*). Intracellular GSH concentrations can reach 1 to 10 mM (*24*), yet its consumption is continuous and multifaceted—supporting both detoxification of reactive aldehydes like FA or methylglyoxal (MGO), while serving as a cosubstrate for enzymes that counteract lipid peroxidation and ferroptosis like GSH peroxidase 4 (GPX4) (*25*–*27*). GSH synthesis depends on the availability of cysteine, which is typically found at ∼100 to 200 μM in normal cells but can rise to millimolar levels in NRF2-hyperactive cancer cells due to increased cystine uptake (*28*). As a result, the balance between GSH and cysteine use for detoxification, GSH synthesis, and antioxidant defense may become a critical point of regulation, affecting not only redox homeostasis but also other cysteine-dependent functions like coenzyme A production (*29*) and mitochondrial Fe-S cluster assembly (*30*).

Here, we identify a metabolic rewiring mediated by hyperactive KEAP1-NRF2 signaling, in which elevated expression of the cystine transporter SLC7A11 redirects FA catabolism away from GSH, which remains necessary for ferroptosis resistance. These findings define NRF2 as a central hub in cancer that determines the use of GSH in either FA catabolism or ferroptosis resistance, suggesting a potential therapeutic strategy targeting cystine uptake and GSH synthesis.

## RESULTS

### Cancer cells have GSH-dependent and GSH-independent FA protections

Motivated by results showing that FA causes oxidative stress and reacts with GSH in human cancer cells and *Caenorhabditis elegans* (*6*), we sought to address whether cancer cells predominantly depend on GSH for protection against FA. First, we determined FA tolerance in multiple cancer cells from different origins, assessing also whether they require GSH for FA detoxification (Fig. 1, B and C, and fig. S1A). Several cancer cell lines from hematological (Jurkat: T-acute lymphoblastic leukemia, Nalm6: B-ALL, K562: chronic myeloid leukemia) and solid tumors (HCT116: colorectal cancer; H1299: NSCLC) were sensitized to FA in conditions of GSH synthesis inhibition, achieved by noncytotoxic concentrations of the GSH synthesis inhibitor l-buthionine-sulfoximine (BSO) (Fig. 1D and fig. S1B). In contrast, A549 [lung adenocarcinoma (LUAD)]; LUDLU-1 and H2170 (lung squamous cell carcinoma); and T98G (glioblastoma) cell lines were not further sensitized to FA upon depletion of GSH. BSO caused more than 70% depletion of GSH (Fig. 1D and fig. S1C), indicating that the lack of dependency on GSH for FA protection was not a consequence of incomplete GSH depletion. Consistent results were obtained by determining plating efficiency (Fig. 1E), even at lower BSO concentrations, as 100 μM severely impaired colony formation independently of FA. To rule out that the residual GSH under BSO exposure accounted for the lack of protection against FA, we genetically inactivated GSH synthetase (GSS), which catalyzes the addition of glycine to the dipeptide γ-glutamyl-cysteine (fig. S1D). A549 cells tolerate loss of GSS because γ-glutamyl-cysteine can partially substitute for several functions of GSH (*17*). Notably, *GSS*-deficient cells lacked detectable GSH and yet did not exhibit increased sensitivity to FA (Fig. 1F), despite showing sensitivity to *tert*-butyl-hydroperoxide (TBHP) (fig. S1E), further indicating that certain cancer cell lines do not mainly rely on GSH for protection against FA.

Among the cell lines showing less dependency on GSH for FA detoxification, we identified LUDLU-1, H2170, T98G, and A549 (Fig. 1B). These cell lines exhibit hyperactivation of the NRF2 transcriptional program, which, in A549 and H2170, results from inactivating mutations in *KEAP1* (*31*, *32*). To directly test whether *KEAP1* loss, and thus NRF2 activation, limits the use of GSH for FA detoxification, we reintroduced wild-type *KEAP1* in A549 and H2170 cells. We first confirmed efficient GSH depletion under the BSO concentrations used in these models (fig. S1F) and validated the down-regulation of NRF2 signaling by measuring the expression of NRF2, GCLC, and SLC7A11, all coded by canonical NRF2-target genes (*18*) (Fig. 1G and fig. S1G). *KEAP1* complementation restored the requirement of GSH in FA protection in both A549 and H2170 cells (Fig. 1H and fig. S1, H and I), indicating they can tolerate FA without primarily relying on GSH.

A major consequence of FA overload is the formation of cytotoxic DNA-protein cross-links (DPCs) (*5*, *33*). Therefore, the persistence of FA tolerance in NRF2-hyperactive cells under conditions of GSH depletion could, in principle, reflect activation of downstream survival mechanisms. We thus quantified DPC accumulation following FA exposure in A549/pKEAP1 cells. *KEAP1*-complemented A549 cells exhibited a significantly greater fold increase in DPC upon FA challenge and GSH depletion compared with noncomplemented A549/pEV cells (Fig. 1I). These findings suggest that the FA-protective mechanism(s) operating in NRF2-hyperactive cells under GSH-limiting conditions acts upstream of FA-inflicted genome damage, consistent with a reduction in intracellular FA availability.

### *KEAP1-NFE2L2* activating mutations up-regulate GSH synthesis and SLC7A11

*KEAP1-NFE2L2* activating mutations are prevalent in several tumors, including LUAD, HNSC, and esophageal carcinoma (ESCA) (fig. S2A). In lung tumors and HNSC, oncogenic mutations in *KEAP1* or *NFE2L2* (coding for NRF2) are associated with poor clinical outcomes (fig. S2B). To identify factors that might explain the reduced reliance on GSH for FA detoxification, we analyzed the mRNA expression from the TCGA-LUAD cohort, stratifying samples by the presence of *KEAP1* or *NFE2L2* driver mutations*. KEAP1-NFE2L2*-mutated tumors showed up-regulation of the GSH synthesis genes *GCLM* and *GCLC*, as well as the cystine/glutamate antiporter *SLC7A11*, among other NRF2 target genes (Fig. 2, A and B). *ADH5*, which codes for the GSH-dependent FA-catabolic enzyme alcohol dehydrogenase 5, was highly expressed in both *KEAP1-NFE2L2–*mutated and wild-type tumors, presenting a slight increase in mutated biopsies (Fig. 2C).

**Fig. 2.**
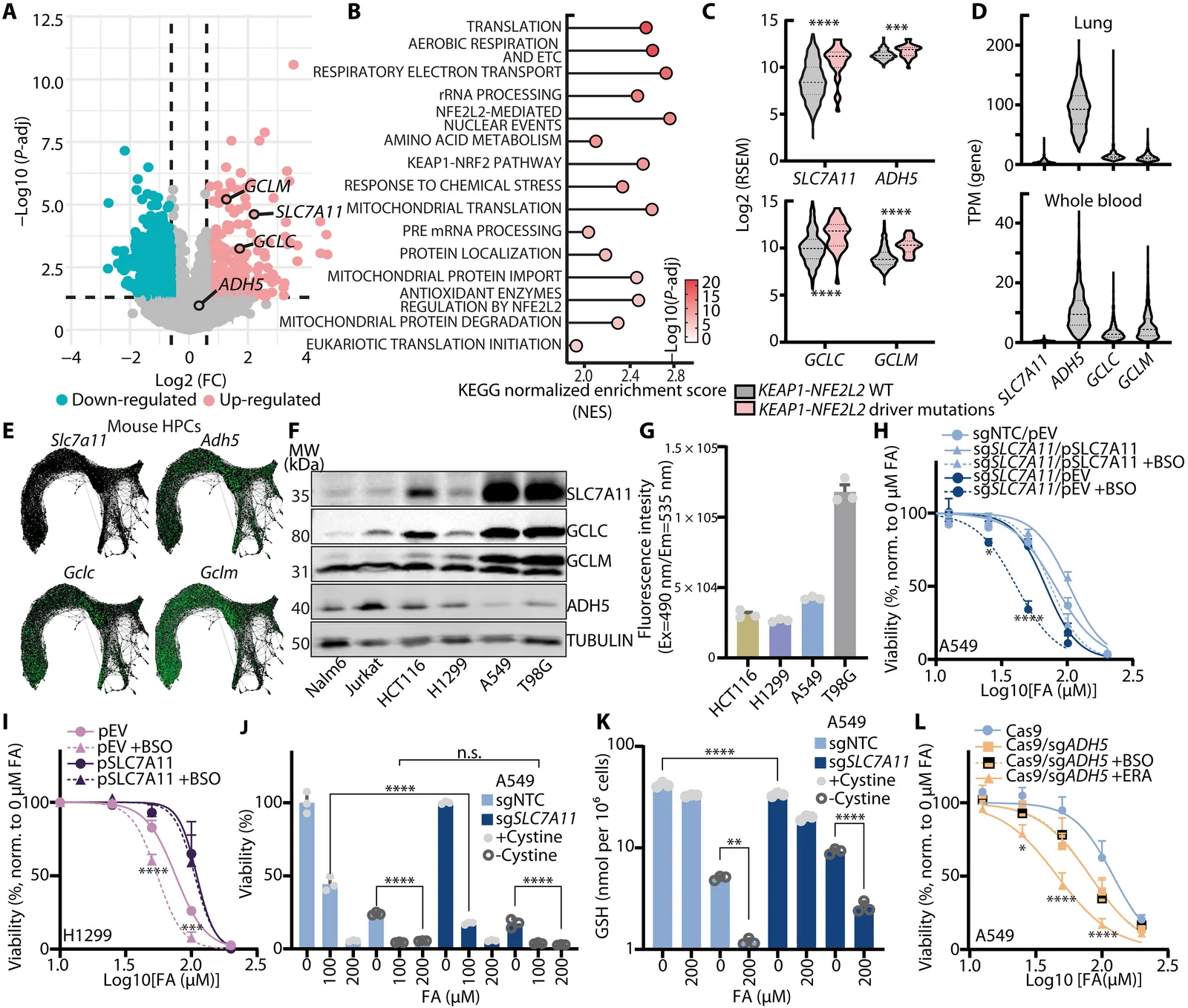
SLC7A11 expression modulates formaldehyde tolerance. (**A**) Volcano plot showing dysregulated genes in lung adenocarcinoma tumors (TCGA-LUAD cohort) carrying driver mutations in *NFE2L2* or *KEAP1* [cutoffs: Log2(FC) = ±0.6 and *P*-adj ≤ 0.05]. (**B**) Gene Set Enrichment Analysis (GSEA) using Kyoto Encyclopedia of Genes and Genomes (KEGG) gene sets in the TCGA-LUAD cohort with mutations in *NFE2L2* or *KEAP1*. (**C**) mRNA expression of selected genes involved in formaldehyde (FA) detoxification in *KEAP1-NFE2L2* mutated and nonmutated (WT) LUAD tumors. (**D**) mRNA expression of selected genes in normal lung and whole blood obtained from GTEX. (**E**) Single-cell RNA-seq data from mouse hematopoietic and progenitor cells (HPC) plotted with kleintools (https://kleintools.hms.harvard.edu/tools/spring.html) (*58*). (**F**) Representative images of Western blots of selected factors from (A) performed in different cell lines. (**G**) Cystine uptake assay performed in selected cell lines (mean ± SEM, *n* = 3). (**H**) FA tolerance of A549 cells lacking *SLC7A11* (sg*SLC7A11*) complemented with a wild-type copy of *SLC7A11* (pSLC7A11) grown in the presence of 100 μM BSO (mean ± SEM, *n* = 3 and two-way ANOVA, ****P < 0.0001). (**I**) FA tolerance for H1299 cells harboring empty vector (pEV) or pSLC7A11 in the presence of 100 μM BSO [mean ± SEM, *n* = 3 and two-way ANOVA (Tukey test between untreated (UT) and BSO at each point); for H1299 pEV: ****P < 0.0001 and ***P = 0.0002]. (**J**) Viability of A549 cells carrying sg*SLC7A11* and sgNTC (nontargeting control) under cystine deprivation [one-way ANOVA (Tukey test)]. (**K**) Glutathione (GSH) levels of the indicated cell lines maintained during 48 hours under cystine deprivation and exposing to FA during the last 24 hours [one-way ANOVA (Tukey test), **P = 0.0058]. (**L**) FA tolerance of A549 cells lacking *ADH5* in the presence of BSO (100 μM) or ERA (5 μM) [mean ± SEM, *n* = 6 and two-way ANOVA (Tukey test between UT and BSO or ERA at each point for multiple comparison); *P = 0.0300 and ****P < 0.0001].

We next assessed the expression of these genes in normal tissues. In both human lung and whole blood, *SLC7A11* expression was minimal, while *ADH5*, *GCLC*, and *GCLM* were readily detectable (Fig. 2D). Similarly, single-cell RNA sequencing (scRNA-seq) data from mouse bone marrow hematopoietic progenitor cells—a major target of FA toxicity—confirmed negligible expression of *Slc7a11* in this tissue (Fig. 2E and fig. S2C) (*10*, *34*). In tumors, *SLC7A11* showed variable expression (fig. S2D). Notably, LUAD and acute myeloid leukemia (AML) tumors showing higher expression of *SLC7A11* resulted in poor overall patient survival (fig. S2E). We then extended this analysis to a panel of cancer cell lines and found that SLC7A11 and factors involved in GSH synthesis were consistently up-regulated in models with hyperactive KEAP1-NRF2 signaling (Fig. 2F and fig. S2F). Last, we observed that *SLC7A11* expression correlated with increased cystine uptake, suggesting that NRF2 hyperactivation drives enhanced import of cystine through SLC7A11 (Fig. 2G).

### SLC7A11 controls FA tolerance

To directly assess the role of SLC7A11 in FA tolerance, we first examined the effect of pharmacological inhibition. NRF2-hyperactive cells became more sensitive to FA upon treatment with the SLC7A11 inhibitors erastin or sulfasalazine at doses not causing more than 30% death (fig. S3, A and B). Then, we generated *SLC7A11*-knockout A549 cells and reconstituted them with a wild-type *SLC7A11* cDNA containing silent mutations in the sgRNA recognition site (fig. S3C). *SLC7A11*-deficient cells were highly sensitive to FA under conditions of GSH depletion induced by BSO, a phenotype that was fully reverted in the *SLC7A11*-complemented cells (Fig. 2H). Moreover, SLC7A11 conferred FA resistance beyond that observed in empty-vector control cells, indicating that SLC7A11 is not only required but can be rate limiting for FA tolerance. To further support that SLC7A11 expression is sufficient to shift FA protection away from GSH dependence, we overexpressed SLC7A11 in H1299 cancer cells (fig. S3D), which exhibit low basal expression of this transporter (Fig. 2F). In control cells (H1299/pEV), GSH depletion significantly increased FA toxicity. In contrast, *SLC7A11*-overexpressing cells (H1299/pSLC7A11) remained comparatively resistant to FA even under GSH-depleted conditions (Fig. 2I).

We reasoned that the increased cystine uptake observed in NRF2-hyperactive cells could enhance intracellular cysteine availability and therefore promote FA protection through cysteine conjugation. To test this hypothesis, we first addressed whether cysteine could mitigate FA toxicity by measuring the formation of three-dimensional (3D) spheres in the presence of FA and the membrane-permeable cysteine derivative *N*-acetylcysteine (NAC) in A549 cells. NAC fully rescued sphere formation in FA-treated cells (fig. S3E), suggesting a protective role for cysteine in counteracting FA toxicity. Because this experiment cannot determine whether FA reacts with thiol groups extracellularly or intracellularly, we next assessed FA tolerance in cells cultured in cystine-free medium. While *SLC7A11*-deficient cells were more sensitive to FA than wild-type controls under standard conditions, both cell lines showed a similar increase in FA sensitivity under cystine deprivation (Fig. 2J), a condition that severely impaired basal growth in both WT and *SLC7A11*-deficient cells. These results suggest that cystine availability is a key determinant of FA tolerance and are consistent with a role of SLC7A11 in cystine-dependent FA protection.

We next examined whether SLC7A11 perturbation affected GSH levels, given that cystine uptake is a relevant source of cysteine for GSH synthesis in cancer cells (*16*). Accordingly, *SLC7A11*-deficient cells contained lower GSH than sgNTC controls, and GSH levels were further reduced upon FA exposure (Fig. 2K). Notably, cystine deprivation caused a ∼10-fold decrease in GSH levels even in *SLC7A11*-deficient cells, indicating that extracellular cystine can partially sustain cysteine availability for GSH synthesis independently of SLC7A11. However, the FA-protective effect of SLC7A11 depends on cystine availability, overall supporting a role for SLC7A11-mediated cystine import in modulating FA tolerance.

The GSH-dependent FA protection initiates with the spontaneous reaction between FA and GSH, yielding *S*-hydroxymethyl-GSH (HMSGSH), which is the substrate of ADH5 (*6*). Because this enzyme retains a minor FA-catabolic activity in the absence of GSH (*11*), we interrogated whether it was still required for FA detoxification in NRF2-hyperactive cells. CRISPR-Cas9–mediated inactivation of *ADH5* in A549 cells increased FA sensitivity (Fig. 2L and fig. S3F), indicating that ADH5 contributes to FA protection in this model. *ADH5*-deficient cells were further sensitized to FA by erastin, but not BSO (Fig. 2L), showing that SLC7A11-mediated FA protection operates independently of both GSH and ADH5.

### FA conjugates with cysteine

FA can react with GSH and cysteine, yielding HMSGSH and the conjugate thiazolidine-4-carboxylic acid (thioproline), respectively (Fig. 3A) (*35*–*39*). To directly compare the reactivity of FA with these two nucleophiles in vitro, we combined cysteine (1 mM), GSH (5 mM), and FA (1 mM) and monitored the kinetics of HMSGSH and thioproline formation by nuclear magnetic resonance spectroscopy (NMR) (Fig. 3B). Under conditions enabling reliable NMR detection and that approximate the GSH:cysteine ratio reported in NRF2-hyperactive cells (*28*), FA reacted initially with GSH (within <143 s); however, cysteine efficiently outcompeted GSH over time, resulting in predominant formation of thioproline at a rate of 3.9 × 10^−3^ units/s (Fig. 3C). Fivefold lower concentrations of cysteine were sufficient to shift the balance from HMSGSH toward thioproline formation (fig. S4A). These data indicate that although FA can react with both GSH and cysteine, the cysteine-FA conjugate prevails in vitro.

**Fig. 3.**
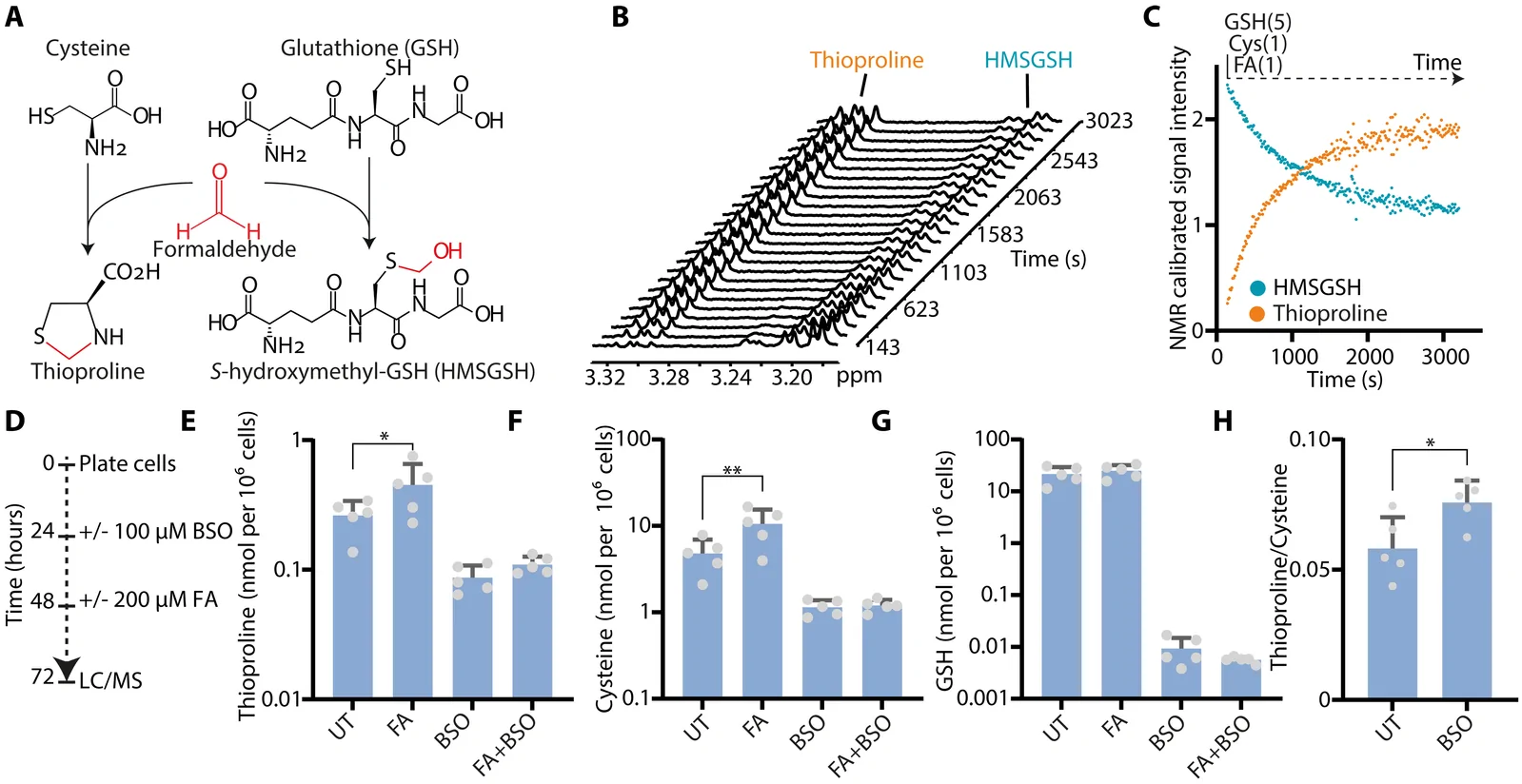
Formaldehyde reacts with cysteine. (**A**) Reaction between formaldehyde (FA) and glutathione (GSH) or cysteine. (**B**) ^1^H NMR spectra of a reaction mixture consisting of cysteine (1 mM), GSH (5 mM), and FA (1 mM, added last) over time at 298 K. *S*-hydroxymethyl-GSH (HMSGSH) and thioproline are indicated. (**C**) Time-dependent formation of thioproline and HMSGSH. Cysteine (1 mM), GSH (5 mM), and FA (1 mM). (**D**) Experimental design for metabolomics analysis. Quantification of thioproline (**E**), cysteine (**F**), and GSH (**G**) in A549 cells by LC-ESI-MS/MS {mean ± SEM, *n* = 5, one-way ANOVA [Tukey test for multiple comparison versus untreated (UT)]; for thioproline: *P = 0.0384; for cysteine: **P = 0.0095}. (**H**) Ratio of thioproline to cysteine in A549 cells exposed to 100 μM l-buthionine-sulfoximine (BSO) over 48 hours (mean ± SEM, *n* = 5, non-parametric *t* test, *P = 0.0270).

We next examined whether FA forms thioproline in cells cultured under standard laboratory conditions (Fig. 3D). To this end, we used a targeted metabolomics approach without thiol derivatization, as such strategies can perturb cysteine–aldehyde equilibria and affect the detection of thiazolidine species such as thioproline (*40*). By running chemical standards of GSH, cysteine, and thioproline, as well as a defined mixture of FA with GSH, we assigned peaks corresponding to these species in A549 cell extracts (fig. S4, B and C). A very low–intensity thioproline signal was also detected in the FA-GSH chemical mixture (fig. S4B), likely reflecting trace cysteine/cysteinyl equivalents generated during sample preparation or analysis, which can subsequently react with FA to form thioproline in vitro. Using this method, thioproline was robustly detected in A549 cells. Furthermore, metabolite quantification from chromatographic peak areas normalized to the number of cells collected in each sample revealed a significant increase of thioproline abundance following FA exposure (Fig. 3E). We then estimated intracellular cysteine and GSH levels from the same nonderivatized dataset, acknowledging that absolute values may be underestimated (Fig. 3, F and G). Exposure to 200 μM FA for 24 hours significantly increased cysteine levels, consistent with conditions that could support thioproline formation. In contrast, inhibition of GSH synthesis with BSO resulted in a marked depletion of GSH and was accompanied by a reduction in both total cysteine and thioproline levels (Fig. 3, E to G). Under conditions of GSH depletion, the relative amount of thioproline compared to cysteine was significantly increased (Fig. 3H), suggesting that endogenous FA can react with both thiols, with cysteine-FA conjugates remaining detectable under these conditions. Consistently, under our LC-MS conditions, we did not reliably detect HMSGSH as a reproducible chromatographic peak with matching retention time and MRM transitions relative to an in vitro reference (fig. S4B).

### FA induces ISR signaling in NRF2-hyperactive cancer cells

NRF2-active A549 cells become sensitized to FA upon erastin treatment; therefore, to define the transcriptional response to acute FA overload, we performed RNA sequencing on A549 cells treated with erastin for 48 hours, adding 200 μM FA during the final 24 hours. KEGG pathway analysis of dysregulated genes revealed alterations in multiple pathways, including p53 signaling and starvation response (Fig. 4, A and B, and fig. S5A). Notably, genes associated with the integrated stress response (ISR) (*18*, *19*) were up-regulated following combined treatment (Fig. 4C and table S2), correlating with a robust phosphorylation of eIF2α, a central hallmark of ISR activation (Fig. 4D).

**Fig. 4.**
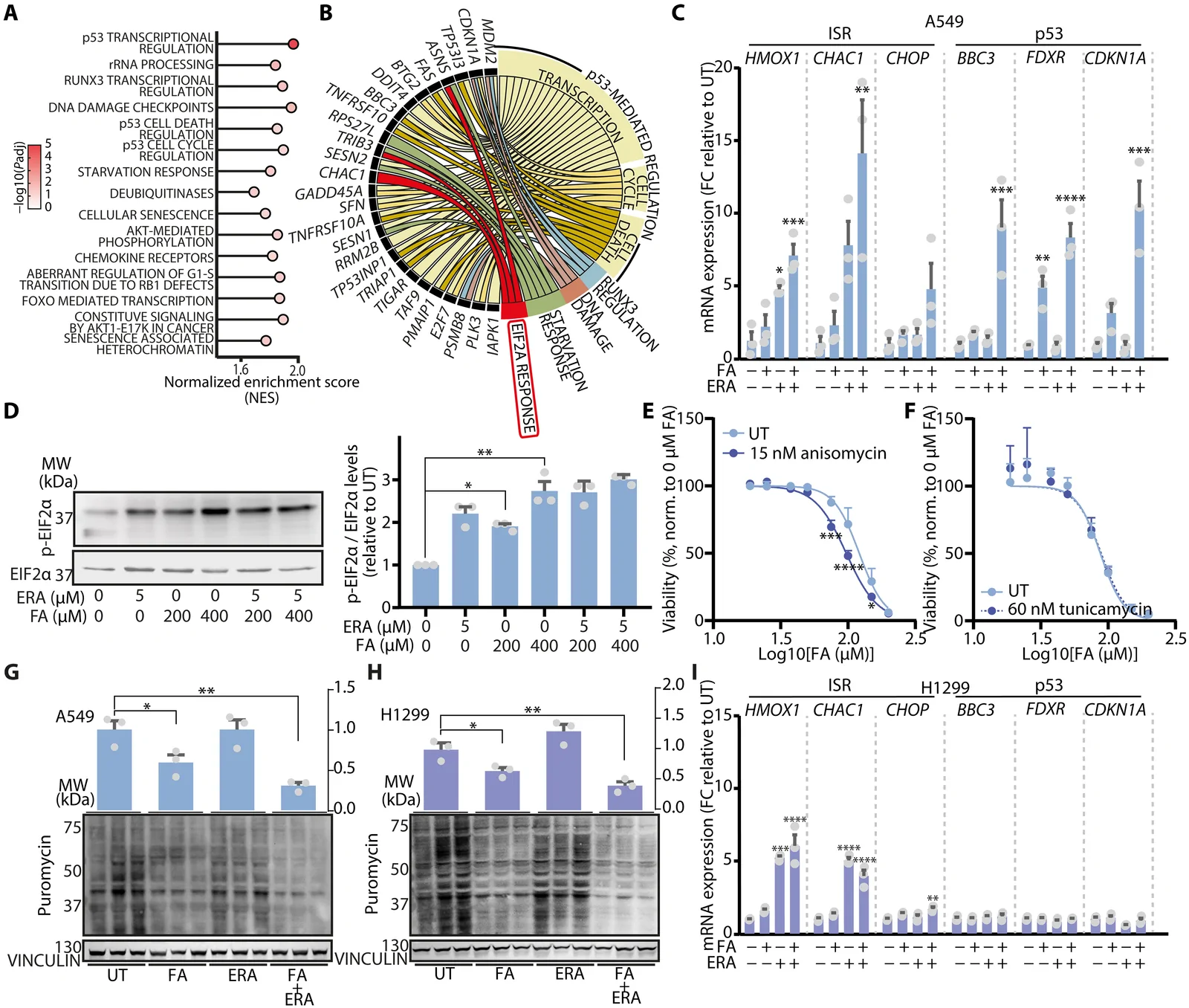
Formaldehyde triggers the integrated stress response in NRF2-active cells. (**A**) Gene Set Enrichment Analysis (GSEA) using KEGG in A549 cells exposed to erastin (ERA) (5 μM, 48 hours) and formaldehyde (FA) (200 μM, 24 hours). (**B**) Chord plot analysis showing significantly dysregulated genes between untreated (UT) and ERA+FA conditions from (A). (**C**) RT-qPCR of selected genes in the same conditions as (A) [mean ± SEM, *n* represented as dots and one-way ANOVA; for *HMOX1*: *P = 0.0132 and ***P = 0.0006; for *CHAC1*: **P = 0.0056; for *BBC3*: ***P = 0.0009; for *FDXR*: **P = 0.0056 (FA) and ****P < 0.0001 (FA + ERA); for *CDKN1A*: ***P = 0.0003]. (**D**) Left: Representative Western blot showing eIF2α phosphorylation in response to ERA (5 μM, 24 hours) and FA (200/400 μM, 4 hours) treatment. Right: Quantification {mean ± SEM, one-way ANOVA [nonparametric Kruskal-Wallis test versus untreated (UT)]; *P = 0.0358 and **P = 0.0063}. (**E**) FA tolerance in A549 in the presence of 15 nM anisomycin (mean ± SEM, *n* = 3 and two-way ANOVA; ***P = 0.0001; ****P < 0.0001; *P = 0.0238). (**F**) FA tolerance in A549 in the presence of 60 nM tunicamycin (mean ± SEM, *n* = 3). (**G**) FA and ERA effect on protein translation in A549 cells: 5 μM ERA for 24 hours adding 500 μM FA during the last 4 hours. Data are quantified in the top plot as ratio between puromycin and VINCULIN signal (mean ± SEM, *n* = 3, one-way ANOVA, *P = 0.0405, **P = 0.0022). (**H**) FA and ERA effect on protein translation in H1299 cells performed as in (G). Data are quantified in the top plot as described in (G) (mean ± SEM, *n* = 3, one-way ANOVA, *P = 0.0499; **P = 0.0032). (**I**) RT-qPCR of selected genes in H1299 cells [5 μM ERA, 48 hours; 200 μM FA, 24 hours (mean ± SEM, *n* = 3, one-way ANOVA, **P = 0.0051, ***P = 0.0001, ****P < 0.0001)].

ISR activation can arise from distinct upstream stress inputs, including endoplasmic reticulum (ER) stress, which engages the ISR via PERK activation, as well as ribosome-associated stress triggered by ribosome stalling and collisions (*41*). We first evaluated whether any of these stresses can alter the cellular tolerance to FA. To this end, we measured FA tolerance in cells treated with anisomycin, an agent that promotes ribosome collisions, or tunicamycin, an ER stressor that activates the ISR through PERK, using low doses to minimize confounding cytotoxicity (fig. S5B). Under these conditions, anisomycin markedly increased FA toxicity, whereas tunicamycin had no significant effect (Fig. 4, E and F). Accordingly, FA exposure impaired global protein translation (Fig. 4G) while erastin failed to halt protein synthesis despite inducing phosphorylation of eIF2α (Fig. 4, D and G). In summary, the translation arrest observed upon FA treatment is more consistent with ribosome stalling caused by FA-induced RNA-protein cross-links, a known cytotoxic lesion that triggers ribosome-associated stress responses if unresolved (*42*, *43*).

In support of a role for ribosome-associated stress signaling in the cellular response to FA, CRISPR screen data from a retinal pigment epithelial (RPE) cell model exposed to FA showed that genes involved in ribosome collision sensing and response, including *ZAK*, *MKK4*, *MAPK14*, and *GIGYF2*, are required for cell proliferation under FA stress (fig. S5C) (*44*). In cancer cells with intact NRF2 signaling (H1299), FA also blocked protein synthesis (Fig. 4H). However, it failed to up-regulate ISR-responsive genes, which were instead strongly induced by erastin (Fig. 4I), further supporting that FA-induced translation inhibition is independent of NRF2 activation status. Noncancerous cells do not constitutively activate NRF2 signaling; therefore, we set out to investigate whether endogenous FA selectively activates ISR in hematopoietic stem cells (HSCs) from a mouse model of endogenous FA accumulation (*10*). Analysis of scRNA-seq data revealed enrichment of p53 signaling, but not ISR gene expression, suggesting that in noncancerous cells, endogenous FA primarily elicits a p53-mediated response rather than ISR transcriptional program (fig. S5D).

### GSH is required for ferroptosis resistance in NRF2-hyperactive cancer cells

As GSH is minimally used for FA protection in A549 cells, we next asked whether NRF2-hyperactive cancer cells might also be less reliant on GSH for protection against ferroptosis. First, we performed a Pearson correlation analysis using all cell lines in the DepMap database to identify gene dependencies associated with high expression of *NFE2L2*, which encodes NRF2. Notably, *NFE2L2* expression negatively correlated with the dependency for growth on genes coding for GSH synthesis (*GCLC* and *GCLM*) and *GPX4* (Fig. 5, A and B), suggesting that NRF2-high cells may be less dependent on the canonical GSH-GPX4 axis for growth.

**Fig. 5.**
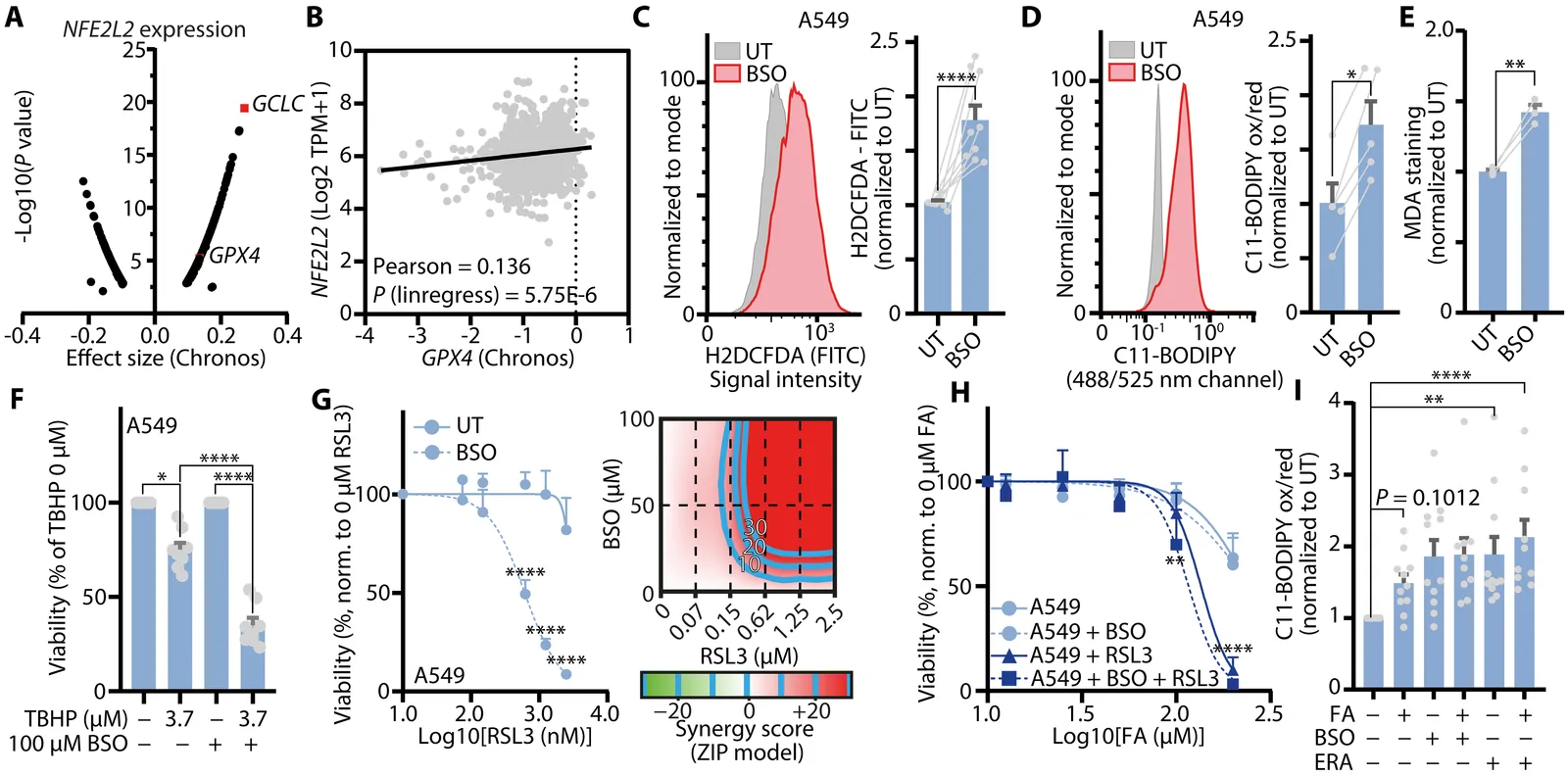
Distinct roles of glutathione in formaldehyde tolerance and ferroptosis resistance. (**A**) Pearson correlation comparing *NFE2L2* expression and gene dependency determined by Chronos score (DepMap) (cutoff *P* < 0.01). (**B**) Linear correlation between *NFE2L2* expression and gene dependency for *GPX4*. (**C**) Left: Histogram showing dichlorofluorescein (H_2_DCFDA) signal in A549 cells after 48 hours of 100 μM l-buthionine-sulfoximine (BSO), UT = untreated. Right: Quantification of H_2_DCFDA (mean ± SEM, *n* represented as dots and *t* test for comparison; ****P < 0.0001). (**D**) Left: C11-BODIPY 581/591 staining (oxidized channel) upon 48 hours 100 μM BSO. Right: Quantification of C11-BODIPY 581/591 as ratio between the signal in the 488/525 (oxidized) and in the 461/590 channel (reduced) [mean ± SEM, *n* represented as dots and *t* test for comparison; *P = 0.0333]. (**E**) Malondialdehyde (MDA) accumulation in A549 cells after 48 hours of 100 μM BSO exposure represented as ratio of the UT signal (mean ± SEM, *n* = 3, *t* test for comparison; **P = 0.0014). (**F**) Viability of A549 cells UT or exposed to *tert*-butyl-hydroperoxide (TBHP) in the presence or absence of 100 μM BSO [mean ± SEM, *n* represented as dots and one-way ANOVA (Tukey test for multiple comparison versus UT)]. (**G**) Left: Tolerance of A549 cells to the ferroptosis inducer RSL3 in the presence of 100 μM BSO [mean ± SEM, *n* = 3 and two-way ANOVA (Tukey test between UT and BSO at each point for multiple comparison); ****P < 0.0001]. Right: Zero interaction potency (ZIP) synergistic analysis of dose-response curves between RSL3 and BSO. (**H**) Formaldehyde (FA) tolerance of A549 cells in the presence of 1 μM RSL3 and/or 100 μM BSO [mean ± SEM, *n* = 3, two-way ANOVA (Tukey test for multiple comparison versus UT for each FA concentration); **P = 0.0011; ****P < 0.0001]. (**I**) C11-BODIPY 581/591 staining of A549 cells exposed to 100 μM BSO 72 hours, 5 μM erastin (ERA) 48 hours, and/or 200 μM FA 24 hours (one-way ANOVA, mean ± SEM, **P = 0.0025; ****P < 0.0001).

Given the well-established role of GSH in suppressing lipid peroxidation, we next directly tested whether GSH remains required for oxidative stress protection in NRF2-hyperactive cells. To this end, we depleted GSH in A549 cells using BSO and assessed multiple oxidative stress readouts. GSH depletion induced oxidation of the nonspecific oxidative stress probe dichlorofluorescein (H_2_DCFDA) (Fig. 5C), increased oxidation of the lipid peroxidation sensor C11-BODIPY (Fig. 5D), and induced accumulation of malondialdehyde (MDA), a terminal product of lipid peroxidation (Fig. 5E) (*45*). In contrast, CellROX fluorescence was not increased under conditions of GSH depletion (fig. S6A), indicating that loss of GSH does not elicit a global increase in freely diffusible ROS. Consistent with these findings, GSH depletion sensitized cells to the organic peroxide TBHP (Fig. 5F). Moreover, BSO synergized with the GPX4 inhibitor RSL3 to induce ferroptotic cell death (Fig. 5G). Notably, while BSO alone did not increase the toxicity of FA in A549 cells, RSL3 significantly enhanced FA toxicity, an effect that was not further exacerbated by GSH depletion (Fig. 5H). Last, we assessed lipid peroxidation using the C11-BODIPY probe following combined treatment with FA, erastin, and BSO. FA alone induced a modest increase in lipid peroxidation, whereas BSO or erastin alone produced a stronger effect. The most pronounced C11-BODIPY oxidation was observed upon combined treatment with erastin and FA, though the increase was not significant compared to erastin alone (Fig. 5I). Together, these results indicate that, while GSH is dispensable for FA detoxification in NRF2-hyperactive cells, it remains essential for ferroptosis resistance, with FA contributing to lipid peroxidation.

### Pharmacological inactivation of GSH synthesis and xCT impairs tumor growth

Our data indicate that SLC7A11 is required for FA protection, while GSH is essential for ferroptosis resistance in NRF2-hyperactive cells. We therefore hypothesized that simultaneous inhibition of GSH synthesis and cystine import would be lethal to these cells. To test this, we assessed whether cotreatment with BSO and erastin produced a synergistic toxic effect across multiple cancer cell lines in vitro (Fig. 6A and fig. S6, B and C). The combination impaired not only cells with *KEAP1-NFE2L2* alterations (A549 and T98G), but also *NFE2L2*–wild-type lines (Jurkat, H1299, and HCT116), consistent with previous reports describing that low *SLC7A11* expression underlies a synthetic lethality with GSH synthesis (*46*, *47*).

**Fig. 6.**
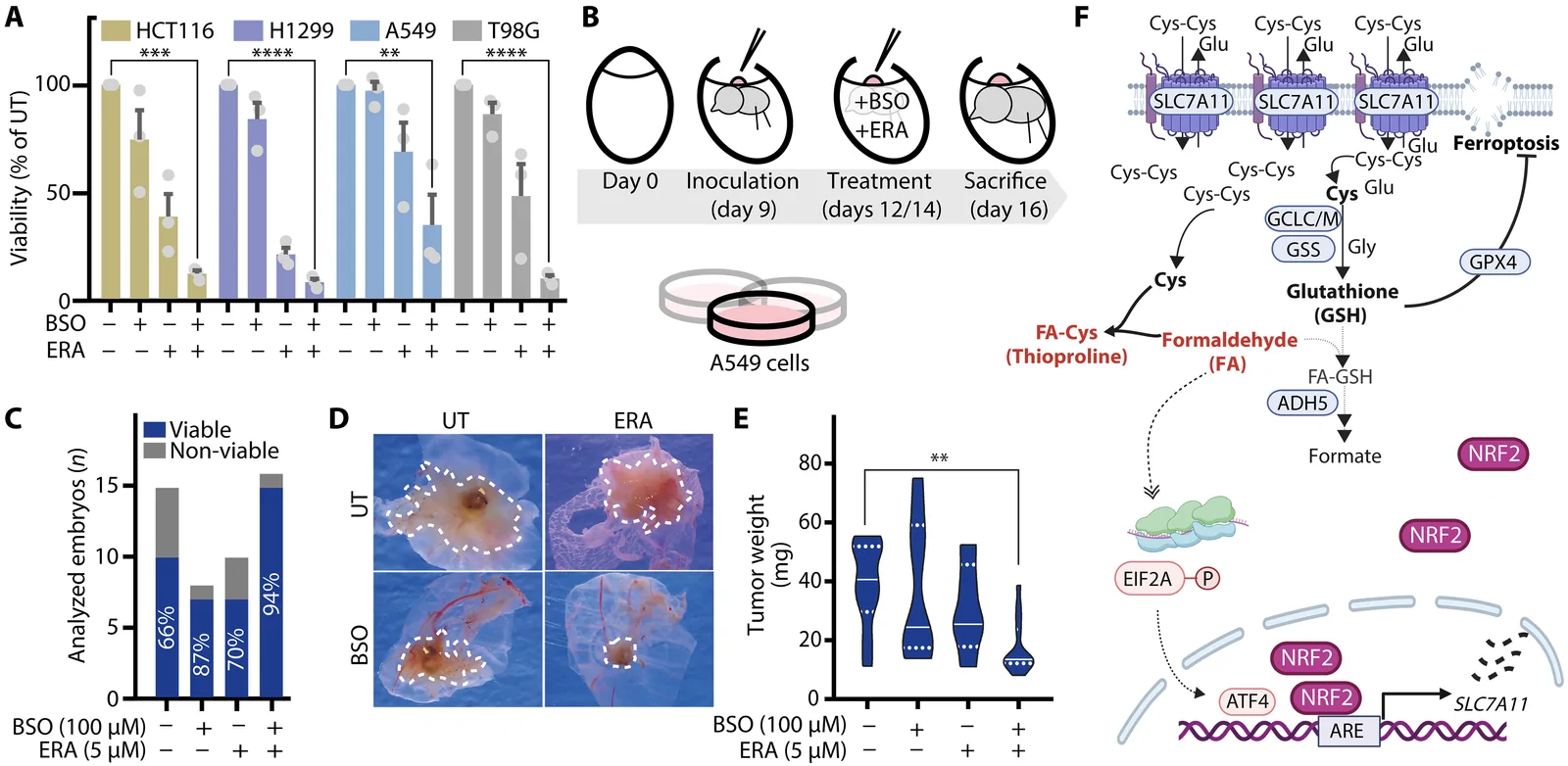
Pharmacological inhibition of GSH synthesis and SLC7A11 impairs tumor growth. (**A**) Viability of cell lines depicted in the figure exposed to 50 μM l-buthionine-sulfoximine (BSO) and/or 2.5 μM erastin (ERA) {mean ± SEM, *n* represented as dots and one-way ANOVA [Tukey test for multiple comparison versus untreated (UT)]; ***P = 0.0003 (HCT116), **P = 0.0044 (A549) and ****P < 0.0001 (H1299 and T98G)}. (**B**) Outline of the chicken chorioallantoic membrane (CAM) tumorigenesis assay. (**C**) Effect of BSO and ERA inoculation on the viability of embryos at day 16. (**D**) Representative images of CAM assay performed with A549 cells exposed to BSO and/or ERA. The tumors are highlighted by dotted lines. (**E**) Tumor weight determined at end point [*n* = 10 for UT, *n* = 7 for BSO, *n* = 5 for ERA, *n* = 14 for ERA+BSO and one-way ANOVA (Tukey test for multiple comparison); **P = 0.0087 (UT versus ERA+BSO)]. The median (solid white line) and the quartiles (dotted white line) are shown. (**F**) Model of formaldehyde (FA) protection in cells with hyperactivation of the KEAP1-NRF2 pathway. Created in BioRender. https://BioRender.com/ck9o6kk.

To evaluate this strategy in vivo, we used an animal-free tumorigenesis model based on the chorioallantoic membrane (CAM) of fertilized chicken embryos (Fig. 6B) (*48*). A549 cells embedded in basement matrix were implanted onto the CAM at embryonic day 9, and tumors were allowed to engraft and grow for 7 days. During this period, BSO and erastin were administered directly into the eggs, using concentrations that do not affect the viability of the embryos (Fig. 6C). On day 16, tumors were collected and weighed (Fig. 6, D and E). Consistent with our in vitro findings, combined BSO and erastin treatment significantly impaired tumor growth in ovo, further supporting the therapeutic relevance of targeting cystine uptake and GSH synthesis in NRF2-driven cancers.

## DISCUSSION

Endogenous FA is a highly reactive metabolite that threatens cell integrity by damaging proteins and nucleic acids, while also disrupting redox homeostasis (*1*, *4*–*6*). In the cytosol, FA detoxification is initiated through conjugation with GSH, a process that, while protective, may impose a metabolic burden on cancer cells that are already heavily reliant on GSH to counteract oxidative stress and prevent ferroptosis (*49*). Here, we show that the NRF2-dependent regulation of SLC7A11 reduces the dependency on GSH for FA protection. In these cells, FA conjugates with cysteine forming a thiazolidine known as thioproline, which is detected at steady state and accumulates under FA stress. This observation is consistent with sustained endogenous FA turnover (Fig. 6F). In a defined in vitro system, FA reacted with both GSH and cysteine; however, thioproline rapidly accumulated at the expense of HMSGSH (Fig. 3), indicating that cysteine-derived conjugate formation is favored under these conditions. Together, these data are consistent with a model in which FA reacts with multiple intracellular thiols, with the distribution of conjugates governed by thiol availability and intrinsic reaction kinetics.

Within this framework, SLC7A11-dependent cystine import emerges as a key determinant of FA tolerance independent from GSH. *SLC7A11*-deficient cells are more sensitive to FA than wild-type counterparts in standard growth conditions. However, under cystine deprivation, both *SLC7A11*-deficient and wild-type cells exhibit similarly increased sensitivity to FA, supporting a central role for SLC7A11-mediated cystine import in modulating FA tolerance. SLC7A11 activity may influence cellular FA tolerance through multiple, non–mutually exclusive processes, including increased intracellular cysteine availability (*16*), coupled glutamate export (*50*), and the requirement for reducing equivalents to convert cystine to cysteine (*51*).

Cells with hyperactive KEAP1-NRF2 signaling are primed for rapid activation of antioxidant and stress adaptation programs, including genes coregulated by NRF2 and activating transcription factor 4 (*18*). Combined treatment with FA and erastin—an SLC7A11 inhibitor—consistently induced the transcription of genes associated with the ISR, specifically in NRF2-active cells. In accordance with recent studies showing that RNA-protein cross-links trigger FA-associated stress signaling (*42*, *43*), we detected FA-dependent eIF2α phosphorylation, a marked inhibition of global protein synthesis, and found that anisomycin, which promotes ribosome collisions, exacerbated FA toxicity (Fig. 4). Together, these observations suggest that FA-induced ribosomal stress activates an ISR-linked transcriptional program. However, further studies are required to define the precise roles of the ISR in FA tolerance.

While GSH was barely required for FA protection in NRF2-hyperactive cells, it remained essential for ferroptosis resistance (Fig. 5). GSH depletion synergized with the ferroptosis inducer RSL3 causing cell death, in agreement with prior studies showing that *KEAP1*-mutant tumors rely on GSH to buffer ROS and promote NADH accumulation and disulfide stress as a consequence of SLC7A11 overexpression (*13*, *52*, *53*). Notably, our data indicated that RSL3—but not GSH depletion alone—increased FA toxicity. One possible explanation is that FA limits the availability of cysteine, which can function as an alternative cofactor for GPX4 (*54*), thus promoting lipid peroxidation by compromising cysteine-dependent antioxidant defenses.

Across our study, we have used FA as a stressor, despite its limited direct therapeutic relevance. To assess the translational potential of our findings, we conducted an in vivo assay using the CAM tumorigenesis model (*55*), which enables preclinical testing without the use of born animals. Consistent with our in vitro data and previous reports (*46*, *56*), combined use of BSO and erastin impaired tumor growth in this model. This effect is likely driven by a combined effect of disruption of redox homeostasis, cysteine availability, and glutamate accumulation, potentially exacerbating endogenous aldehyde stress. However, the relative contribution of FA toxicity to tumor death cannot be resolved from these data.

In summary, our data suggest that combined inhibition of GSH synthesis and cystine uptake may represent a therapeutically relevant strategy for tumors with heightened sensitivity to aldehyde stress, including cancers with DNA repair deficiencies such as BRCA1/2 mutation (*57*), as well as tumors with high *SLC7A11* expression, a feature often associated with poor prognosis in lung tumors and myeloid leukemia.

## MATERIALS AND METHODS

### Cell culture

HCT116 colon cancer cells were provided by G. Soria (originally from B. Vogelstein, Johns Hopkins University). A549 (RRID:CVCL_0023) and H1299 (RRID:CVCL_0060) cells were provided by C. Perez Castro (IBioBA-CONICET) and originally sourced from ATCC. T98G cells were provided by P. Menendez, originally from ATCC. These lines were cultured in Dulbecco’s modified Eagle’s medium (DMEM, Thermo Fisher Scientific, no. 12100061) supplemented with 10% fetal bovine serum (FBS, Natocor) and 1% penicillin/streptomycin (P/S, GIBCO, no. 15140-122). LUDLU-1 and H2170 were provided by M. Sanchez-Céspedes (IJC) originally from ECACC and ATCC. Nalm6 cells and Jurkat cells were obtained from DSMZ, while K562 were sourced from ATCC. These lines were maintained in RPMI 1640 medium (Thermo Fisher Scientific, no. 61870044) with 10% FBS and 1% P/S. All cells were cultured at 37°C, 5% CO_2_ and tested regularly for mycoplasma contamination. Experiments were approved by the ethics committee from Hospital Universitario Germans Trias i Pujol, Protocol ID: PI-24-098.

### Cell line generation

For generation of CRISPR-Cas9 knockout cell lines, the vector lenticrispv2 (Addgene catalog no. 52961) was used. The sgRNAs are listed in table S1. For *GSS*- and *SLC7A11*-deficient cells, we selected puromycin-resistant cells, validated the down-regulation of the target proteins by Western blot, and performed the viability assays with the pool of puromycin-resistant cells. *ADH5*-deficient cells were generated in A549 cells stably expressing Cas9 (Addgene, catalog no. 78546) infected with a lentiviral vector coding for an sgRNA against the exon 3 of *ADH*5 previously published (*2*). Clonal dilution was performed, and the cells showing down-regulation of ADH5 were selected for the viability assays. For the complementation of *SLC7A11*-deficient cells, we cloned the cDNA of *SLC7A11* introducing silent mutations that prevent the recognition of the sgRNA in a pLOC lentiviral backbone harboring a resistance marker for blasticidin. The expression of SLC7A11 was validated in the pool of blasticidin-resistant cells by Western blot.

### Chemical treatments

Cells were treated with the following compounds: FA (Thermo Fisher Scientific, no. 28906), TBHP (Sigma-Aldrich, no. 458139), RSL3 (MedChemExpress, no. HY-100218A), BSO (Sigma-Aldrich, no. B2515), erastin (MedChemExpress, no. HY-15763), sulfasalazine (MedChemExpress, no. HY-14655), *N*-acetyl-l-cysteine (NAC, Sigma-Aldrich, no. A7250), cystine (Thermo Fisher Scientific, no. J61661.09), anisomycin (Cayman Chemical, no. 11308), and tunicamycin (Cayman Chemical, no. 11445). The concentrations and duration of each treatment are described in the corresponding figure.

### Oxidative stress detection

A549 cells were seeded in triplicate at 1 × 10^4^ cells/mL in complete DMEM and incubated overnight at 37°C, 5% CO_2_. Cells were treated with 100 μM BSO for 48 hours, then incubated with 10 μM H_2_DCFDA (Thermo Fisher Scientific, no. D399) or 5 μM C11-BODIPY (no. D3861) for 30 min at 37°C in the dark. For CellROX (Thermo Fisher Scientific, no. C10422), 500 μM TBHP was applied for 20 min as positive control. After washing with phosphate-buffered saline (PBS) + 2% FBS, cells were trypsinized and transferred to 96-well round-bottom plates or cytometry tubes. Fluorescence was detected by flow cytometry [FITC channel: Ex 488 nm, Em 517 to 527 nm; PE channel: Ex 488 nm, Em 576/26; or APC (allophycocyanin) channel: Ex 640 nm, Em 665 nm, for CellROX] in a BD FACSCanto II.

For MDA detection, A549 cells were seeded in triplicate (4 × 10^5^ cells per 10-cm dish) in complete DMEM and incubated at 37°C, 5% CO_2_ overnight. The following day, cells were treated with 100 μM BSO for 48 hours. Then, cells were harvested using trypsin and centrifuged (1000*g*, 5 min). Immediately after, pellets were lysed in lysis buffer (20 mM tris-HCl, pH 7.5, 150 mM NaCl, 0.5 mM EDTA, and 1% Triton X-100) and centrifuged (4°C, 12,000*g*, 15 min), and the supernatant was used for MDA detection, following the manufacturer’s instructions (ApexBIO, no. K2167). Fluorescence (Ex/Em 535/590 nm) was measured (Synergy H1 microplate reader).

### Western blotting

Cells were plated in six-well plates (4 × 10^5^ cells per well) and treated 24 hours later. Cells were harvested using trypsin and centrifuged (4°C, 1000*g*, 5 min), washed with PBS, frozen in liquid nitrogen, and stored at −80°C. Pellets were lysed with buffer [0.25 M tris-HCl, pH 6.8, 8% SDS, 40% glycerol, protease inhibitor (Sigma-Aldrich, no. P2714-1BTL), and phosphatase inhibitor (Santa Cruz Biotechnology, no. sc-45065)] and sonicated (Bioruptor, Diagenode, 10 cycles 30 s on/30 s off). Samples were quantified by BCA assay (Thermo Fisher Scientific, no. A55861). Extracts were heated at 90°C for 5 min, centrifuged (13,000*g*, 5 min), and supplemented with β-mercaptoethanol and bromophenol blue. Then, samples were resolved in 10 to 15% SDS-PAGE gels and transferred to 0.45 μm nitrocellulose membranes (Thermo Fisher Scientific, no. 88018). Blocking was performed using 2.5% milk or bovine serum albumin in TBS. Membranes were incubated with primary antibodies overnight at 4°C and then with horseradish peroxidase (HRP)– or fluorescent-conjugated secondary antibodies. Detection was performed with ECL (Amersham, no. 2232; Thermo Fisher Scientific, no. 34580) and imaged using a GBOX (Syngene) or Odyssey (LI-COR). The following antibodies were used across this work: Anti-SLC7A11/xCT (Abcam, catalog no. ab307601; RRID: AB_3094570), Anti-GCLC (H-5) (Santa Cruz Biotechnology, catalog no. sc-390811; RRID: AB_2736837), Anti-GCLM (G-4) (Santa Cruz Biotechnology, catalog no. sc-55585; RRID: AB_831788), Anti-ADH5 (2D11) (Santa Cruz Biotechnology, catalog no. sc-293460; RRID: AB_2927735), Anti–α-Tubulin (DM1A) (Santa Cruz Biotechnology catalog no. sc-32293 HRP; RRID: AB_628412), Anti-EIF2S1 (A42:F421) (Proteintech, catalog no. 11170-1-AP; RRID: AB_2096489), Anti–p-EIF2S1 (Ser51) (Proteintech, catalog no. 68023-1-Ig; RRID: AB_2918767), Anti-GSS (OTI2F2) (OriGene, catalog no. TA501936; RRID: AB_11128188), Anti-NRF2 (Proteintech, catalog no. 16396-1-AP; RRID: AB_2782956), Anti-Vinculin (7F9) (Santa Cruz Biotechnology, catalog no. sc-73614 HRP; RRID: AB_1131294), Anti-GAPDH (6C5) (Abcam, catalog no. ab8245; RRID: AB_2107448), Anti-Puromycin (12D10) (Merck, catalog no. MABE343; RRID: AB_2566826), Anti-FLAG (M2) (Merck, catalog no. F3165; RRID: AB_259529), IRDye 680RD Goat anti-Ms (LI-COR, catalog no. 926-68070; RRID: AB_10956588), IRDye 800CW Donkey anti-Rb (LI-COR, catalog no. 926-32213; RRID: AB_621848), Anti-Rabbit IgG-HRP (Merck, catalog no. A0545; RRID: AB_257896), and Anti-Mouse IgG-HRP (Merck, catalog no. A9044; RRID: AB_2313567).

### qPCR

RNA was extracted using the RNeasy Mini Kit (Qiagen, no. 74014), quantified (Nanodrop Lite), and reverse transcribed with SuperScript III (Thermo Fisher Scientific, no. 18080-044) and Oligo-dT primers. qPCR was performed using SYBR master mix (PB-L, no. 0501) in triplicates on a CFX96 system (Bio-Rad). Expression was calculated via the 2^−ΔΔCt^ method. Primer sequences are listed in table S1.

### Open reading frame cloning

Human *SLC7A11* was amplified from cDNA prepared from A549 exposed to erastin with primers hSLC7A11-Fw and hSLC7A11-Rv and cloned into pLOC vector opened with Bam HI using Hi-Fi mix. Cloning was validated by sequencing and by Western blot. Human *KEAP1* was amplified from cDNA prepared from H1299 cells with primers hKEAP1-Fw and hKEAP1-Rv and cloned into pLOC vector opened with Bam HI including the sequence coding for FLAG epitope using Hi-Fi mix. Cloning was validated by sequencing and Western blot. For cloning the pLOC expression vector (Horizon discovery, catalog no. OHS5832) was used.

### RNA sequencing and scRNA-seq analysis

A549 cells were seeded (1 × 10^6^ and 1.5 × 10^6^ cells per 10-cm dish) and treated under the following conditions: untreated, +FA (200 μM 24 hours), +erastin (5 μM 48 hours), and +FA/erastin. RNA was isolated (RNeasy Mini Kit), quantified, and prepared at 100 ng/μL. Biological replicates were prepared. Samples were then sequenced on a NovaSeq system using the TruSeq mRNA library (Macrogen). FASTQ files were processed using the RNA-seq platform and differential expression was performed following DEseq pipeline in R environment. The resulting list of significantly differentially expressed genes was subjected to gene set enrichment analysis using the fgsea package, and plotted in volcano plots using ggplot2 package and as a chord plot by using the GOplot package in R.

For scRNA-seq, raw reads were downloaded from GSE157832. Transcript quantification and generation of count matrices were performed using the scrnaseq Nextflow pipeline provided by nf-core, implementing the Salmon Alevin method with the GRCm39 transcriptome as the reference genome. Potential doublet cells were identified and removed using the Scrublet algorithm within the Scanpy Python framework. Quality control steps involved filtering cells to retain only those with less than 10% mitochondrial reads, at least 1200 detected genes, and total UMI counts falling within three standard deviations of the mean. Following this initial filtering, genes expressed in fewer than 10 cells were removed from the dataset. Subsequent clustering was performed using the Leiden clustering algorithm, and dimensionality reduction for visualization was achieved through Uniform Manifold Approximation and Projection, both implemented in Scanpy. For differential expression analysis, cells belonging to the HSC cluster were identified by high expression of Procr. These cells were grouped into three pseudobulk replicates per genotype. Differential gene expression was then assessed using the DESeq2 package in R. It should be noted that the pseudobulk approach does not fully capture biological variability, as the replicates used are technical pseudoreplicates rather than true biological replicates. Genes significantly differentially expressed were defined as those with an adjusted *P* value (*P*-adj) < 0.05 and absolute log2 fold change (log2FC) ≥ 0.5. The resulting list of significantly differentially expressed genes was subjected to gene set enrichment analysis using ShinyGO 0.85.1, KEGG database, with an FDR cutoff of 0.01, and using pathway database and redundancy removal for filtering.

### Cell viability and proliferation assays

Cells were seeded in 96-well plates at 3000 cells per well and treated with preincubated compounds (NAC, BSO, or erastin), followed by stressors (FA, RSL3, and TBHP). After 72 hours, resazurin (Sigma-Aldrich, no. 199303) was added to a final concentration of 40 μM. After 2 to 3 hours, fluorescence (Ex/Em = 575/590 nm) was measured (Enspire, Perkin Elmer). For colony formation assays, 600 cells per well were plated in six-well plates, treated, and incubated for 7 to 10 days. Cells were fixed and stained with 0.5% crystal violet (ChemCruz, no. sc-207460) and 6% glutaraldehyde (Sigma-Aldrich, no. G6275). For spheroid formation, 96-well plates were precoated with 1.5% agarose. Cells were plated with treatments, incubated 5 to 7 days, and imaged using a Canon Rebel T3i camera and Zeiss Axio A1 microscope.

For anisomycin and tunicamycin cell viability assays, A549 cells were seeded in 96-well plates at 2000 cells per well in complete DMEM and incubated at 37°C, 5% CO_2_ overnight. The following day, cells were treated with anisomycin 30 nM (Cayman Chemical, no. 11308), or tunicamycin 120 nM (Cayman Chemical, no. 11445). The next day, cells were treated with FA so that anisomycin and tunicamycin concentrations were diluted to 15 and 60 nM, respectively. After 72 hours, resazurin (Sigma-Aldrich, no. 199303) was added to a final concentration of 30 μM. After 2 to 3 hours, fluorescence (Ex/Em = 575/590 nm) was measured (Synergy H1 microplate reader).

The viability measure in the absence of cystine was performed in cystine-deprived DMEM (Thermo Fisher Scientific, no. 21013024) supplemented with 200 μM methionine (Sigma-Aldrich, no. M9625), 1× GlutaMAX (Thermo Fisher Scientific, no. 35050061), 1% P/S, and 10% FBS. Briefly, A549 cells were seeded at 2000 cells per well in either complete DMEM (the above-mentioned media plus 200 μM cystine) or cystine-deprived DMEM and incubated at 37°C, 5% CO_2_ overnight. The following day, cells were treated with FA in the same DMEM cystine-rich or cystine-deprived conditions. After 72 hours, resazurin (Sigma-Aldrich, no. 199303) was added to a final concentration of 30 μM. After 2 to 3 hours, fluorescence (Ex/Em = 575/590 nm) was measured (Synergy H1 microplate reader).

### Cystine uptake assay

Cystine uptake was assessed using the Cystine Uptake Assay Kit (Dojindo, no. UP05-10). Cells (1 × 10^4^ per well) were seeded in 96-well plates. After 24 hours, cells were washed and incubated with selenocystine for 30 min. After fixation, a fluorescent probe was added and read (Ex/Em = 490/535 nm) on an Enspire microplate reader.

### GSH measurements

GSH levels were assessed using the GSH-Glo Glutathione kit (Promega, no. V6911). Briefly, cells were plated (3 × 10^3^ per well for HCT116, H1299 and T98G cells; 2.5 × 10^3^ per well for A549 cells) in 96-well plates. After 24 hours, BSO was added. Then, after 72 hours, culture medium was removed and cells were incubated with 100 μL of 1× GSH-Glo Reagent for 30 min. Reconstituted Luciferin Detection Reagent (100 μL) was added to each well and incubated for 15 min. Last, supernatants were transferred to a white 96-well plate and luminescence was measured on an Enspire microplate reader. Results were normalized to viable cell number using a duplicated plate that was counted with trypan blue. GSH concentrations were calculated as relative to each cell line and by interpolation from a GSH standard curve.

### Preparation of samples for LC-ESI-MS/MS analysis

A549 cells were seeded at 7.5 × 10^5^ cells per 10-cm dish in complete DMEM and incubated at 37°C, 5% CO_2_ overnight. The following day, A549 wild-type cells were treated with 100 μM BSO, and the next day, they were treated with FA (200 μM). The day after, cells were washed with PBS, trypsinized, and counted. Immediately after, cells were washed twice with PBS and pellets were rapidly frozen by direct immersion in liquid nitrogen and stored at −80°C. Cell pellets (in 1.5-mL microcentrifuge tubes, 1.6 × 10^6^ to 4.6 × 10^6^ cells per sample) were lysed by the addition of 80% v/v methanol in H_2_O (500 μL) and incubation on ice for 15 min. The samples then underwent centrifugation (14,000*g*, 10 min at 4°C), and the resulting supernatants were transferred to UPLC vials for MS analysis.

### LC-ESI-MS/MS analysis

The LC-ESI-MS/MS experiments consisted of a Waters Acquity I Class Plus Series UPLC, comprising a binary solvent manager plus sample manager fitted with a flow-through needle connected to a Xevo TQ-XS tandem quadrupole mass spectrometer with an electrospray interface (Waters Ltd). The temperature of the electrospray source was maintained at 150°C, and the desolvation temperature was maintained at 600°C. Nitrogen gas was used as the desolvation gas (1000 liters/hour) and the cone gas flow was set to 150 L/h. The nebulizer gas flow pressure was set at 7.0 bar. The capillary voltage was set at 3.15 kV, and the cone voltage was maintained at 25 V. A 2-μL aliquot of the extracted cell sample (250 μL) was injected onto an Acquity UPLC BEH C18 (2.1 × 100 mm, 1.7 μm) column (Waters Ltd., Manchester, UK). The column was eluted using a gradient with solvent A, 0.1% formic acid and solvent B, acetonitrile (containing 0.1% formic acid) at a flow rate of 0.4 mL/min with a run time of 5.5 min. The following gradient was used: 0 min—1% B, 1 min—1% B, 4.6 min—5% B, 4.7 min—50% B, 5.0 min—50% B, 5.1 min—1% B, and 5.5 min—1% B. The column temperature was set at 40°C using the Acquity column manager, and the temperature of the samples was maintained at 6°C in the sample manager. The samples were analyzed in positive electrospray ionization mode using multiple reaction monitoring (MRM). The MRM transitions monitored plus collision energies used were as follows: l-cysteine 122 to 76 *m/z* (mass/charge ratio; quantifier, 12 eV) plus 122 to 105 *m/z* (qualifier, 12 eV), thioproline 134 to 88 *m/z* (quantifier, 16 eV) plus 134 to 105 *m/z* (qualifier, 16 eV), GSH 308 to 162 *m/z* (quantifier, 16 eV) plus 308 to 179 *m/z* (qualifier, 16 eV), and HMSGSH 337 to 179 *m/z* (quantifier, 16 eV) plus 337 to 162 *m/z* (qualifier, 16 eV). The collision gas was argon with a flow rate of 0.15 mL/min (indicated cell pressure 3.5 × 10^−3^ mbar). The resolution was 0.75 *m/z* unit at peak base. The optimal MRM transition collision energy values were obtained by acquiring MS/MS data following the fluidic infusion of pure standards for each compound apart from HMSGSH, which was a standard produced by the reaction of GSH with FA. The data were acquired and processed using MassLynx V4.2 software. Metabolite concentrations obtained from targeted metabolomics (nmol/mL) were normalized to the number of cells used for extraction and expressed as nanomoles per 10^6^ cells.

### NMR analyses

All spectra were recorded on an 800-MHz Bruker Avance NOE spectrometer using a 5-mm TXO cryoprobe and operating at 298 K. Samples were prepared in microcentrifuge tubes before being transferred into 5-mm-diameter NMR tubes for NMR analysis. All NMR data were processed and analyzed using Topspin version 4.4.1. ^1^H NMR time-course spectra were collected as pseudo 2D experiments, with each data point having a 12-s acquisition time. Signal intensities are calibrated to the intensity of the ^1^H resonance of 3-(trimethylsilyl)propionic-2,2,3,3-d_4_ acid (TSP), which was added as an internal standard. The concentrations of GSH, cysteine, and FA were selected to ensure reliable detection and quantification of all species in the NMR spectra, while allowing assessment of intrinsic reaction kinetics under defined conditions, rather than to recapitulate exact intracellular concentrations.

Reaction mixtures were prepared as follows, see below. 

#### GSH + 0.2 eq. cysteine +0.2 eq. FA

1) 30 μL of a 100 mM GSH in 100 mM sodium phosphate buffer in H_2_O pH 7.4

2) 6 μL of 100 mM cysteine in 100 mM sodium phosphate buffer in H_2_O pH 7.4

3) 6 μL of a 100 mM FA stock in H_2_O

4) 5 μL of a 10 mg/mL stock of TSP in D_2_O

5) 60 μL of D_2_O

6) 499 μL of 100 mM sodium phosphate buffer in H_2_O pH 7.4

The sample was then immediately subjected to NMR analysis.

#### Competition experiments after FA and GSH prereaction

A master reaction mixture was first prepared as follows:

1) 210 μL of 100 mM GSH in 100 mM sodium phosphate buffer in H_2_O pH 7.4

2) 42 μL of a 100 mM FA stock in H_2_O

3) 35 μL of a 10 mg/mL stock of TSP in D_2_O

4) 420 μL of D_2_O

5) 3283 μL of 100 mM sodium phosphate buffer in H_2_O pH 7.4

After the addition of FA (added last), 570 μL of the master stock was removed and combined with 30 μL of 100 mM sodium phosphate buffer in H_2_O pH 7.4. The sample was then immediately subjected to NMR analysis.

For analysis following the addition of cysteine, aliquots of the master stock (570 μL) were taken and combined (at different time-points) with preprepared solutions as follows:

1) Six microliters of 100 mM cysteine in 100 mM sodium phosphate buffer in H_2_O pH 7.4; 24 μL of 100 mM sodium phosphate buffer in H_2_O pH 7.4

2) Twelve microliters of 100 mM cysteine in 100 mM sodium phosphate buffer in H_2_O pH 7.4; 18 μL of 100 mM sodium phosphate buffer in H_2_O pH 7.4

Each combined sample was then immediately subjected to NMR analysis.

### CAM assay

Fertilized eggs (Granja Santa Isabel, Córdoba, Spain) were incubated for 9 days at 37°C, 55% humidity. A window was created on day 9, and a drop of P/S was applied. A549 cells (2 × 10^6^ cells/mL) were mixed 1:1 with Matrigel (Cultek, no. 354234), incubated, and 50 μL was placed onto the CAM within a containment ring. On days 12 and 14, 50-μL drops containing treatments (BSO or erastin) were applied. On day 16, embryos were euthanized, and tumors were excised, imaged, and weighed.

### Statistical analysis

Data were analyzed using Prism 10 (GraphPad). Analysis of variance (ANOVA; one-way or two-way) tests were applied as described in the legend of the corresponding figure. Data are shown as mean ± SEM, with dots corresponding to independent measurements from samples grown or collected from different plates.

## Supplementary Materials

### The PDF file includes:

Figs. S1 to S6 Legends for tables S1 and S2 Uncropped western blot images References
