# Supplementary material for: NRF2 coordinates cystine import and glutathione use to sustain ferroptosis resistance and formaldehyde tolerance in cancer
Source: Sci Adv. 2026 Oct 23;12(43):eaea1431. doi: 10.1126/sciadv.aea1431 (PMC13693651; doi:10.1126/sciadv.aea1431)
Supplement: Supplementary file 1 [file sciadv.aea1431_sm.pdf]

Science Advances
AAAS

Supplementary Materials for

NRF2 coordinates cystine import and glutathione use to sustain ferroptosis resistance and formaldehyde tolerance in cancer

Agustín E. Morellato *et al.*

Corresponding author: Lucas B. Pontel, lpontel@carrerasresearch.org

Sci. Adv. **12**, eaea1431 (2026)
DOI: 10.1126/sciadv.aea1431

The PDF file includes:

Figs. S1 to S6
Legends for tables S1 and S2
Uncropped western blot images
References

Other Supplementary Material for this manuscript includes the following:

Tables S1 and S2

Fig. S1

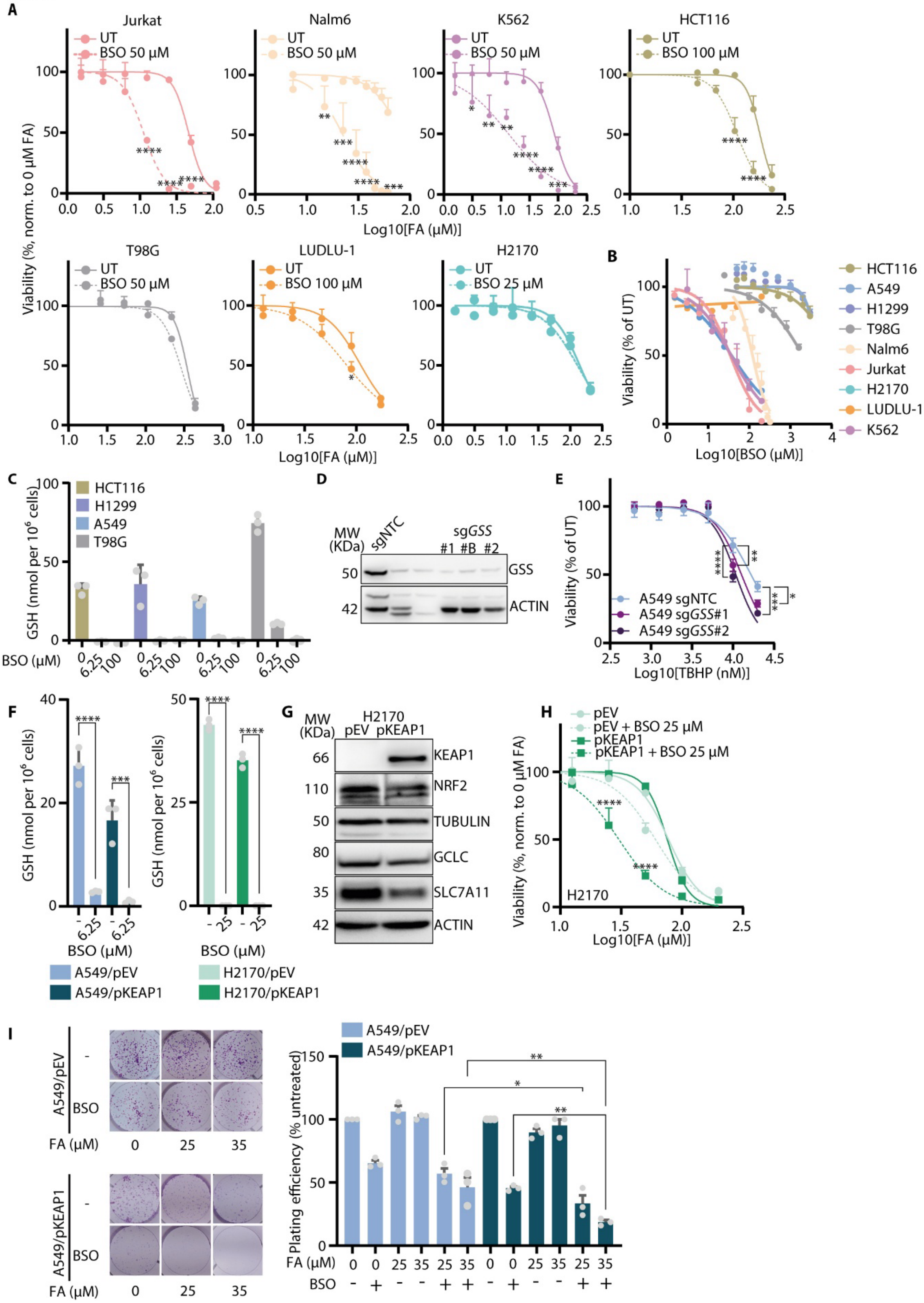

A
Jurkat
Nalm6
K562
HCT116
UT
BSO 50 μM
BSO 100 μM
Viability (%, norm. to 0 μM FA)
Log10[FA (μM)]
T98G
LUDLU-1
H2170
BSO 25 μM
B
Viability (% of UT)
Log10[BSO (μM)]
HCT116
A549
H1299
T98G
Nalm6
Jurkat
H2170
LUDLU-1
K562
C
GSH (nmol per 10^6 cells)
BSO (μM)
D
MW (KDa)
sgNTC
sgGSS
#1 #B #2
GSS
ACTIN
E
Viability (% of UT)
Log10[TBHP (nM)]
A549 sgNTC
A549 sgGSS#1
A549 sgGSS#2
F
GSH (nmol per 10^6 cells)
BSO (μM)
A549/pEV
A549/pKEAP1
H2170/pEV
H2170/pKEAP1
G
H2170
pEV pKEAP1
KEAP1
NRF2
TUBULIN
GCLC
SLC7A11
ACTIN
H
pEV
pEV + BSO 25 μM
pKEAP1
pKEAP1 + BSO 25 μM
Viability (%, norm. to 0 μM FA)
Log10[FA (μM)]
H2170
I
A549/pEV
A549/pKEAP1
BSO
FA (μM)
Plating efficiency (% untreated)

Fig. S1. Cancer cells cope with formaldehyde through glutathione-dependent and independent mechanisms. A. Viability plots for the depicted cell lines exposed to increasing concentrations of formaldehyde (FA). These plots were used to estimate the lethal dose that causes 50% death, which were plotted in the heatmap shown in Fig. 1B. **B.** Viability curves for the depicted cell lines exposed to increasing concentrations of L-buthionine sulfoximine (BSO) over 3-4 days. Viability was assessed by resazurin metabolism as described in the Methods section. **C.** Glutathione (GSH) determination by using the GSH-Glo kit in HCT116, A549, H1299, and T98G cells exposed to 6.25 and 100 μM BSO for 72 h (n=3). **D.** Western blot of A549 cells infected with sgRNA targeting *GSS*, or a non-targeting control (NTC). **E.** Viability curves for the depicted cell lines exposed to increasing concentrations of tert-butyl-hydroperoxide (TBHP) over 3-4 days [mean±SD, n=3, one-way ANOVA analysis (Tukey test for multiple comparisons); **p=0.0052, *p=0.0154, ***p=0.0004, ****p<0.0001]. **F.** GSH quantification by using the GSH-Glo kit in A549 and H2170 cells complemented with KEAP1 (pKEAP1) and their isogenic control carrying the empty vector (pEV) in the presence of the indicated concentrations of BSO over 48 h. **G.** Immunoblots showing the expression of the labeled proteins in H2170 cells stably carrying an empty vector (pEV) or a vector coding for wild-type KEAP1 (pKEAP1). **H.** Viability curves for H2170/pEV or H2170/pKEAP1 cells exposed to different concentrations of FA in the presence of BSO (25 μM) [mean±SD, n=3 and two-way ANOVA analysis (Tukey test between UT and BSO 25 μM at each point for multiple comparisons); ****p<0.0001]. **I.** Colony survival assay (CSA) for A549/pEV and A549/pKEAP1 in the presence of FA and/or BSO (6.25 μM) [mean±SEM, n=3, one-way ANOVA analysis (Tukey test for multiple comparisons); **p=0.0018, *p=0.0110, **p=0.0025].

Fig. S2

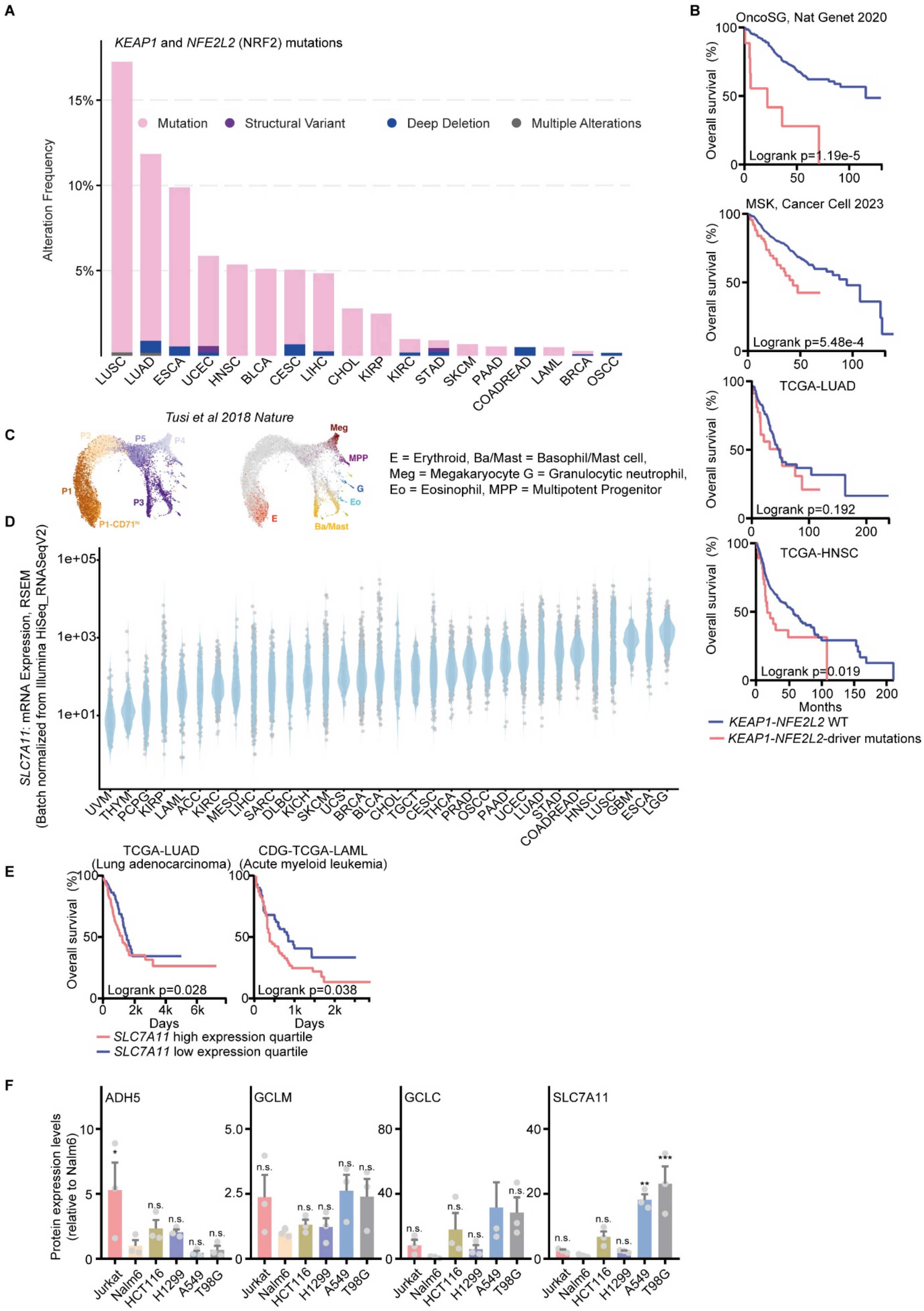
A
KEAP1 and NFE2L2 (NRF2) mutations
Alteration Frequency
Mutation
Structural Variant
Deep Deletion
Multiple Alterations
LUSC LUAD ESCA UCEC HNSC BLCA CESC LIHC CHOL KIRP KIRC STAD SKCM PAAD COADREAD LAML BRCA OSCC
B
OncoSG, Nat Genet 2020
Logrank p=1.19e-5
MSK, Cancer Cell 2023
Logrank p=5.48e-4
TCGA-LUAD
Logrank p=0.192
TCGA-HNSC
Logrank p=0.019
Overall survival (%)
Months
KEAP1-NFE2L2 WT
KEAP1-NFE2L2-driver mutations
C
Tusi et al 2018 Nature
E = Erythroid, Ba/Mast = Basophil/Mast cell,
Meg = Megakaryocyte G = Granulocytic neutrophil,
Eo = Eosinophil, MPP = Multipotent Progenitor
D
SLC7A11: mRNA Expression, RSEM
(Batch normalized from Illumina HiSeq_RNASeqV2)
UVM THYM PCPG KIRP LAML ACC KIRC MESO LIHC SARC DLBC KICH SKCM UCS BRCA BLCA CHOL TGCT CESC THCA PRAD OSCC PAAD UCEC LUAD STAD COADREAD HNSC LUSC GBM ESCA LGG
E
TCGA-LUAD
(Lung adenocarcinoma)
Logrank p=0.028
CDG-TCGA-LAML
(Acute myeloid leukemia)
Logrank p=0.038
Overall survival (%)
Days
SLC7A11 high expression quartile
SLC7A11 low expression quartile
F
ADH5
GCLM
GCLC
SLC7A11
Protein expression levels
(relative to Nalm6)
Jurkat Nalm6 HCT116 H1299 A549 T98G

Fig. S2. SLC7A11 expression levels across normal and tumor cells. A. Alteration frequency of either *KEAP1* or *NFE2L2* (NRF2) in tumors from TCGA cohorts. **B.** Kaplan-Meier plots in lung tumors: Lung Adenocarcinoma (OncoSG, Nat Genet 2020) (*59*); Lung Adenocarcinoma (MSK, Cancer Cell 2023) (*60*); TCGA Lung Adenocarcinoma (LUAD) data; and TCGA head and neck squamous cell carcinoma (HNSC). **C.** Cell distribution in data from mouse hematopoietic progenitor cells (HPC) from Tusi et al. (*58*) (Reference for Fig. 2E). **D.** *SLC7A11* mRNA expression represented as RSEM. Data obtained from TCGA curated cohorts through cBioPortal: ACC Adrenocortical carcinoma, BLCA Bladder urothelial carcinoma, BRCA Breast invasive carcinoma, CESC Cervical squamous cell carcinoma and endocervical adenocarcinoma, CHOL Cholangiocarcinoma, COAD Colon adenocarcinoma, DLBC Diffuse large B-cell lymphoma, ESCA Esophageal carcinoma, GBM Glioblastoma multiforme, HNSC Head and neck squamous cell carcinoma, KICH Kidney chromophobe, KIRC Kidney renal clear cell carcinoma, KIRP Kidney renal papillary cell carcinoma, LAML Acute myeloid leukemia, LGG Brain lower-grade glioma, LIHC Liver hepatocellular carcinoma, LUAD Lung adenocarcinoma, LUSC Lung squamous cell carcinoma, MESO Mesothelioma, OV Ovarian serous cystadenocarcinoma, PAAD Pancreatic adenocarcinoma, PCPG Pheochromocytoma and paraganglioma, PRAD Prostate adenocarcinoma, READ Rectum adenocarcinoma, SARC Sarcoma, SKCM Skin cutaneous melanoma, STAD Stomach adenocarcinoma, TGCT Testicular germ cell tumors, THCA Thyroid carcinoma, THYM Thymoma, UCEC Uterine corpus endometrial carcinoma, UCS Uterine carcinosarcoma, UVM Uveal melanoma. **E**. Kaplan-Meier plots for the TCGA-LUAD and GDC-TCGA LAML cohorts separated according to the quartile high or low expression of *SLC7A11*. **F**. Quantification of western blots presented in Fig. 2. One-way ANOVA corrected with a Dunnett's test for multiple comparisons; n=3; mean±SEM; **p=0.0012; ***p=0.0001.

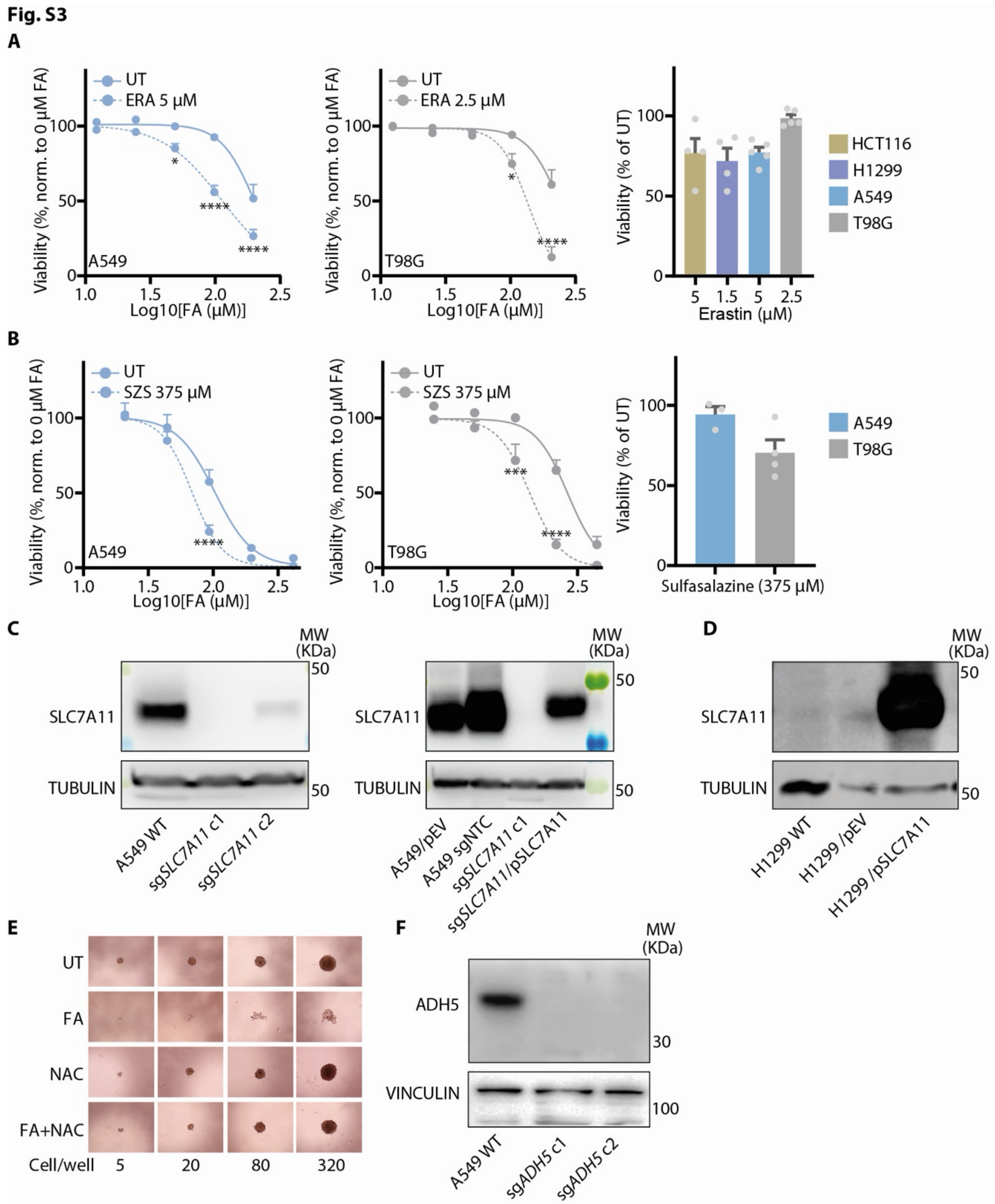

Fig. S3. SLC7A11 perturbation and formaldehyde tolerance

A. Formaldehyde (FA) tolerance of A549 and T98G cells in the presence of erastin (ERA) (mean±SEM, n=5 and two-way ANOVA; for A549: *p=0.0345 and ****p<0.0001; for T98G: *p=0.0175 and ****p<0.0001). In the right bar plot the viability at 0 μM FA for a given ERA concentration is shown. **B.** FA tolerance of A549 and T98G cells in the presence of sulfasalazine (SZS) (mean±SEM, n=3 and two-way ANOVA; for A549: ****p<0.0001; for T98G: ***p=0.0003 and ****p<0.0001). In the right bar plot the viability at 0 μM FA for a given SZS concentration is shown. **C.** Left: western blot showing inactivation of SLC7A11 in A549 cells (sg*SLC7A11* represents cells infected with a lentiviral vector expressing a sgRNA against *SLC7A11*; WT: wild-type). Right: Complementation of *SLC7A11*-deficient cells with a

lentiviral vector expressing SLC7A11 (pSLC7A11). **D.** Western blot showing overexpression of SLC7A11 (pSLC7A11) in H1299 cells and their isogenic control harboring the empty vector (pEV). **E.** Representative images of 3D sphere formation assay using A549 cells in the presence of 200 μM FA and/or N-acetylcysteine (NAC, 500 μM). **F.** Western blot showing the inactivation of ADH5 in A549 cells.

Fig. S4

A

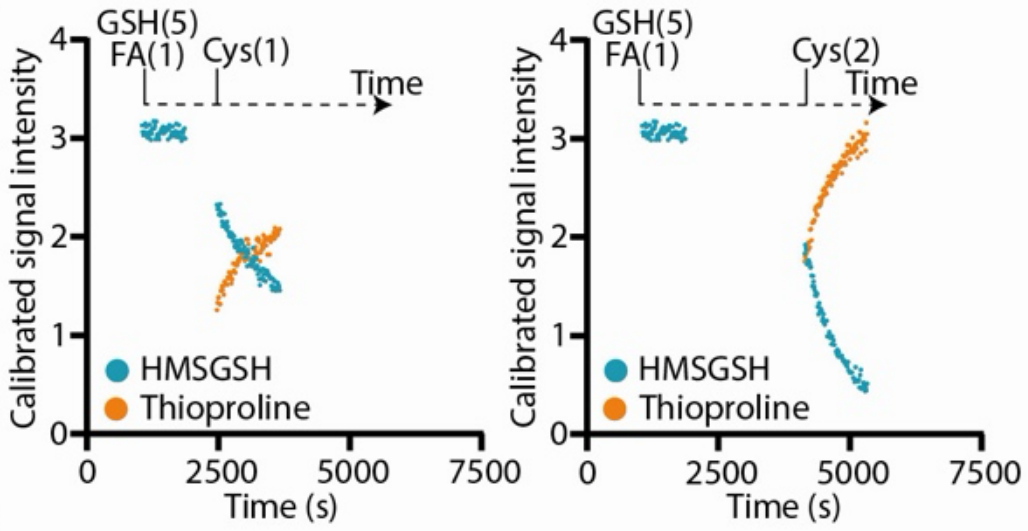
GSH(5)
FA(1)
Cys(1)
Time
GSH(5)
FA(1)
Cys(2)
Time
Calibrated signal intensity
HMSGSH
Thioproline
Time (s)

B

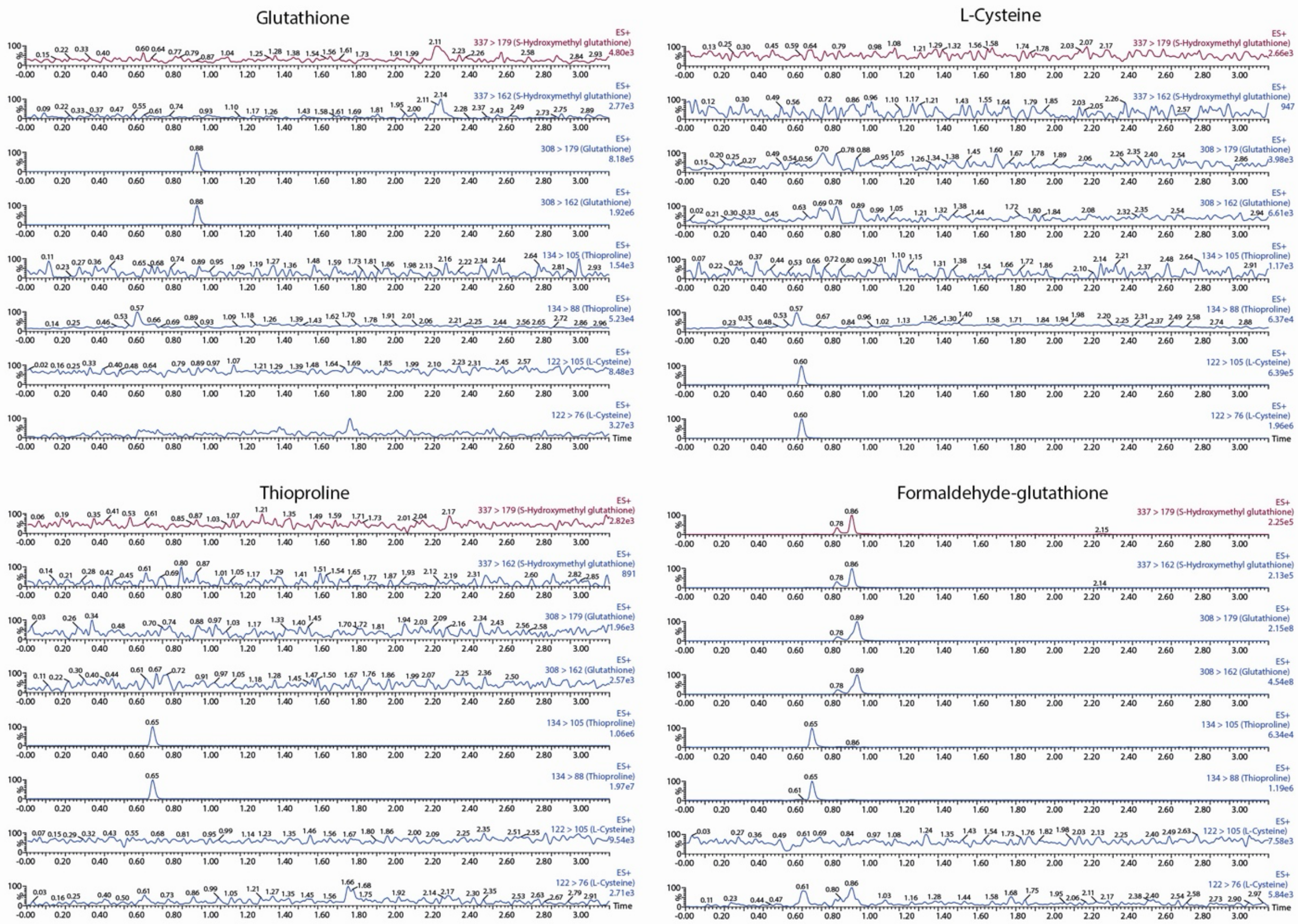
Glutathione
L-Cysteine
Thioproline
Formaldehyde-glutathione
337 > 179 (S-Hydroxymethyl glutathione)
337 > 162 (S-Hydroxymethyl glutathione)
308 > 179 (Glutathione)
308 > 162 (Glutathione)
134 > 105 (Thioproline)
134 > 88 (Thioproline)
122 > 105 (L-Cysteine)
122 > 76 (L-Cysteine)
ES+
Time

C

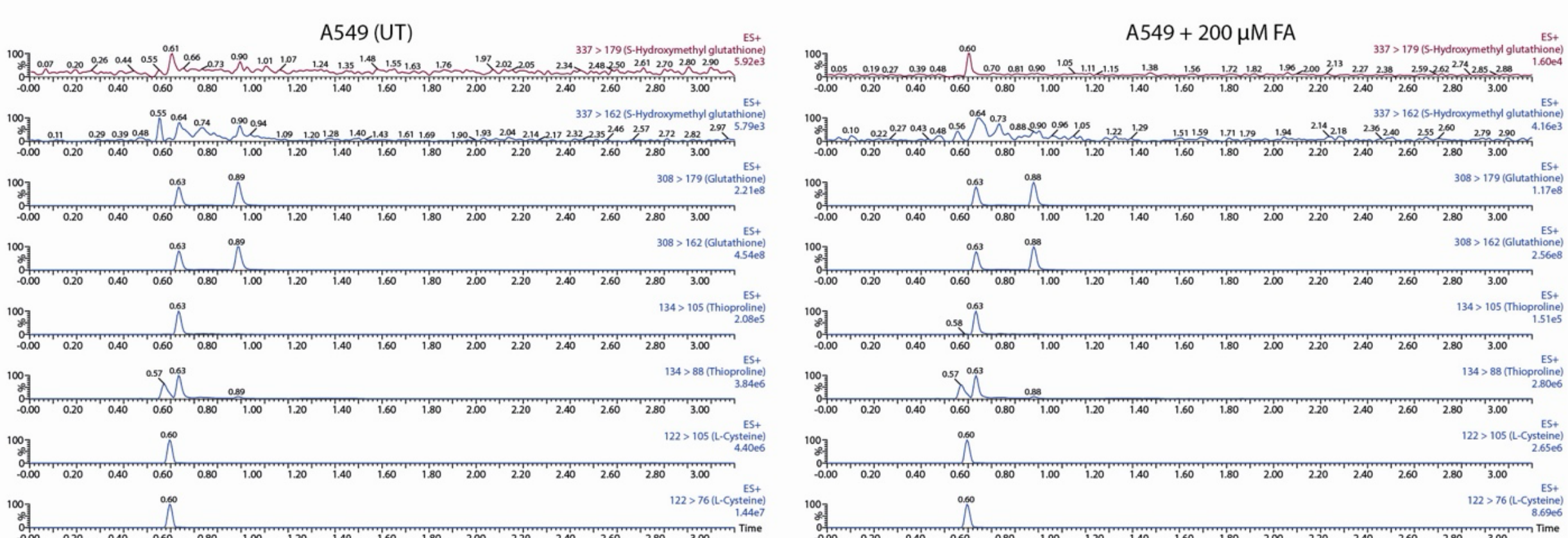
A549 (UT)
A549 + 200 µM FA
337 > 179 (S-Hydroxymethyl glutathione)
337 > 162 (S-Hydroxymethyl glutathione)
308 > 179 (Glutathione)
308 > 162 (Glutathione)
134 > 105 (Thioproline)
134 > 88 (Thioproline)
122 > 105 (L-Cysteine)
122 > 76 (L-Cysteine)
ES+
Time

Fig. S4. Formaldehyde reacts with cysteine. A. Time-dependent formation of thioproline and S-hydroxymethyl-glutathione (HMSGSH) monitored by nuclear magnetic resonance (NMR) after addition of 1 mM cysteine at 1875 seconds (left panel), or 2 mM cysteine at 3632 seconds (right panel). **B.** LC-ESI-MS/MS chromatograms obtained for chemical standards corresponding to glutathione (top left); L-cysteine (top right); thioproline (bottom left); and the product of the reaction between formaldehyde (FA) and GSH corresponding to HMSGSH (bottom right). **C.** LC-ESI-MS/MS chromatograms obtained from representative A549 cells untreated (UT) (left panel) or exposed to 200 µM FA (right panel) for 24 h. The SRM channels corresponding to GSH, cysteine, thioproline, and HMSGSH are shown. Metabolite quantification was based on chromatographic peak areas, from which concentrations were determined and normalized to the number of cells collected in each sample.

Fig. S5

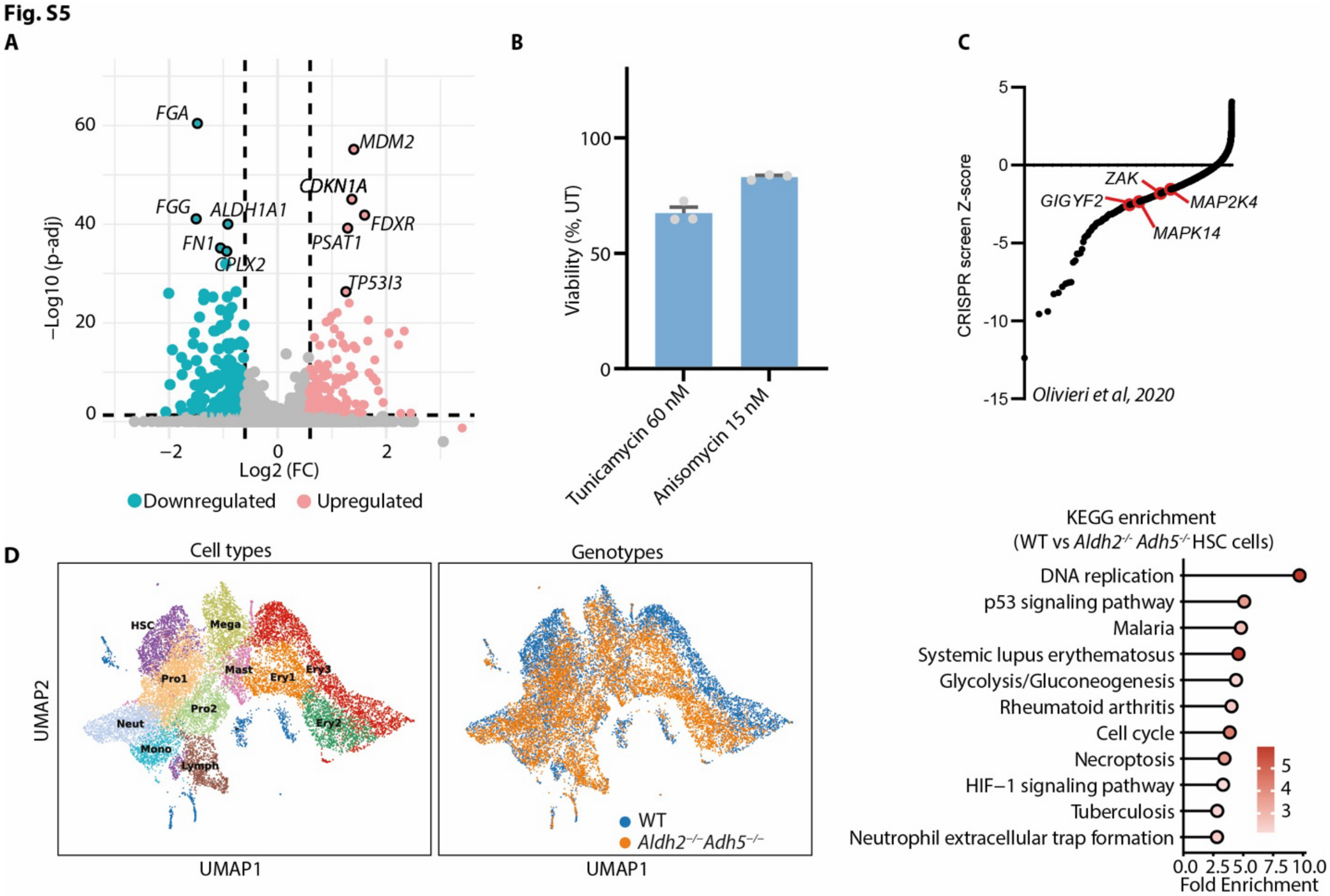

Fig. S5. The cellular response to formaldehyde. A. Volcano plot showing significantly dysregulated genes when comparing A549 cells treated with DMSO and A549 cells treated with 5 µM erastin (ERA) for 48 h, adding 200 µM formaldehyde (FA) for the last 24 h (Fold change > 1.5; adj-P ≤0.05). **B.** Viability at 0 µM FA for a given concentration of anisomycin or sulfasalazine (SZS) (relative to Figs. 4E and 4F). **C**. CRISPR screen data performed in retinal pigment epithelial (RPE) cells exposed to 63.5 µM FA over 10 doubling times, highlighting the negative scores of genes involved in the ribotoxic stress response (RSR) *(45)*. **D.** Uniform Manifold Approximation and Projection (UMAP) depicting single-cell architecture of mouse bone marrow cells. The middle panels highlight the hematopoietic stem cells (HSC) by genotype. Right: KEGG analysis of differentially regulated genes in HSC comparing *Aldh2*$^{-/-}$*Adh5*$^{-/-}$ with wild-type mice. Data obtained from GSE157832.

Fig. S6

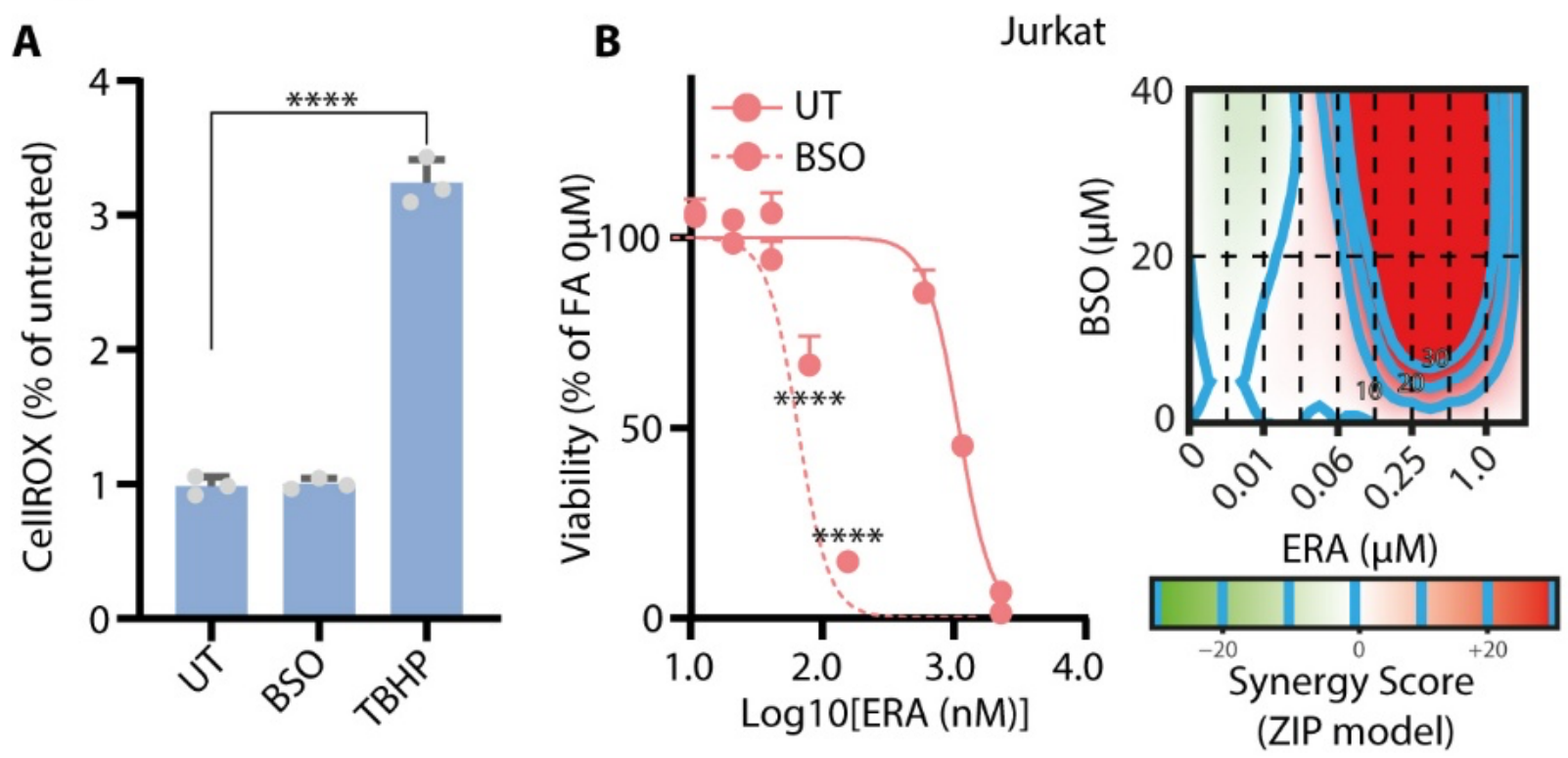

C

Cell line	Expected (Bliss)	Observed (Bliss)	ΔBliss
HCT116	0.287	0.118	+0.169
H1299	0.174	0.078	+0.096
A549	0.669	0.355	+0.314
T98G	0.335	0.095	+0.240

Fig. S6. Pharmacological inhibition of GSH synthesis and SLC7A11 is lethal across several cell lines. **A**. CellROX Deep Red staining in A549 cells exposed to 100 µM L-buthionine sulfoximine (BSO) for 48 h or 500 µM tert-butyl-hydroperoxide (TBHP) for 20 minutes. CellROX signal was quantified in the allophycocyanin (APC) channel and represented relative to the untreated samples (UT) [mean±SEM, n=3, one-way ANOVA analysis (Tukey test for multiple comparisons); ****p<0.0001]. **B.** Viability plot for Jurkat cells exposed to 40 µM BSO and to the indicated concentrations of erastin (ERA) [mean±SEM, two-way ANOVA analysis (Tukey test between UT and BSO 40 µM at each ERA concentration); ****p<0.0001]. The right plot shows the zero-interaction potency (ZIP) synergistic effect of the dual combination. **C.** Assessment of synergism in the data in Fig. 6A by calculating the expected and observed Bliss coefficients and the difference between them.

<u>**Supplemental material titles:**</u>

1. Table S1: List of primers used in the study.
2. Table S2: List of genes dysregulated in A549 cells exposed to formaldehyde and erastin filtered by *p*-adj < 0.01.
3. Uncropped western blot images

NRF2 coordinates cystine importe and glutathione use to sustain ferroptosis resistance and formaldehyde tolerance in cancer

UNCROPPED
WESTERN BLOTS
IMAGES

Fig1 – G (Part 1)

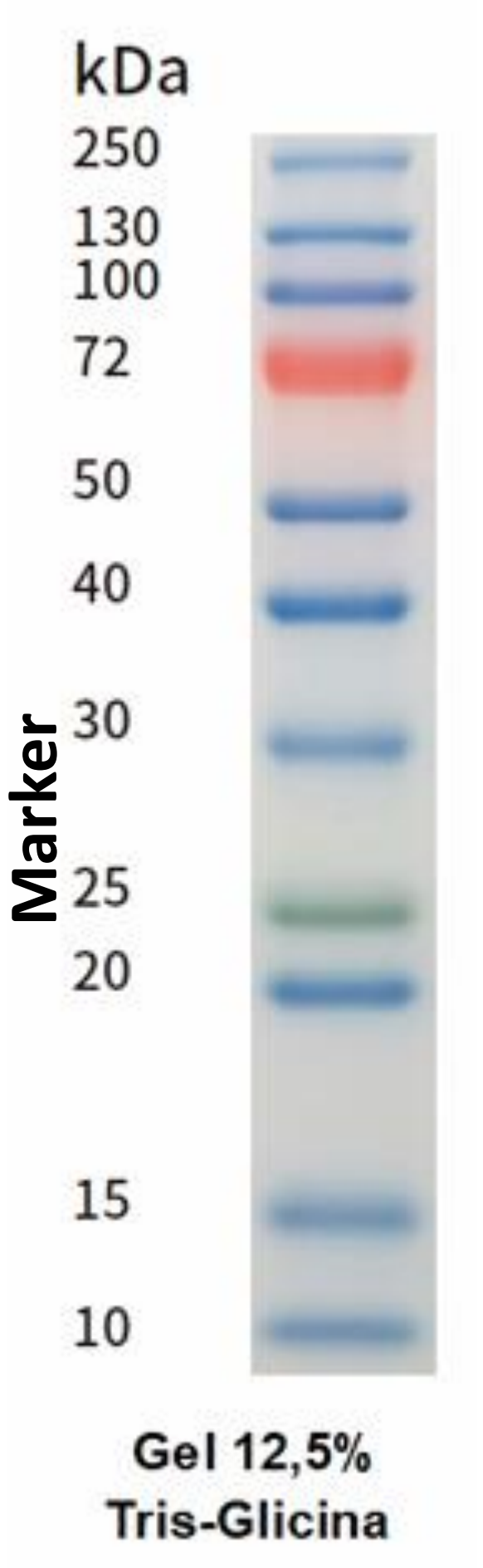
kDa
250
130
100
72
50
40
30
25
20
15
10
Marker
Gel 12,5%
Tris-Glicina

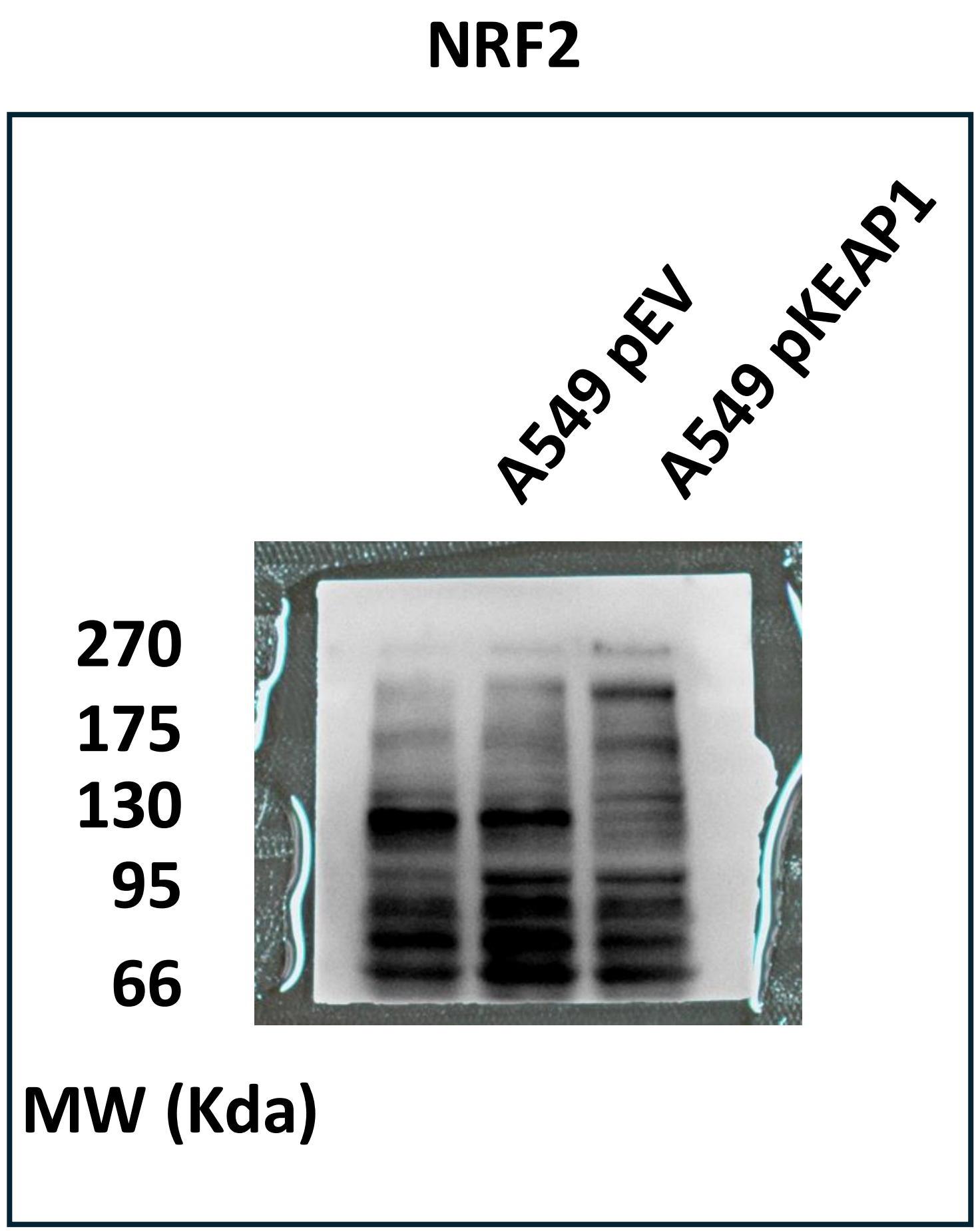
NRF2
A549 pEV
A549 pKEAP1
270
175
130
95
66
MW (Kda)

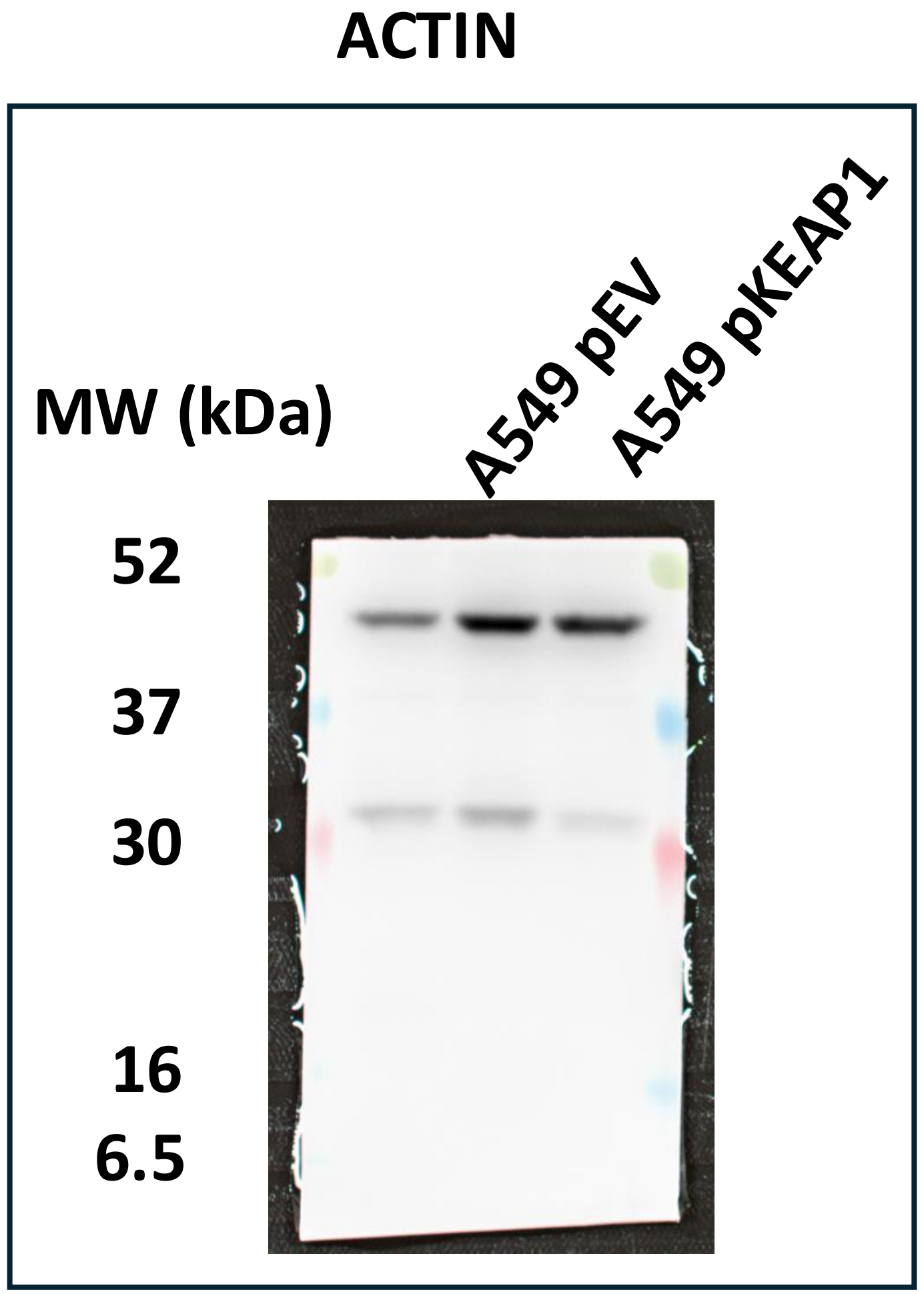
ACTIN
MW (kDa)
A549 pEV
A549 pKEAP1
52
37
30
16
6.5

Fig1 – G (Part 2)

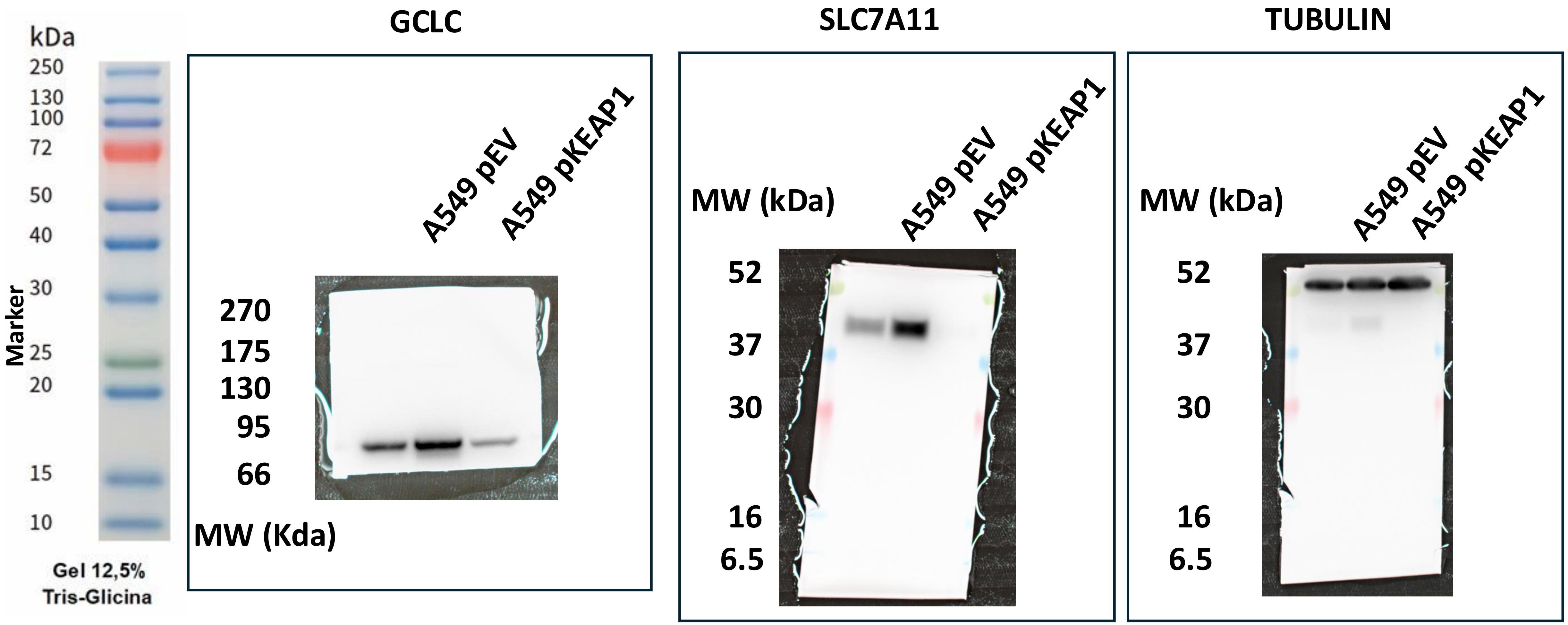

kDa
250
130
100
72
50
40
30
25
20
15
10
Marker
Gel 12,5%
Tris-Glicina
GCLC
A549 pEV
A549 pKEAP1
270
175
130
95
66
MW (Kda)
SLC7A11
MW (kDa)
A549 pEV
A549 pKEAP1
52
37
30
16
6.5
TUBULIN
MW (kDa)
A549 pEV
A549 pKEAP1
52
37
30
16
6.5

Fig1 – G (Part 3)

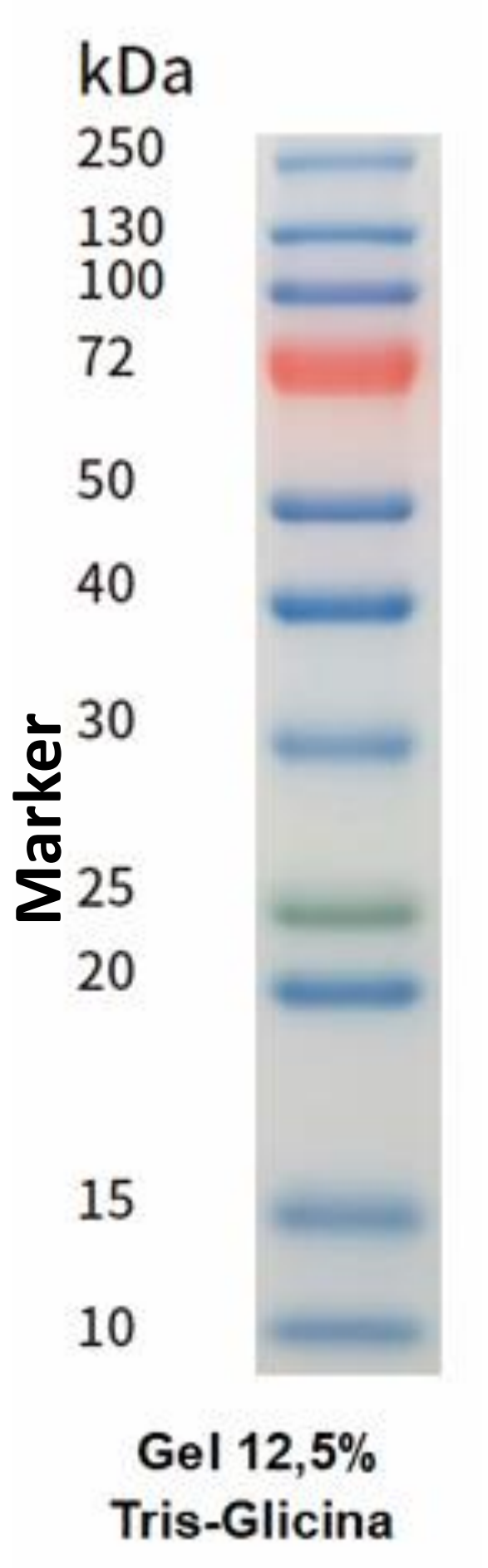

kDa
250
130
100
72
50
40
30
25
20
15
10
Marker
Gel 12,5%
Tris-Glicina

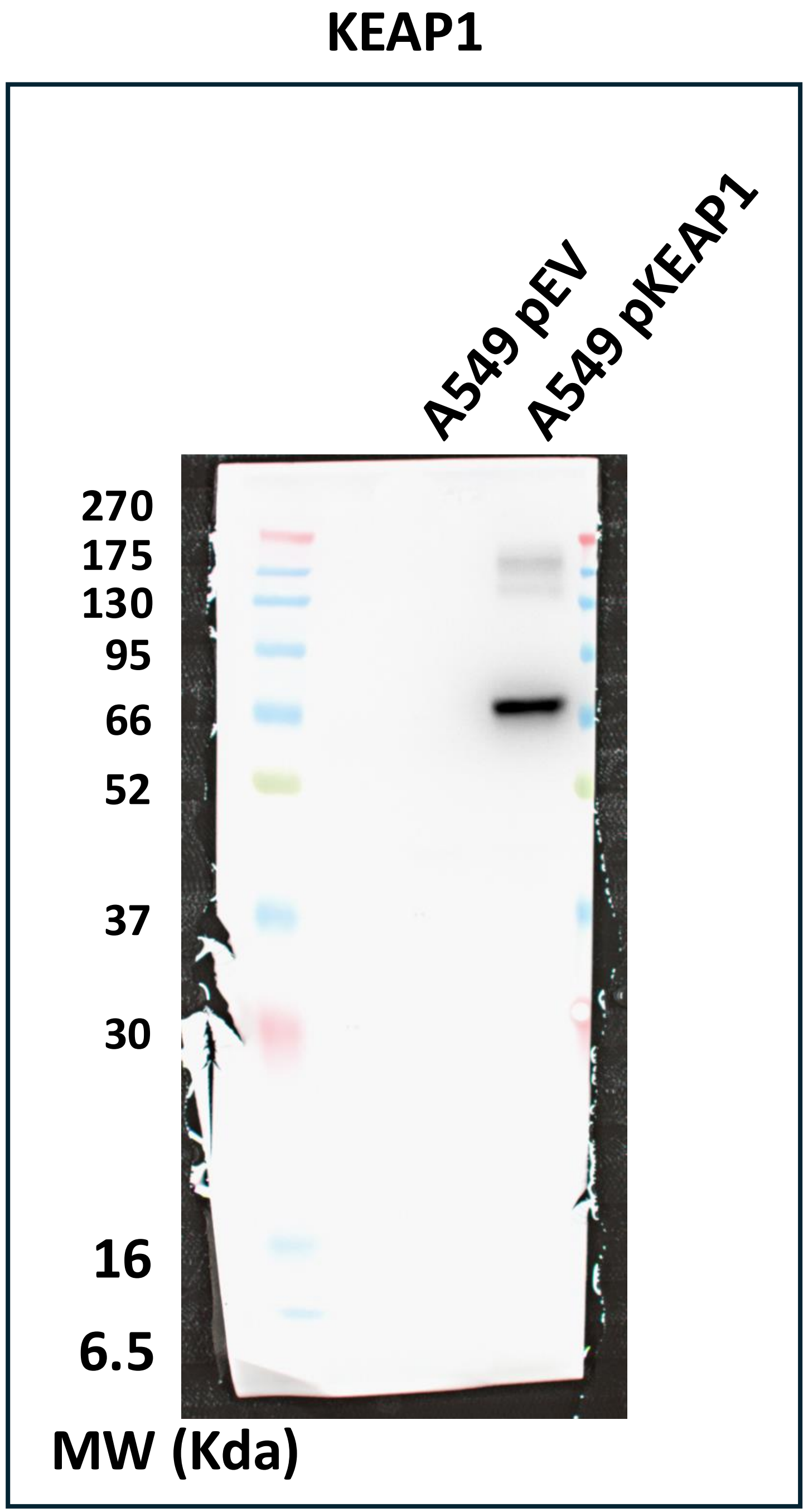

KEAP1
A549 pEV
A549 pKEAP1
270
175
130
95
66
52
37
30
16
6.5
MW (Kda)

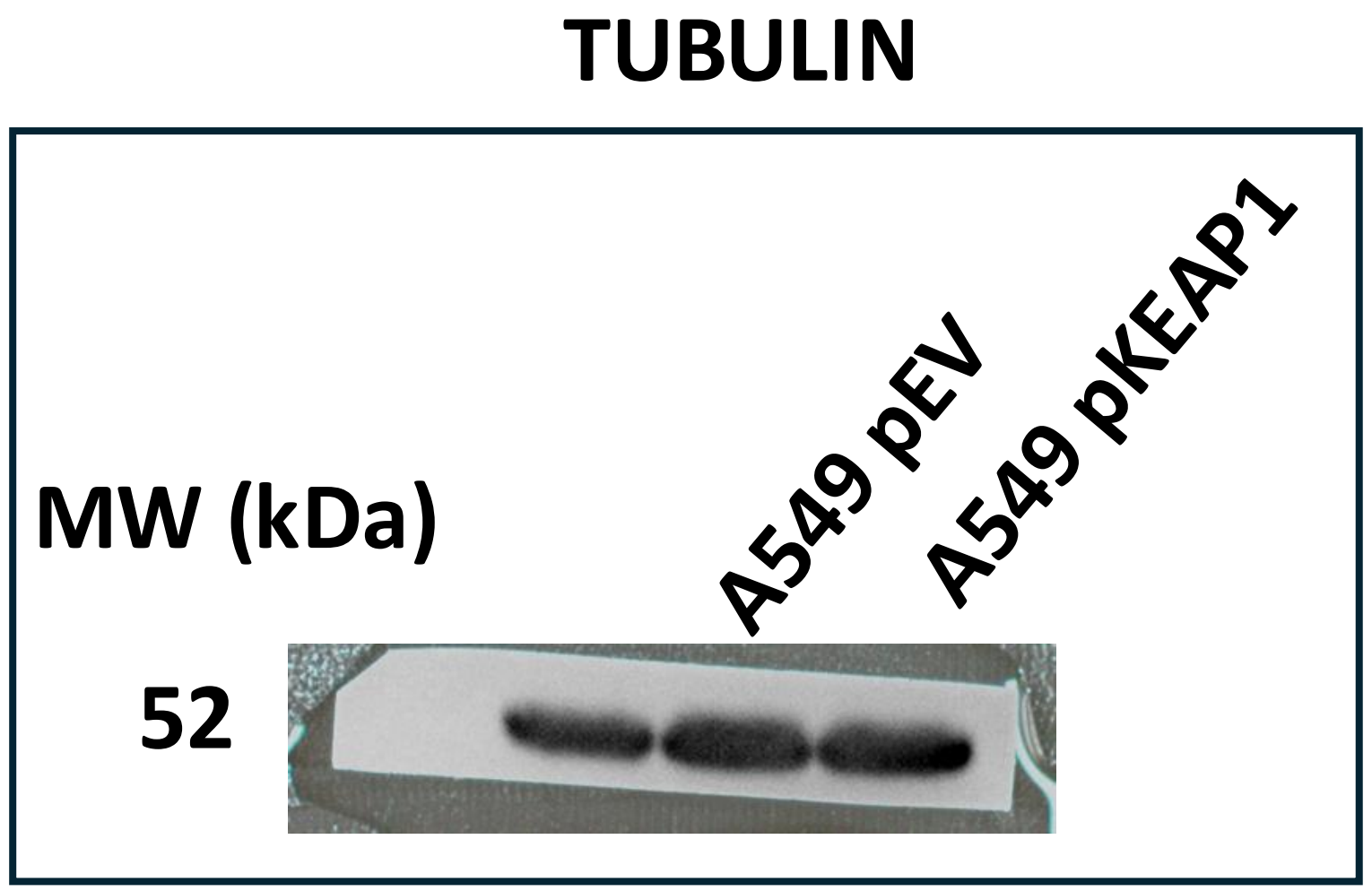

TUBULIN
MW (kDa)
A549 pEV
A549 pKEAP1
52

Fig2 – F (Part 1)

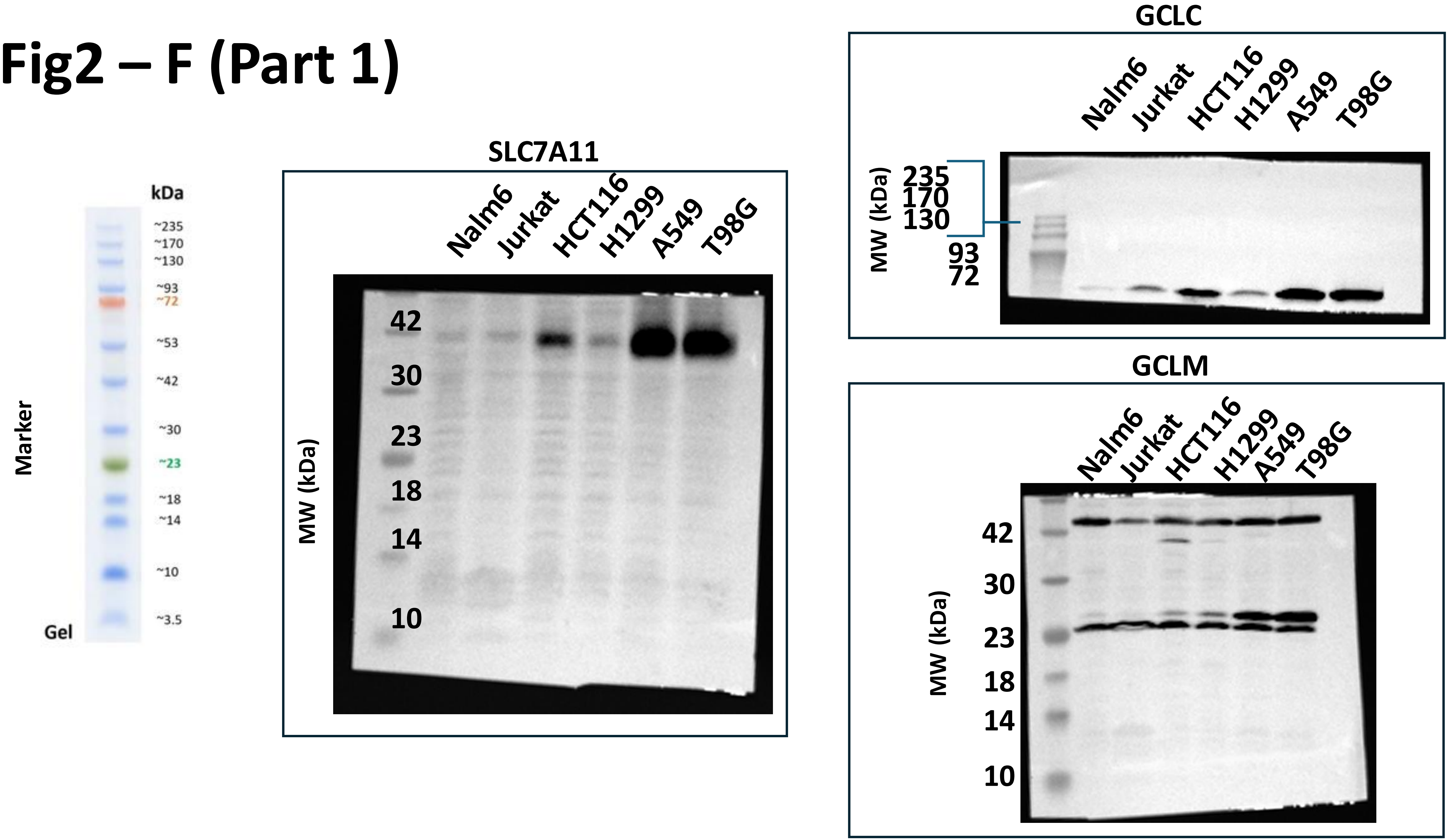

Marker
kDa
~235
~170
~130
~93
~72
~53
~42
~30
~23
~18
~14
~10
~3.5
Gel
SLC7A11
Nalm6
Jurkat
HCT116
H1299
A549
T98G
MW (kDa)
42
30
23
18
14
10
GCLC
Nalm6
Jurkat
HCT116
H1299
A549
T98G
MW (kDa)
235
170
130
93
72
GCLM
Nalm6
Jurkat
HCT116
H1299
A549
T98G
MW (kDa)
42
30
23
18
14
10

Fig2 – F (Part 2)

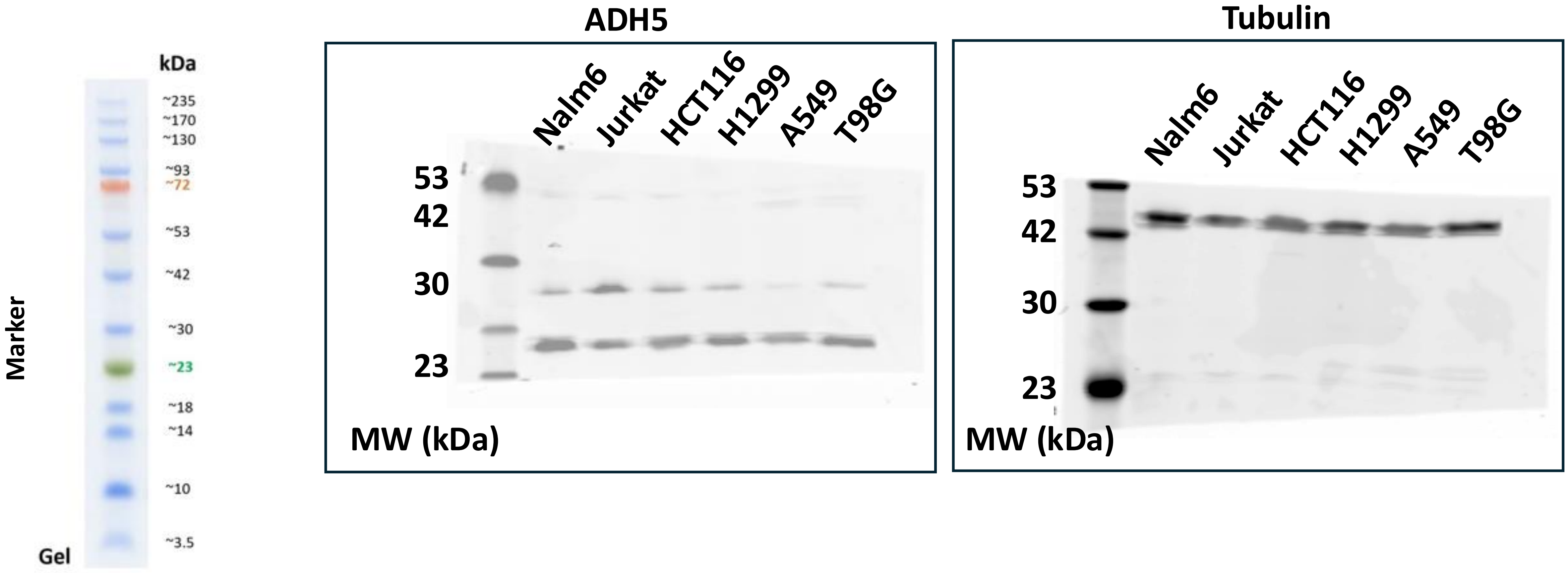
Marker
Gel
kDa
~235
~170
~130
~93
~72
~53
~42
~30
~23
~18
~14
~10
~3.5
ADH5
Nalm6
Jurkat
HCT116
H1299
A549
T98G
53
42
30
23
MW (kDa)
Tubulin
Nalm6
Jurkat
HCT116
H1299
A549
T98G
53
42
30
23
MW (kDa)

Fig4-D

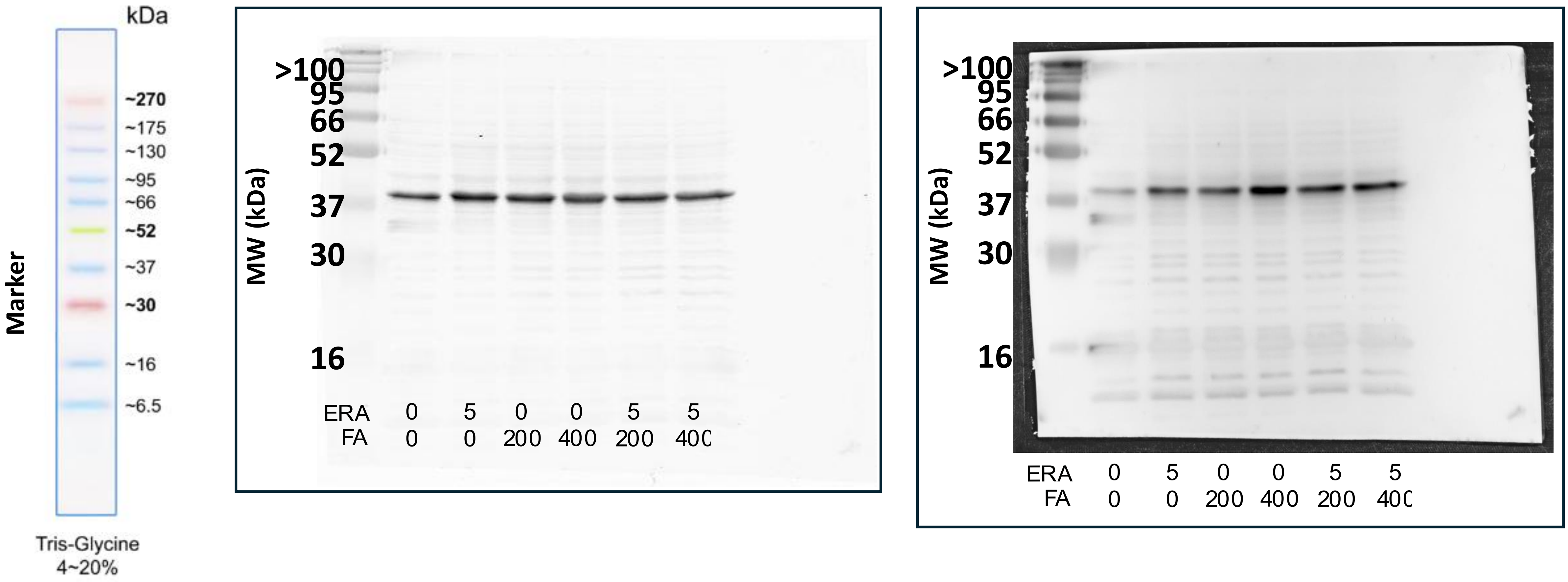

kDa
~270
~175
~130
~95
~66
~52
~37
~30
~16
~6.5
Marker
Tris-Glycine
4~20%
EIF2a
MW (kDa)
>100
95
66
52
37
30
16
ERA 0 5 0 0 5 5
FA 0 0 200 400 200 400
P-EIF2a
MW (kDa)
>100
95
66
52
37
30
16
ERA 0 5 0 0 5 5
FA 0 0 200 400 200 400

Fig4 – G

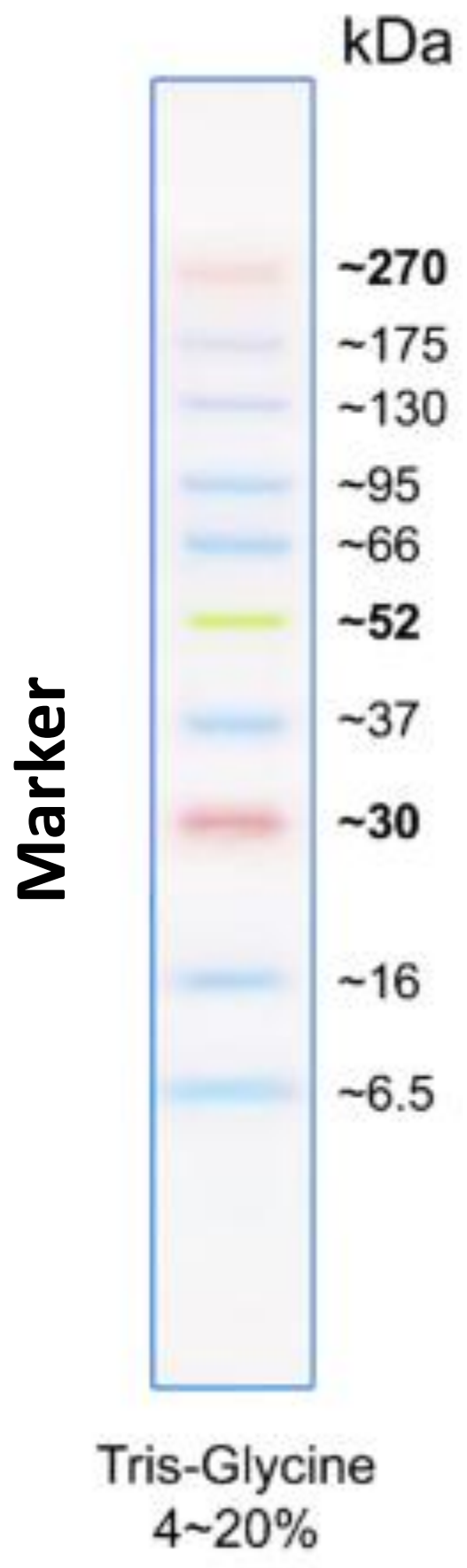

kDa
~270
~175
~130
~95
~66
~52
~37
~30
~16
~6.5
Marker
Tris-Glycine
4~20%

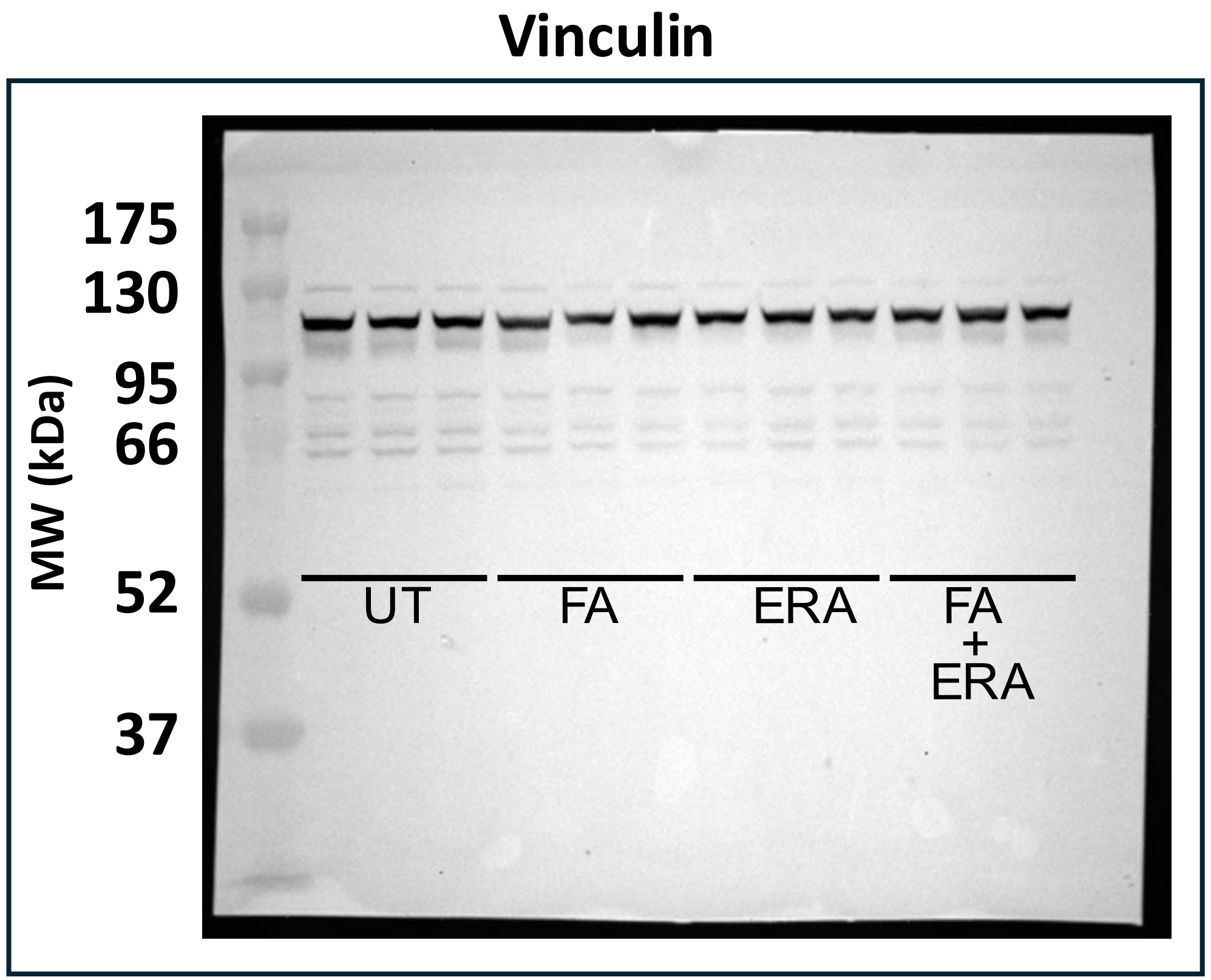

Vinculin
175
130
95
66
52
37
MW (kDa)
UT
FA
ERA
FA
+
ERA

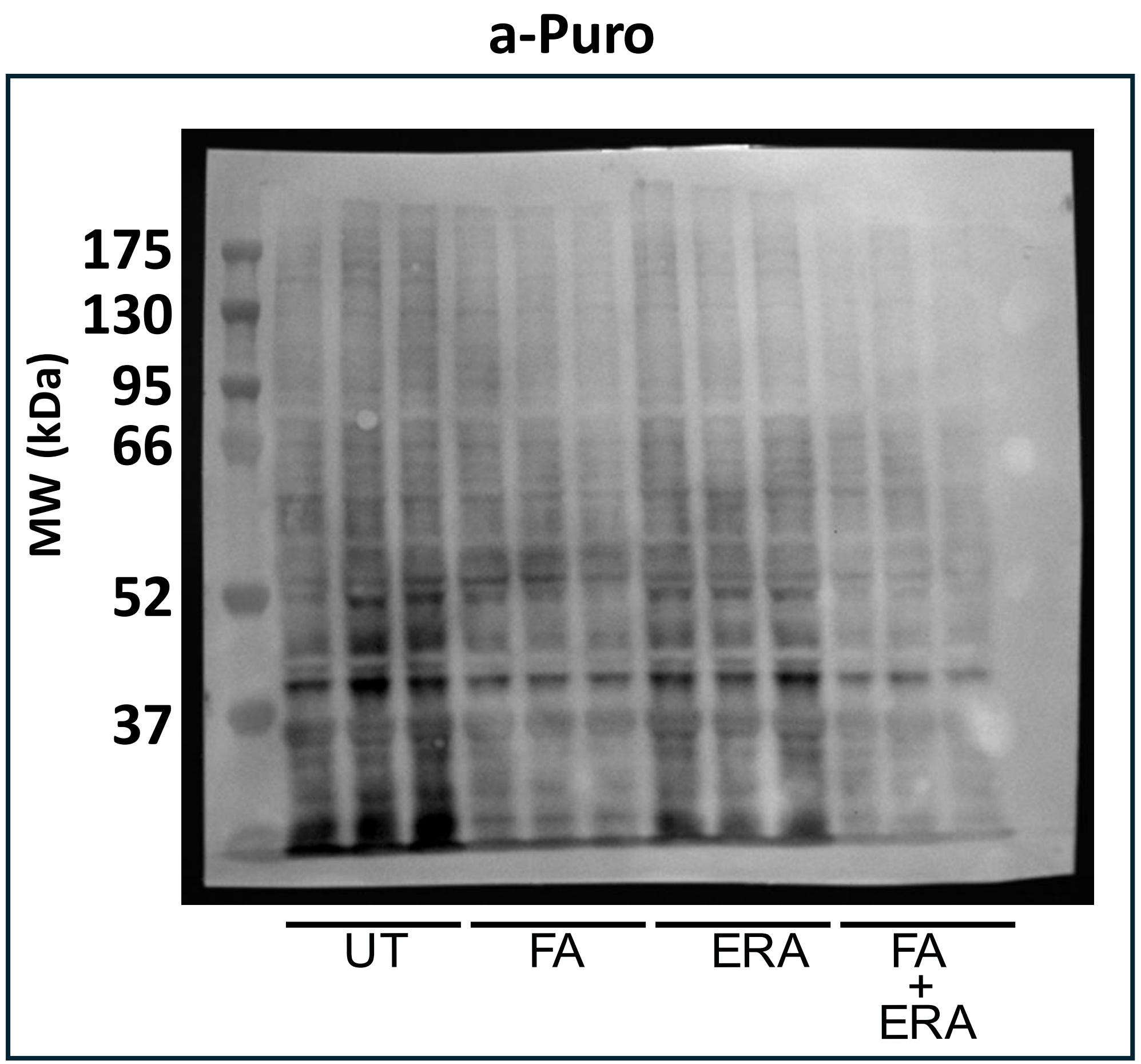

a-Puro
175
130
95
66
52
37
MW (kDa)
UT
FA
ERA
FA
+
ERA

Fig4 – H

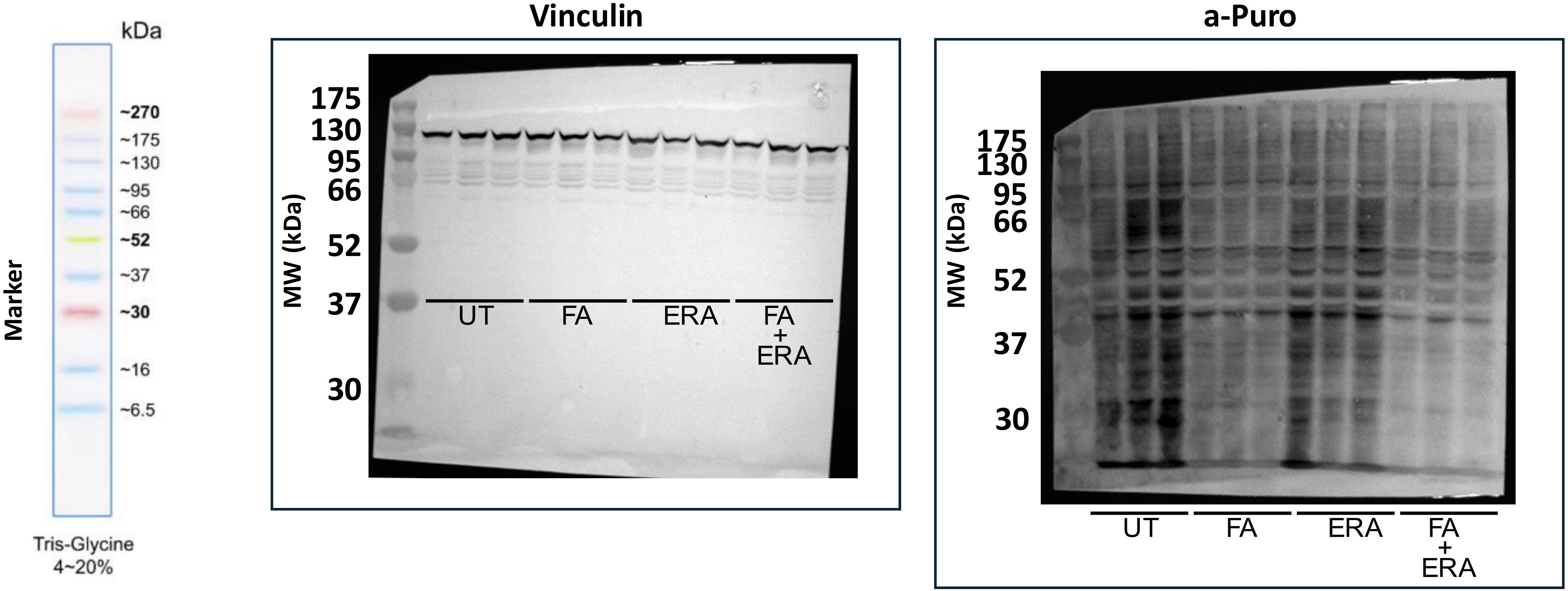

kDa
~270
~175
~130
~95
~66
~52
~37
~30
~16
~6.5
Marker
Tris-Glycine
4~20%
Vinculin
175
130
95
66
52
37
30
MW (kDa)
UT
FA
ERA
FA
+
ERA
a-Puro
175
130
95
66
52
37
30
MW (kDa)
UT
FA
ERA
FA
+
ERA

FigSup1 – D

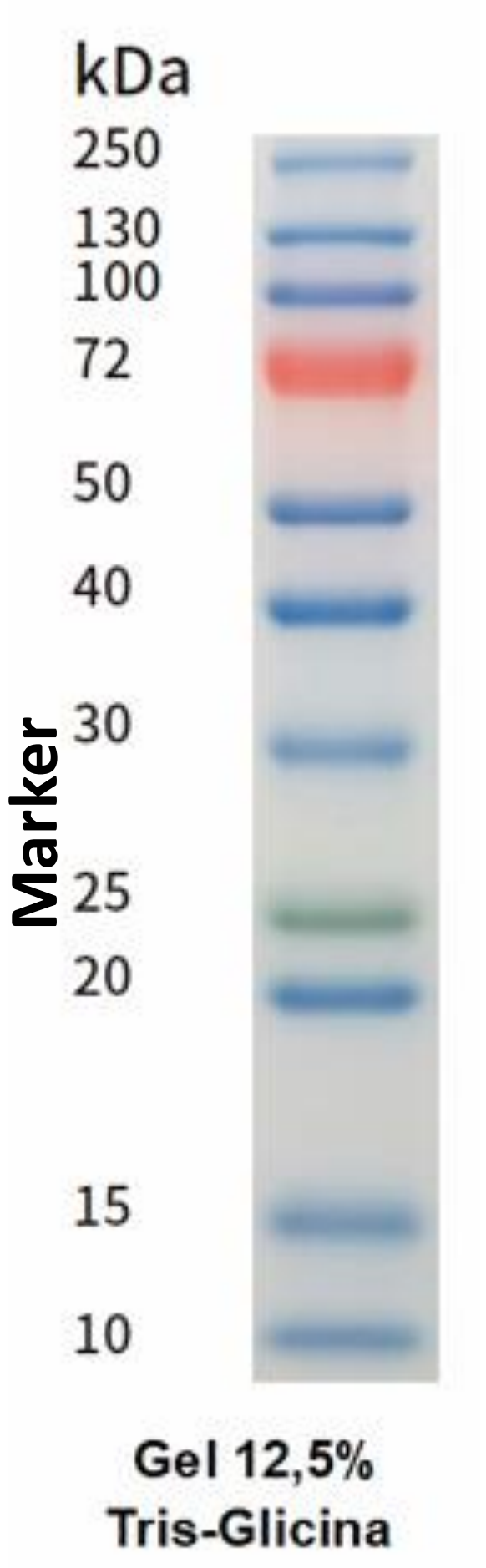

GSS

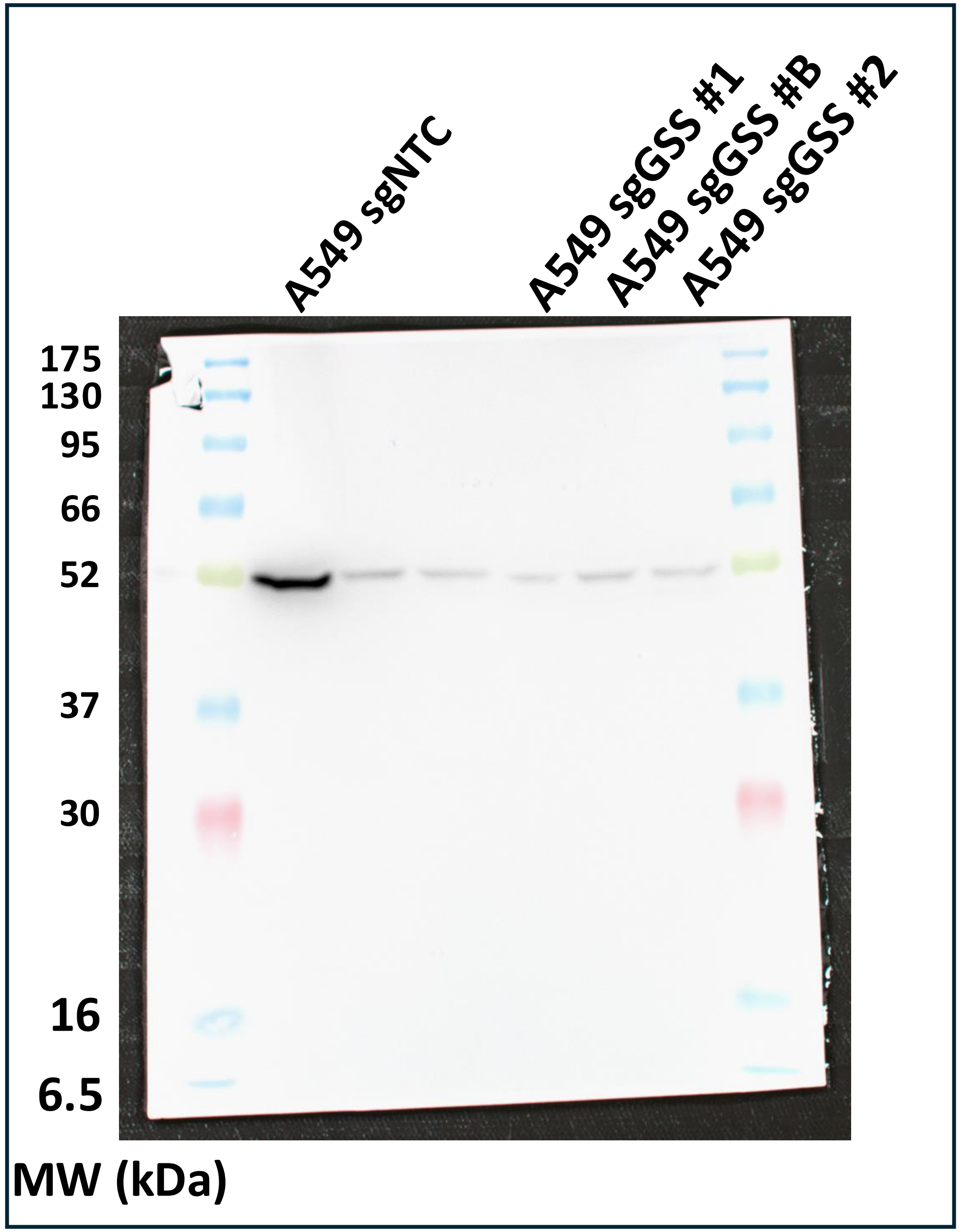

ACTIN

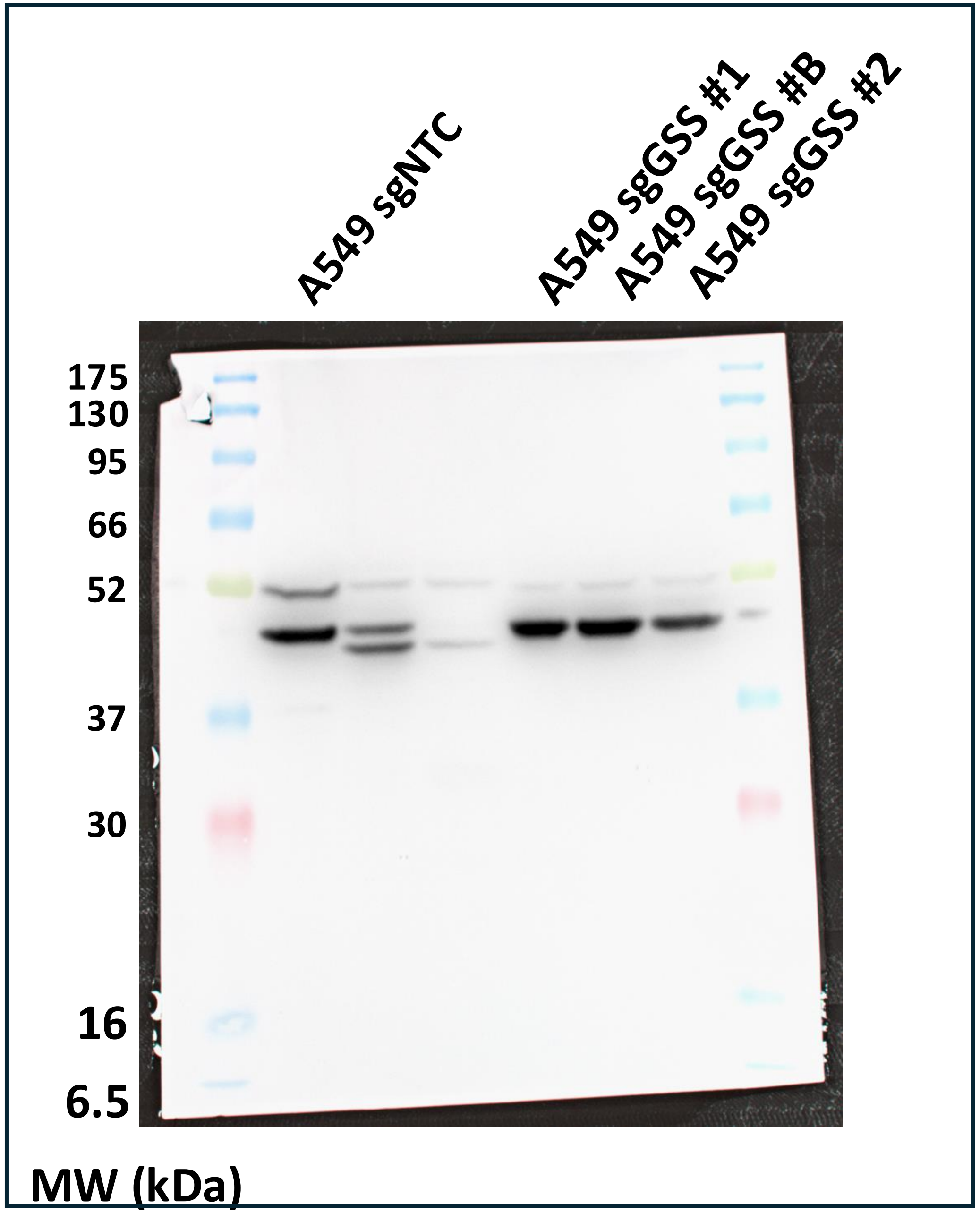

FigSup1 – G (Part 1)

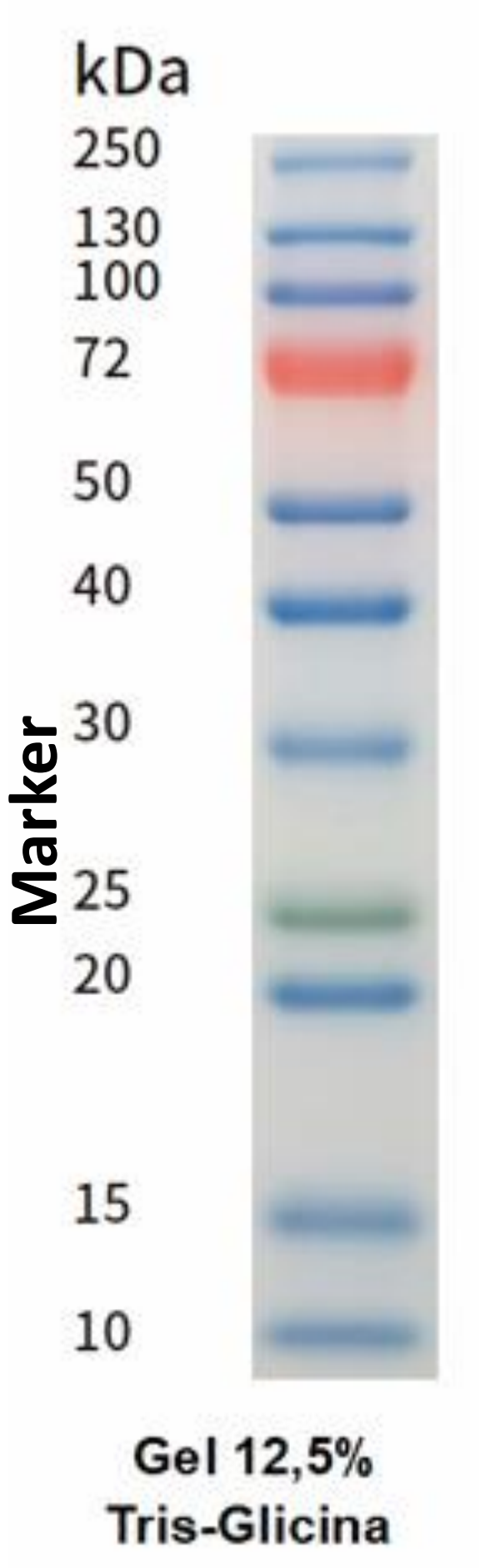

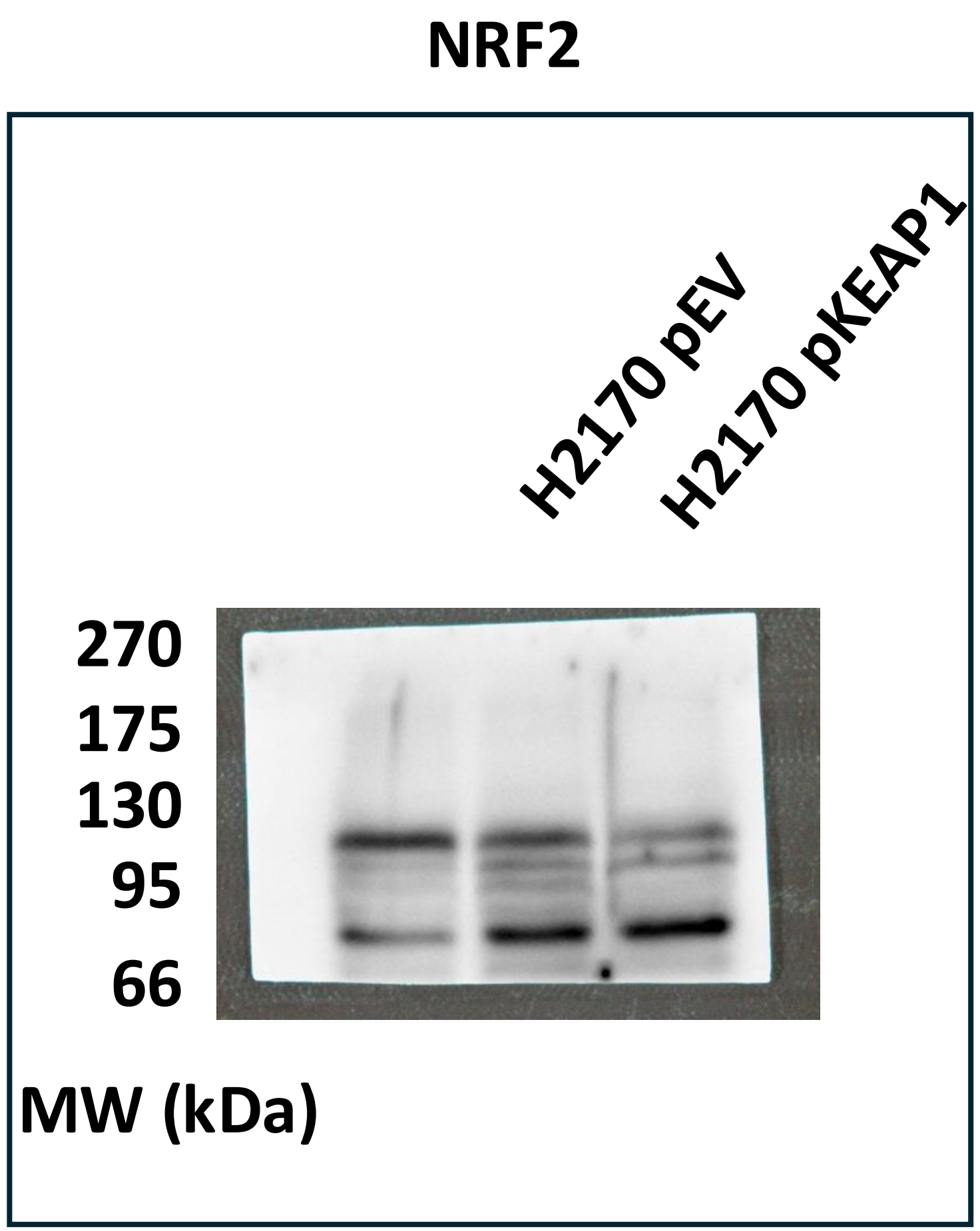

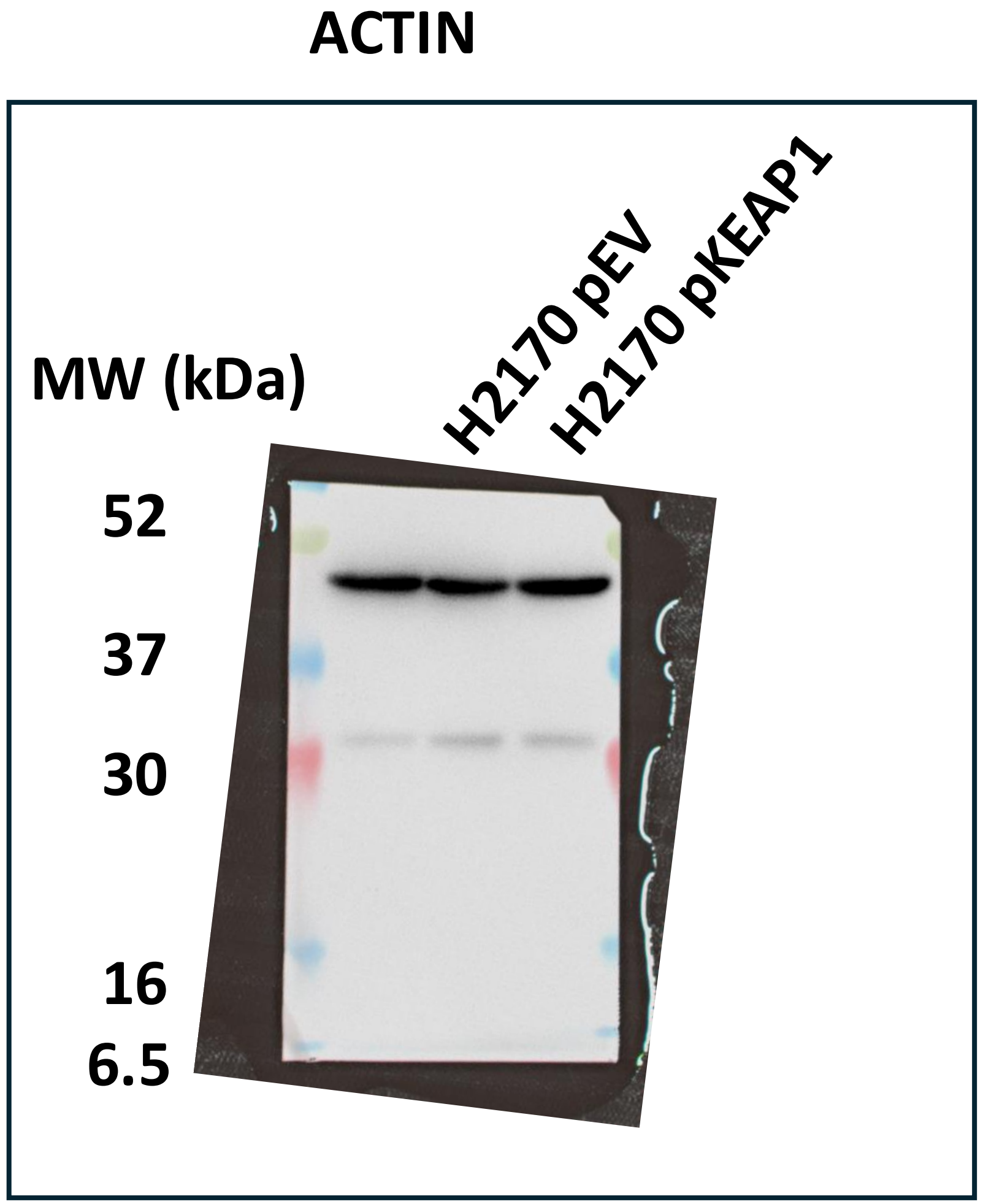

FigSup1 – G (Part 2)

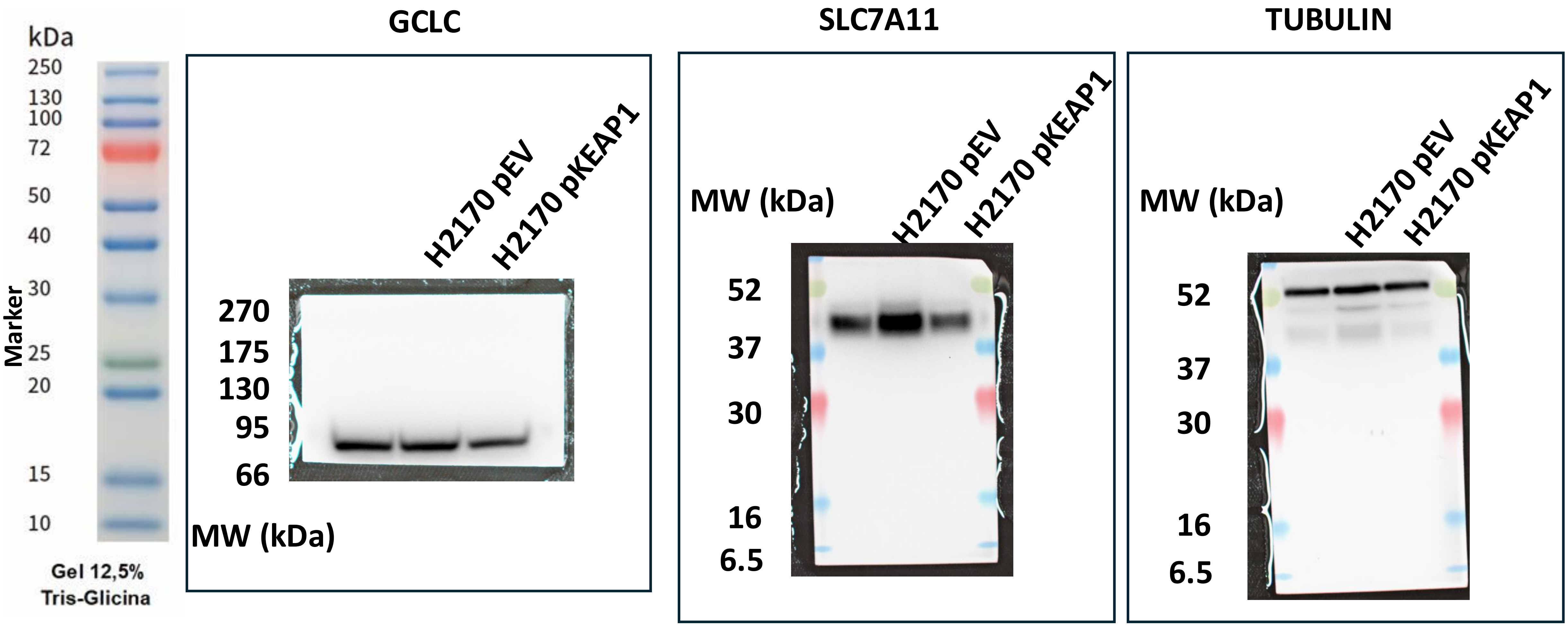

FigSup1 – G (Part 3)

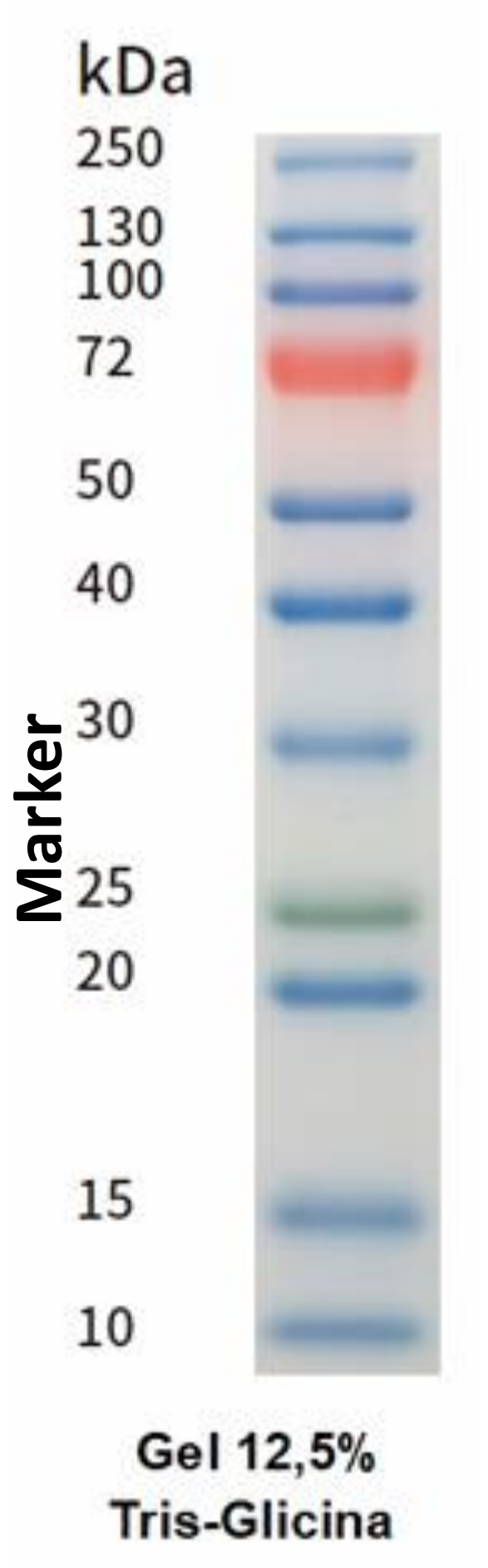

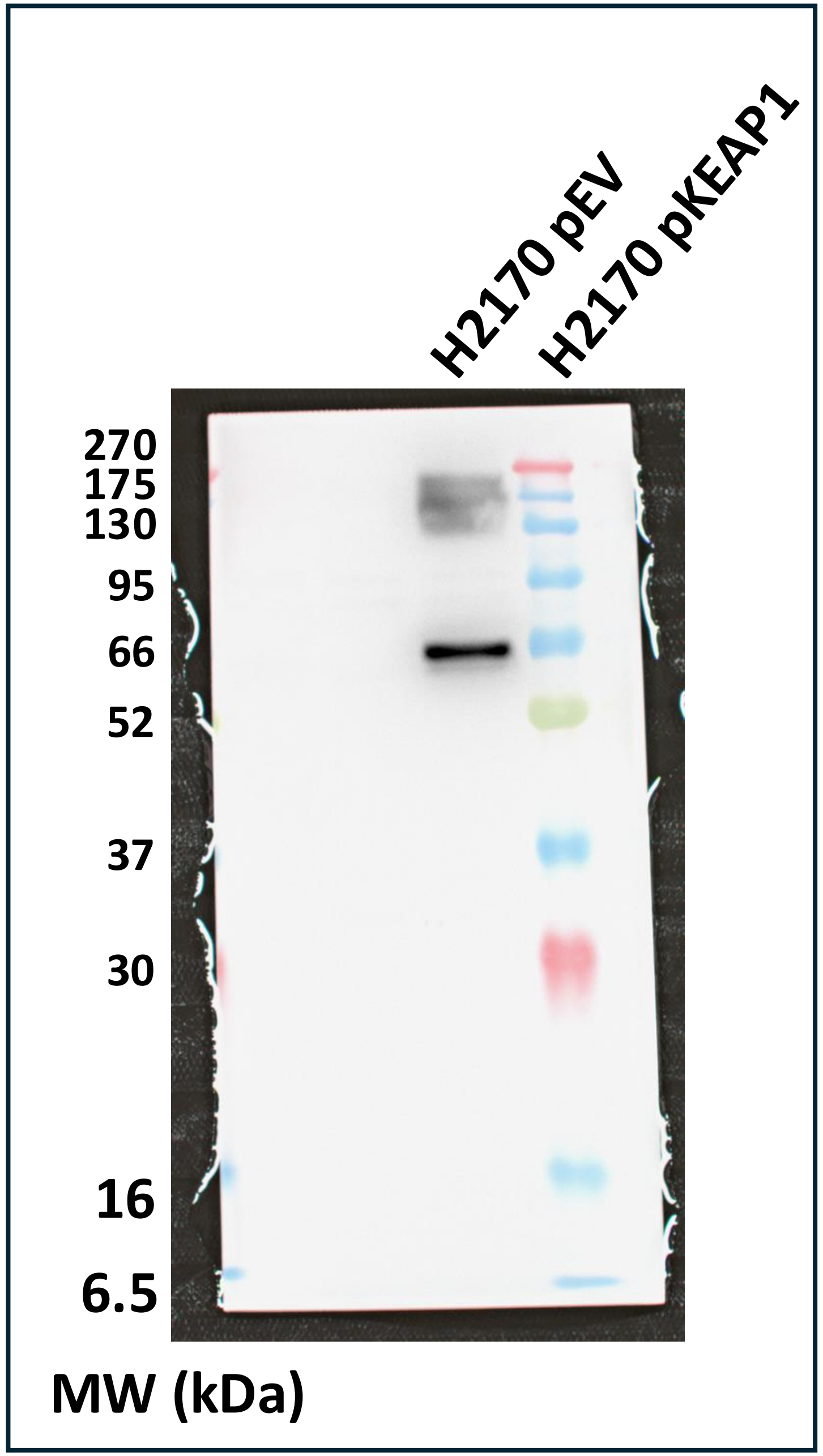

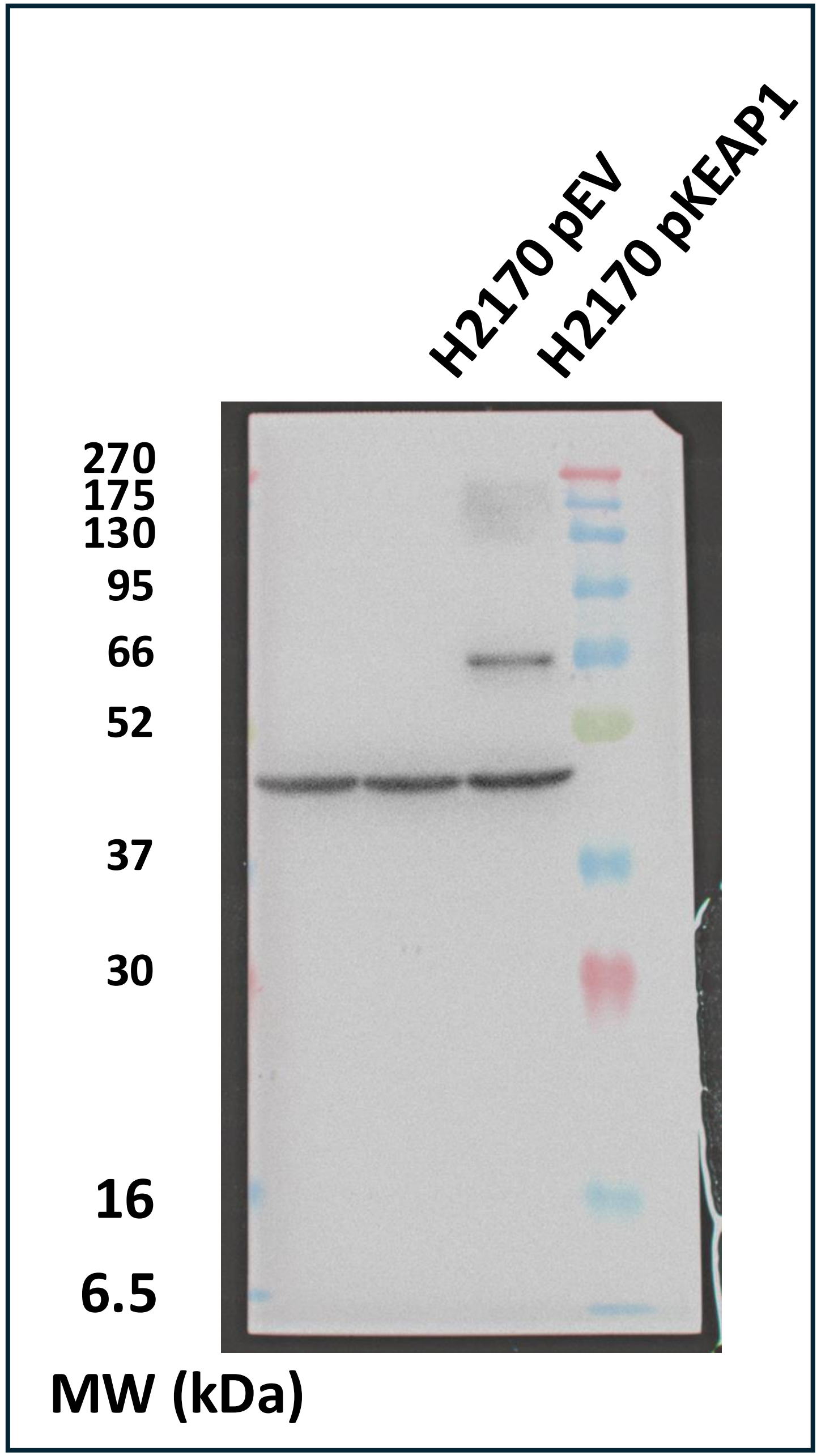

FigSup3 – C (Part 1)

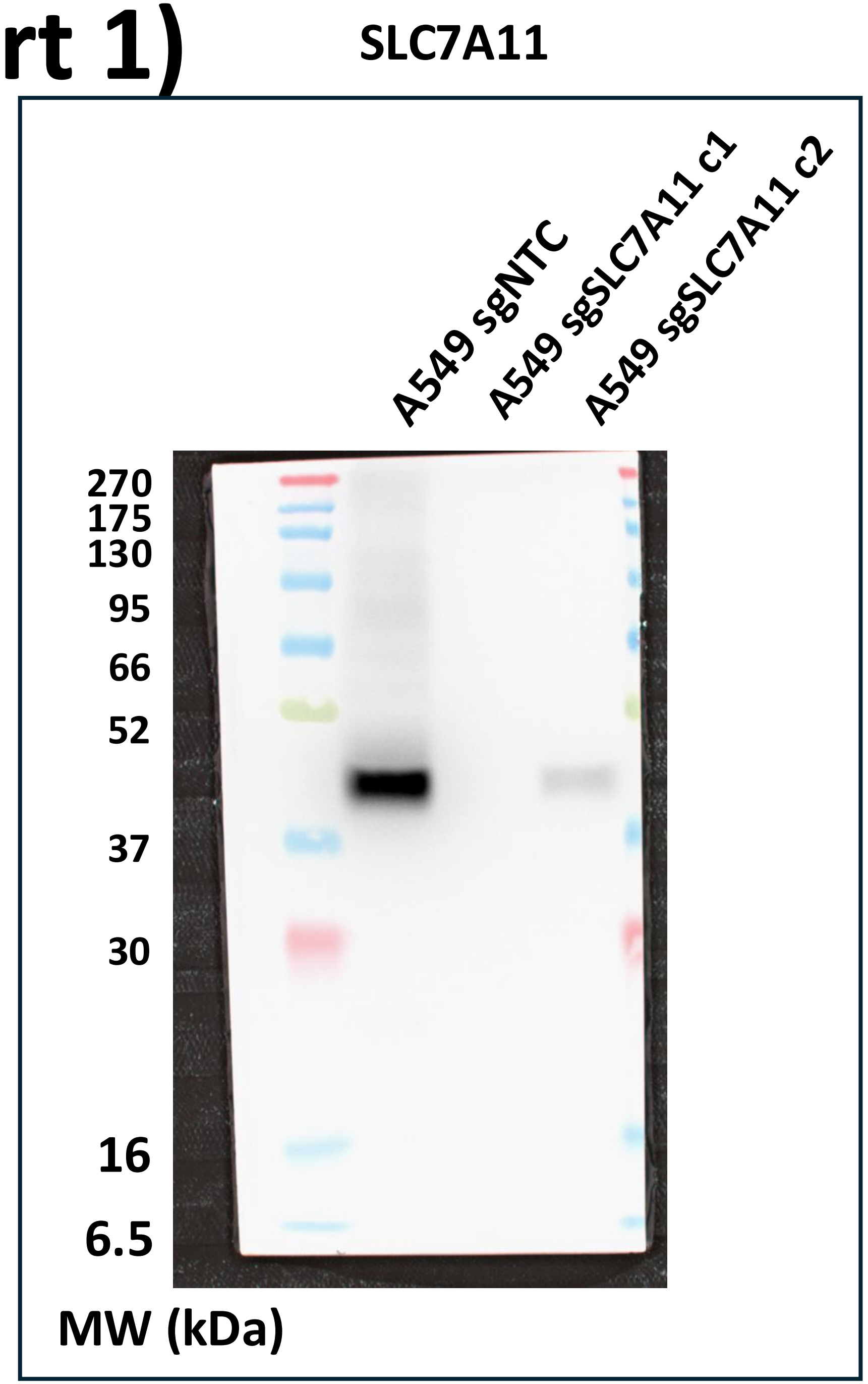

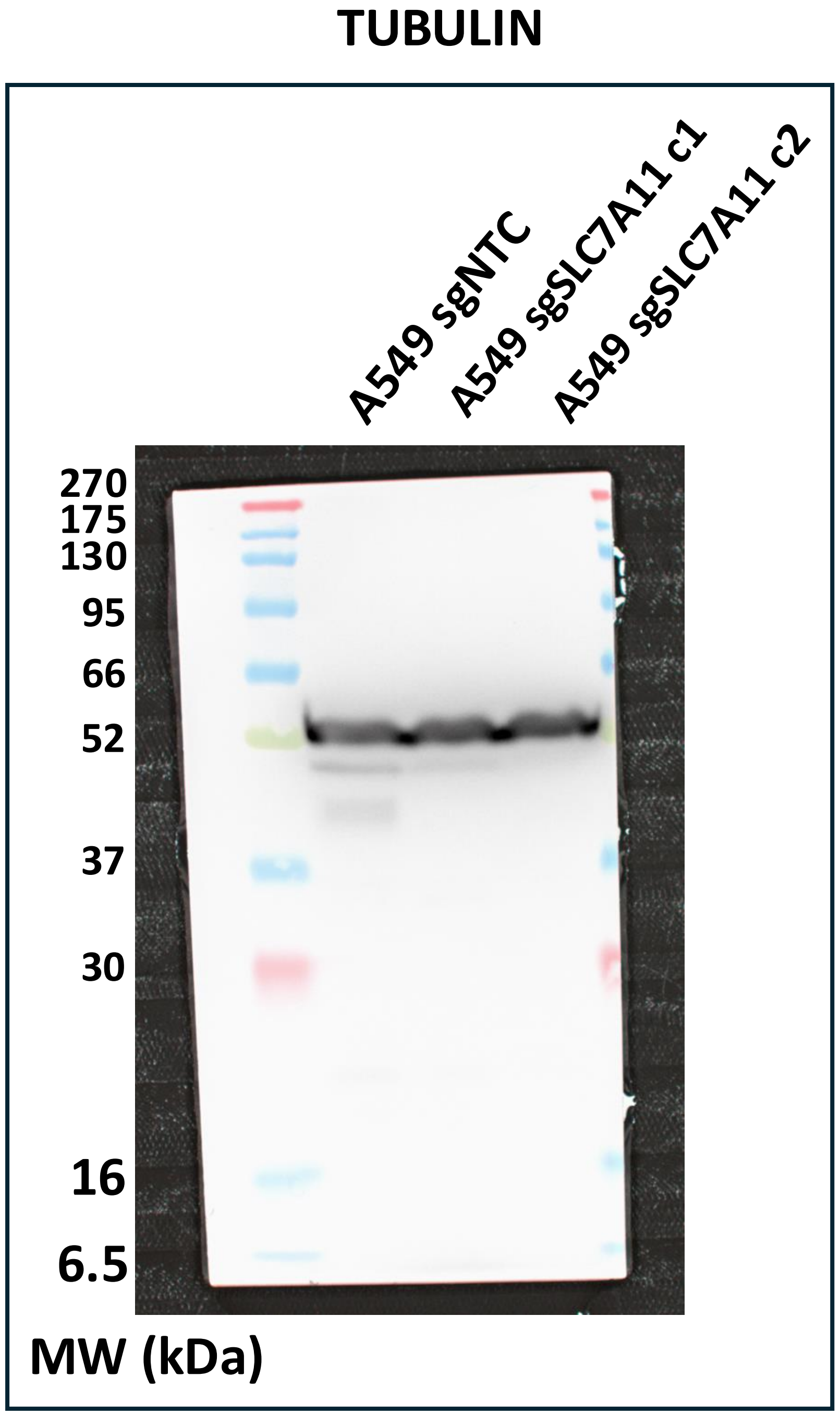

FigSup3 – C (Part 2)

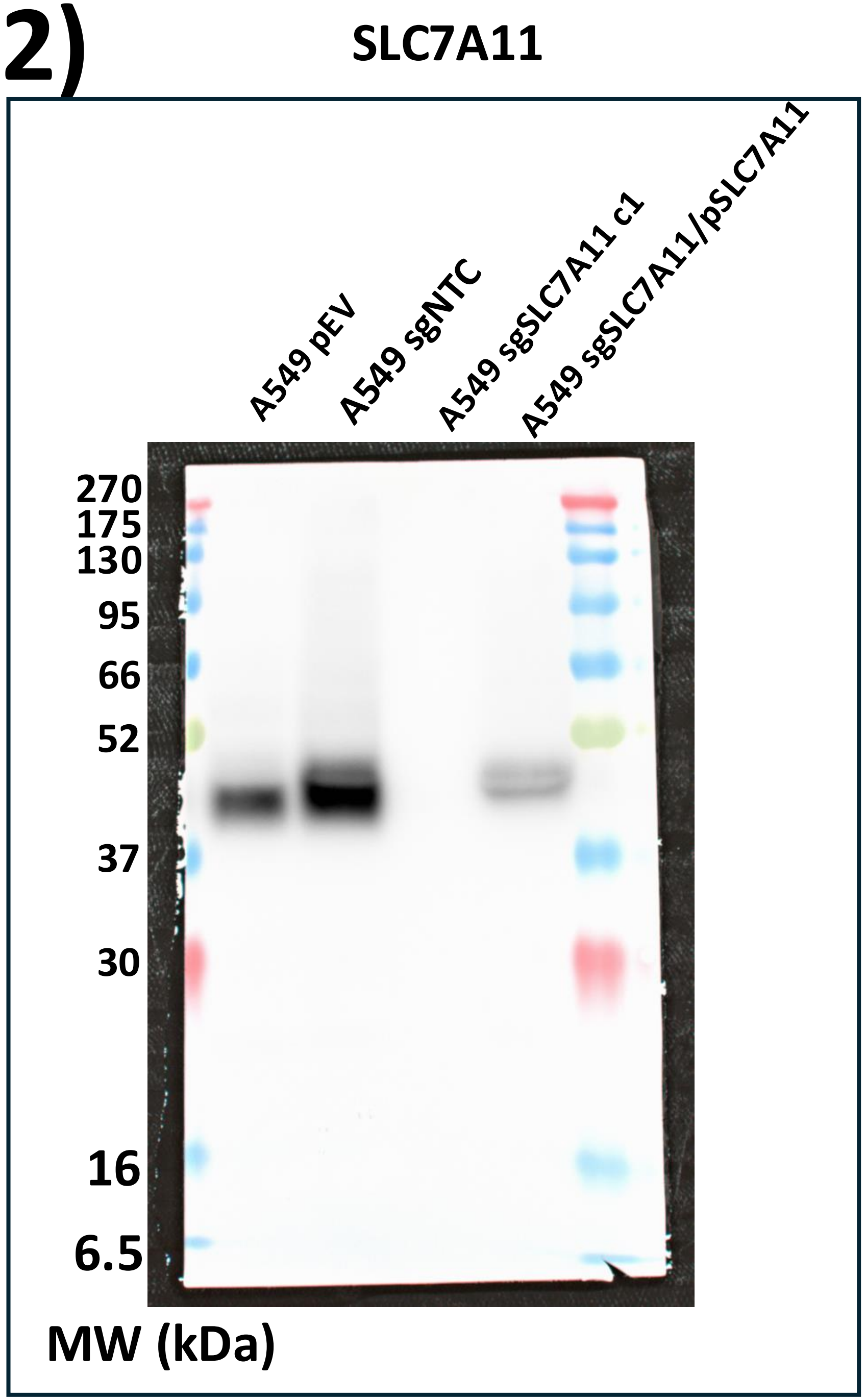

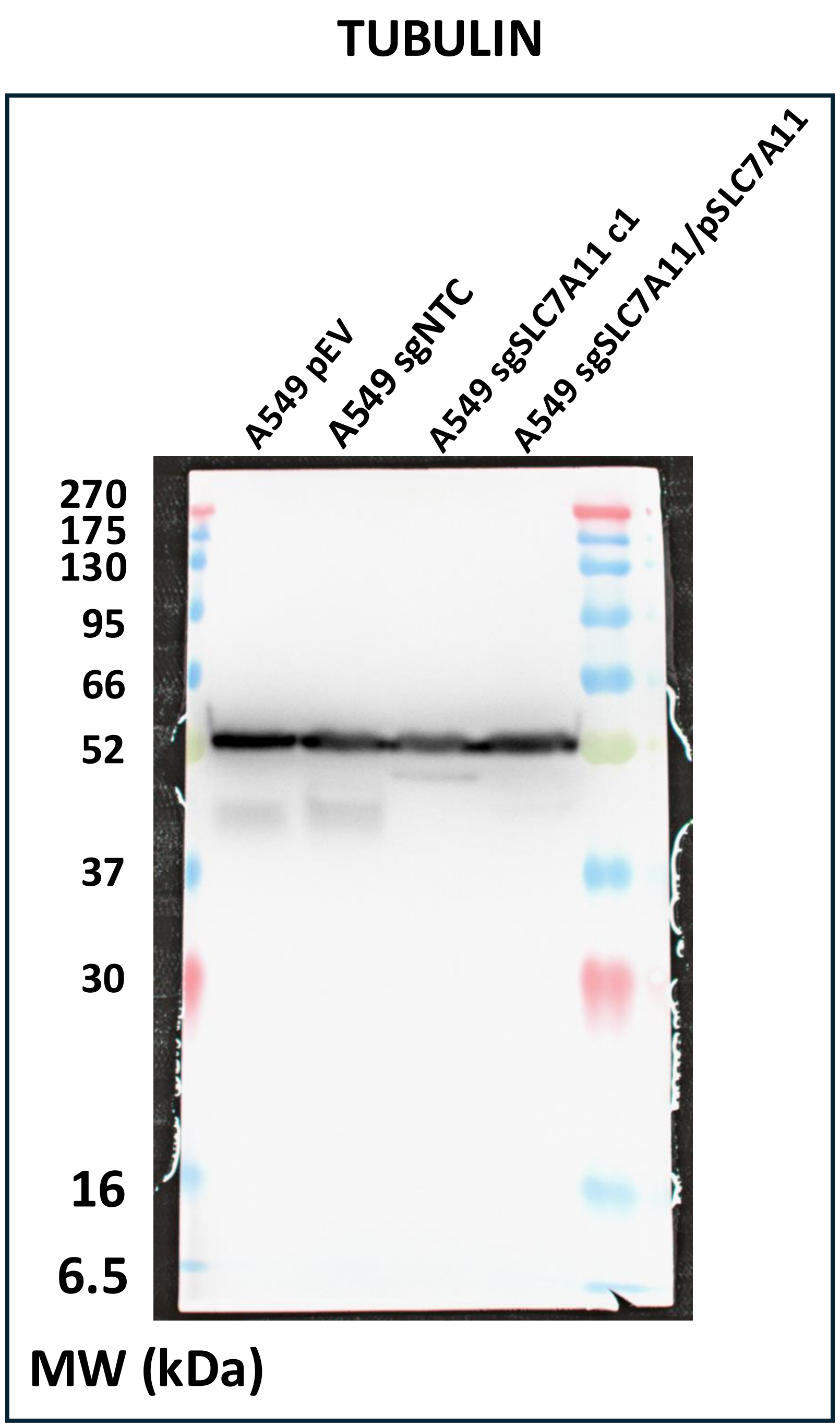

FigSup3 – D

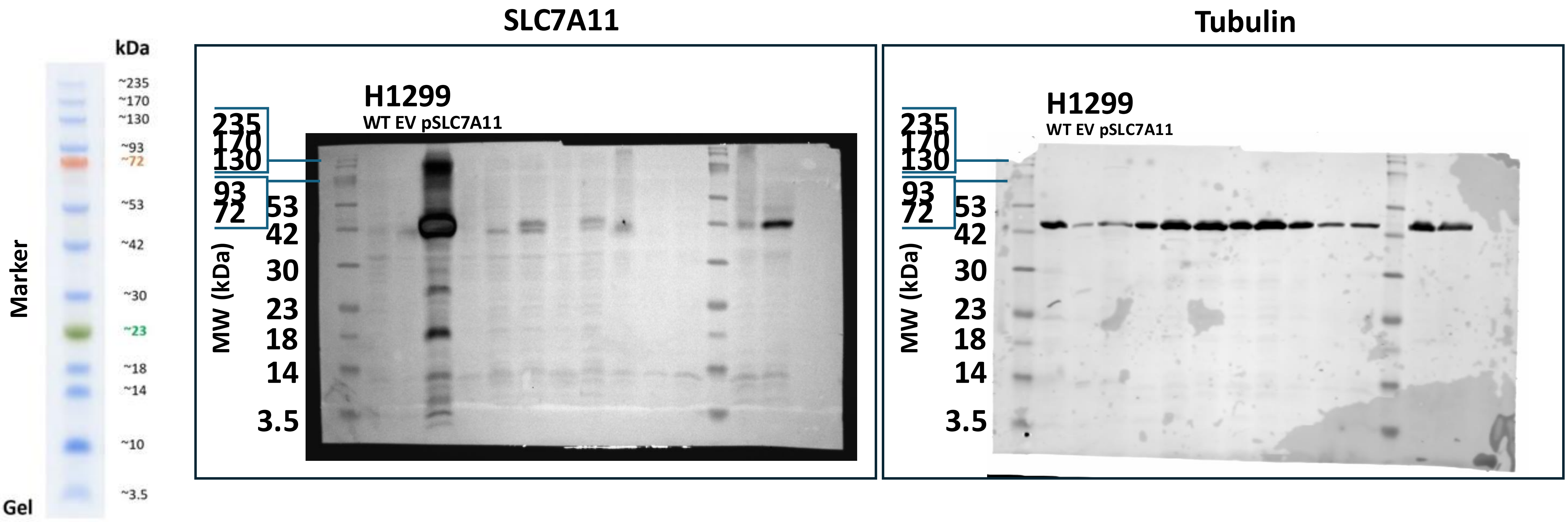

Marker
Gel
kDa
~235
~170
~130
~93
~72
~53
~42
~30
~23
~18
~14
~10
~3.5
SLC7A11
H1299
WT EV pSLC7A11
MW (kDa)
235
170
130
93
72
53
42
30
23
18
14
3.5
Tubulin
H1299
WT EV pSLC7A11
MW (kDa)
235
170
130
93
72
53
42
30
23
18
14
3.5

FigSup3 – F

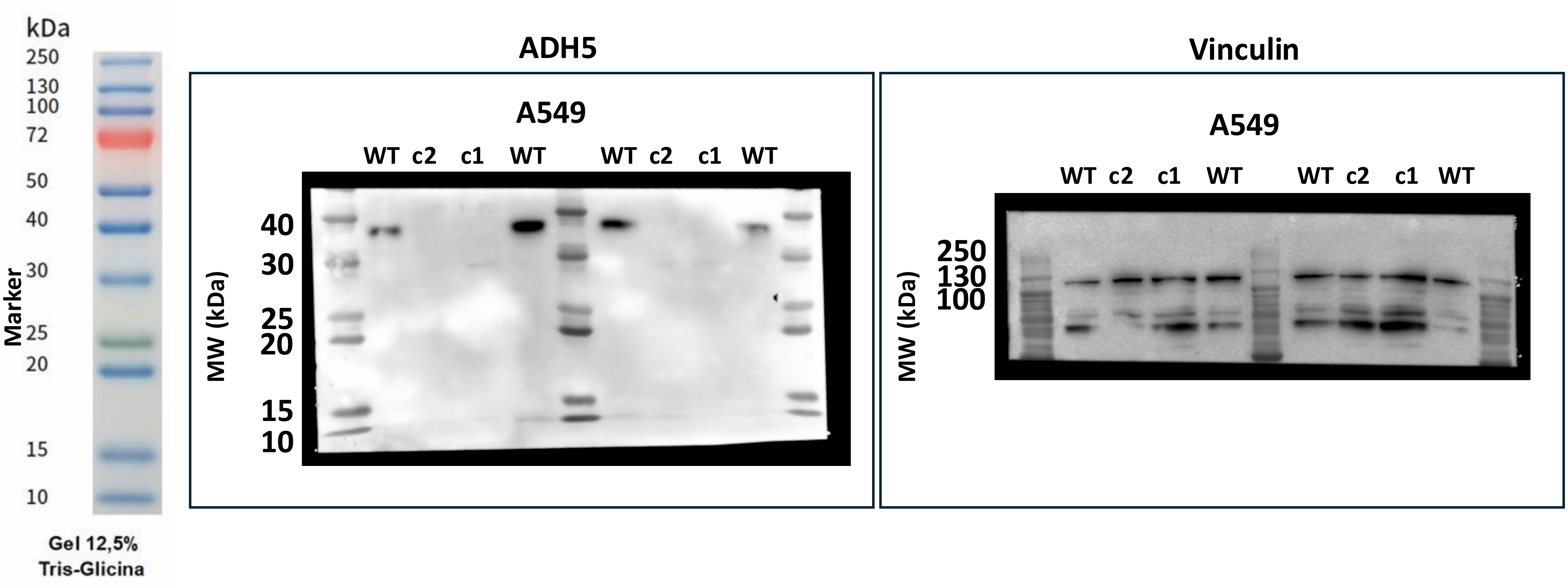
kDa
250
130
100
72
50
40
30
25
20
15
10
Marker
Gel 12,5%
Tris-Glicina
ADH5
A549
WT c2 c1 WT WT c2 c1 WT
MW (kDa)
40
30
25
20
15
10
Vinculin
A549
WT c2 c1 WT WT c2 c1 WT
MW (kDa)
250
130
100

REFERENCES

1. A. Morellato, C. Umansky, L. B. Pontel, The toxic side of one-carbon metabolism and epigenetics. *Redox Biol.* **40**, 101850 (2021).

2. G. Burgos-Barragan, N. Wit, J. Meiser, F. A. Dingler, M. Pietzke, L. Mulderrig, L. B. Pontel, I. V Rosado, T. F. Brewer, R. L. Cordell, P. S. Monks, C. J. Chang, A. Vazquez, K. J. Patel, Mammals divert endogenous genotoxic formaldehyde into one-carbon metabolism. *Nature* **548**, 549–554 (2017).

3. Y. Shi, F. Lan, C. Matson, P. Mulligan, J. R. Whetstine, P. A. Cole, R. A. Casero, Y. Shi, Histone demethylation mediated by the nuclear amine oxidase homolog LSD1. *Cell* **119**, 941–953 (2004).

4. M. Valverde-Santiago, L. B. Pontel, Emerging mechanisms underlying formaldehyde toxicity and response. *Mol. Cell* **85**, 2068–2079 (2025).

5. M. Rieckher, C. Gallrein, N. Alquezar-Artieda, N. Bourached-Silva, A. L. Vaddavalli, D. Mares, M. Backhaus, T. Blindauer, K. Greger, E. Wiesner, L. B. Pontel, B. Schumacher, Distinct DNA repair mechanisms prevent formaldehyde toxicity during development, reproduction and aging. *Nucleic Acids Res.* **52**, 8271–8285 (2024).

6. C. Umansky, A. E. Morellato, M. Rieckher, M. A. Scheidegger, M. R. Martinefski, G. A. Fernández, O. Pak, K. Kolesnikova, H. Reingruber, M. Bollini, G. P. Crossan, N. Sommer, M. E. Monge, B. Schumacher, L. B. Pontel, Endogenous formaldehyde scavenges cellular glutathione resulting in redox disruption and cytotoxicity. *Nat. Commun.* **13**, 745 (2022).

7. V. N. Pham, K. J. Bruemmer, J. D. W. Toh, E. J. Ge, L. Tenney, C. C. Ward, F. A. Dingler, C. L. Millington, C. A. Garcia-Prieto, M. C. Pulos-Holmes, N. T. Ingolia, L. B. Pontel, M. Esteller, K. J. Patel, D. K. Nomura, C. J. Chang, Formaldehyde regulates S-adenosylmethionine biosynthesis and one-carbon metabolism. *Science* **382**, eabp9201 (2023).

8. Y. Oka, M. Hamada, Y. Nakazawa, H. Muramatsu, Y. Okuno, K. Higasa, M. Shimada, H. Takeshima, K. Hanada, T. Hirano, T. Kawakita, H. Sakaguchi, T. Ichimura, S. Ozono, K. Yuge,

Y. Watanabe, Y. Kotani, M. Yamane, Y. Kasugai, M. Tanaka, T. Suganami, S. Nakada, N. Mitsutake, Y. Hara, K. Kato, S. Mizuno, N. Miyake, Y. Kawai, K. Tokunaga, M. Nagasaki, S. Kito, K. Isoyama, M. Onodera, H. Kaneko, N. Matsumoto, F. Matsuda, K. Matsuo, Y. Takahashi, T. Mashimo, S. Kojima, T. Ogi, Digenic mutations in ALDH2 and ADH5 impair formaldehyde clearance and cause a multisystem disorder, AMeD syndrome. *Sci. Adv.* **6**, eabd7197 (2020).

9. L. B. Pontel, I. V. Rosado, G. Burgos-Barragan, J. I. Garaycoechea, R. Yu, M. J. Arends, G. Chandrasekaran, V. Broecker, W. Wei, L. Liu, J. A. Swenberg, G. P. Crossan, K. J. Patel, Endogenous formaldehyde is a hematopoietic stem cell genotoxin and metabolic carcinogen. *Mol. Cell* **60**, 177–188 (2015).

10. F. A. Dingler, M. Wang, A. Mu, C. L. Millington, N. Oberbeck, S. Watcham, L. B. Pontel, A. N. Kamimae-Lanning, F. Langevin, C. Nadler, R. L. Cordell, P. S. Monks, R. Yu, N. K. Wilson, A. Hira, K. Yoshida, M. Mori, Y. Okamoto, Y. Okuno, H. Muramatsu, Y. Shiraishi, M. Kobayashi, T. Moriguchi, T. Osumi, M. Kato, S. Miyano, E. Ito, S. Kojima, H. Yabe, M. Yabe, K. Matsuo, S. Ogawa, B. Göttgens, M. R. G. Hodskinson, M. Takata, K. J. Patel, Two aldehyde clearance systems are essential to prevent lethal formaldehyde accumulation in mice and humans. *Mol. Cell* **80**, 996–1012.e9 (2020).

11. C. A. Staab, J. Ålander, R. Morgenstern, R. C. Grafström, J. O. Höög, The Janus face of alcohol dehydrogenase 3. *Chem. Biol. Interact.* **178**, 29–35 (2009).

12. H. J. Forman, H. Zhang, A. Rinna, Glutathione: Overview of its protective roles, measurement, and biosynthesis. *Mol. Aspects Med.* **30**, 1–12 (2009).

13. G. M. Denicola, F. A. Karreth, T. J. Humpton, A. Gopinathan, C. Wei, K. Frese, D. Mangal, K. H. Yu, C. J. Yeo, E. S. Calhoun, F. Scrimieri, J. M. Winter, R. H. Hruban, C. Iacobuzio-Donahue, S. E. Kern, I. A. Blair, D. A. Tuveson, Oncogene-induced Nrf2 transcription promotes ROS detoxification and tumorigenesis. *Nature* **475**, 106–109 (2011).

14. E. Kansanen, S. M. Kuosmanen, H. Leinonen, A. L. Levonenn, The Keap1-Nrf2 pathway: Mechanisms of activation and dysregulation in cancer. *Redox Biol.* **1**, 45–49 (2013).

15. S. C. Lu, Regulation of glutathione synthesis. *Mol. Aspects Med.* **30**, 42–59 (2009).

16. S. J. Yoon, J. A. Combs, A. Falzone, N. Prieto-Farigua, S. Caldwell, H. D. Ackerman, E. R. Flores, G. M. DeNicola, Comprehensive metabolic tracing reveals the origin and catabolism of cysteine in mammalian tissues and tumors. *Cancer Res.* **83**, 1426–1442 (2023).

17. Y. P. Kang, A. Mockabee-Macias, C. Jiang, A. Falzone, N. Prieto-Farigua, E. Stone, I. S. Harris, G. M. DeNicola, Non-canonical glutamate-cysteine ligase activity protects against ferroptosis. *Cell Metab.* **33**, 174–189.e7 (2021).

18. J. K. C. Kreß, C. Jessen, A. Hufnagel, W. Schmitz, T. N. Xavier da Silva, A. Ferreira dos Santos, L. Mosteo, C. R. Goding, J. P. Friedmann Angeli, S. Meierjohann, The integrated stress response effector ATF4 is an obligatory metabolic activator of NRF2. *Cell Rep.* **42**, 112724 (2023).

19. C. Sarcinelli, H. Dragic, M. Piecyk, V. Barbet, C. Duret, A. Barthelaix, C. Ferraro-Peyret, J. Fauvre, T. Renno, C. Chaveroux, S. N. Manié, ATF4-dependent NRF2 transcriptional regulation promotes antioxidant protection during endoplasmic reticulum stress. *Cancers* **12**, 569 (2020).

20. X. Chen, C. Su, S. Ren, C. Zhou, T. Jiang, Pan-cancer analysis of KEAP1 mutations as biomarkers for immunotherapy outcomes. *Ann. Transl. Med.* **8**, 141 (2020).

21. P. Koppula, G. Lei, Y. Zhang, Y. Yan, C. Mao, L. Kondiparthi, J. Shi, X. Liu, A. Horbath, M. Das, W. Li, M. V. Poyurovsky, K. Olszewski, B. Gan, A targetable CoQ-FSP1 axis drives ferroptosis- and radiation-resistance in *KEAP1* inactive lung cancers. *Nat. Commun.* **13**, 2206 (2022).

22. S. J. Dixon, K. M. Lemberg, M. R. Lamprecht, R. Skouta, E. M. Zaitsev, C. E. Gleason, D. N. Patel, A. J. Bauer, A. M. Cantley, W. S. Yang, B. Morrison, B. R. Stockwell, Ferroptosis: An iron-dependent form of nonapoptotic cell death. *Cell* **149**, 1060–1072 (2012).

23. V. I. Sayin, S. E. Leboeuf, S. X. Singh, S. M. Davidson, D. Biancur, B. S. Guzelhan, S. W. Alvarez, W. L. Wu, T. R. Karakousi, A. M. Zavitsanou, J. Ubriaco, A. Muir, D. Karagiannis, P. J. Morris, C. J. Thomas, R. Possemato, M. G. Vander Heiden, T. Papagiannakopoulos,

Activation of the NRF2 antioxidant program generates an imbalance in central carbon metabolism in cancer. *eLife* **6**, e28083 (2017).

24. S. Emmert, G. Quargnali, S. Thallmair, P. Rivera-Fuentes, A locally activatable sensor for robust quantification of organellar glutathione. *Nat. Chem.* **15**, 1415–1421 (2023).

25. M. Maiorino, M. Conrad, F. Ursini, GPx4, lipid peroxidation, and cell death: Discoveries, rediscoveries, and open issues. *Antioxid. Redox Signal.* **29**, 61–74 (2018).

26. W. S. Yang, R. Sriramaratnam, M. E. Welsch, K. Shimada, R. Skouta, V. S. Viswanathan, J. H. Cheah, P. A. Clemons, A. F. Shamji, C. B. Clish, L. M. Brown, A. W. Girotti, V. W. Cornish, S. L. Schreiber, B. R. Stockwell, Regulation of ferroptotic cancer cell death by GPX4. *Cell* **156**, 317–331 (2014).

27. G. Asantewaa, I. S. Harris, Glutathione and its precursors in cancer. *Curr. Opin. Biotechnol.* **68**, 292–299 (2021).

28. Y. P. Kang, L. Torrente, A. Falzone, C. M. Elkins, M. Liu, J. M. Asara, C. C. Dibble, G. M. Denicola, Cysteine dioxygenase 1 is a metabolic liability for non-small cell lung cancer. *eLife* **8**, e45572 (2019).

29. D. Yu, J. Liang, H. R. Widlund, P. Puigserver, Feedforward cysteine regulation maintains melanoma differentiation state and limits metastatic spread. *Cell Rep.* **43**, 114484 (2024).

30. N. P. Ward, S. J. Yoon, T. Flynn, A. M. Sherwood, M. A. Olley, J. Madej, G. M. DeNicola, Mitochondrial respiratory function is preserved under cysteine starvation via glutathione catabolism in NSCLC. *Nat. Commun.* **15**, 4244 (2024).

31. A. Singh, V. Misra, R. K. Thimmulappa, H. Lee, S. Ames, M. O. Hoque, J. G. Herman, S. B. Baylin, D. Sidransky, E. Gabrielson, M. V. Brock, S. Biswal, Dysfunctional KEAP1–NRF2 interaction in non-small-cell lung cancer. *PLoS Med.* **3**, e420 (2006).

32. L. Baird, M. Yamamoto, NRF2-dependent bioactivation of mitomycin C as a novel strategy to target KEAP1-NRF2 pathway activation in human cancer. *Mol. Cell. Biol.* **41**, e00473-20 (2021).

33. Y. Oka, Y. Nakazawa, M. Shimada, T. Ogi, Endogenous aldehyde-induced DNA–protein crosslinks are resolved by transcription-coupled repair. *Nat. Cell Biol.*, **26**, 784–796 (2024).

34. D. Pellin, M. Loperfido, C. Baricordi, S. L. Wolock, A. Montepeloso, O. K. Weinberg, A. Biffi, A. M. Klein, L. Biasco, A comprehensive single cell transcriptional landscape of human hematopoietic progenitors. *Nat. Commun.* **10**, 2395 (2019).

35. M. Pietzke, G. Burgos-Barragan, N. Wit, J. Tait-Mulder, D. Sumpton, G. M. Mackay, K. J. Patel, A. Vazquez, Amino acid dependent formaldehyde metabolism in mammals. *Commun. Chem.* **3**, 78 (2020).

36. G. Pan, C.-K. Au, Y.-H. Ham, J. Z. Yu, Z. Cai, W. Chan, Urinary thioproline and thioprolinyl glycine as specific biomarkers of formaldehyde exposure in humans. *Environ. Sci. Technol.* **58**, 16368–16375 (2024).

37. T. John, N. Saffoon, J. Walsby-Tickle, S. S. Hester, F. A. Dingler, C. L. Millington, J. S. O. McCullagh, K. J. Patel, R. J. Hopkinson, C. J. Schofield, Aldehyde-mediated inhibition of asparagine biosynthesis has implications for diabetes and alcoholism. *Chem. Sci.* **15**, 2509–2517 (2024).

38. R. J. Hopkinson, P. S. Barlow, C. J. Schofield, T. D. W. Claridge, Studies on the reaction of glutathione and formaldehyde using NMR. *Org. Biomol. Chem.* **8**, 4915–4920 (2010).

39. J. J. A. G. Kamps, R. J. Hopkinson, C. J. Schofield, T. D. W. Claridge, How formaldehyde reacts with amino acids. *Commun. Chem.* **2**, 126 (2019).

40. J. A. Brain, A.-L. B. G. Vigil, K. Davidsen, A. Itokawa, A. C. Jurasin, H. J. Kerbyson, M. Kobiesa, M. L. Hart, S. J. Yoon, P. Bellotti, J. P. Maianti, G. M. DeNicola, L. B. Sullivan, Excess cysteine drives conjugate formation and impairs proliferation of NRF2-activated cancer cells. *Nat. Metab.* **8**, 840–854 (2026).

41. K. Pakos-Zebrucka, I. Koryga, K. Mnich, M. Ljujic, A. Samali, A. M. Gorman, The integrated stress response. *EMBO Rep.* **17**, 1374–1395 (2016).

42. S. Zhao, J. Cordes, K. M. Caban, M. J. Götz, T. Mackens-Kiani, A. J. Veltri, N. K. Sinha, P. Weickert, S. Kaya, G. Hewitt, D. D. Nedialkova, T. Fröhlich, R. Beckmann, A. R. Buskirk, R. Green, J. Stingele, RNF14-dependent atypical ubiquitylation promotes translation-coupled resolution of RNA-protein crosslinks. *Mol. Cell* **83**, 4290–4303.e9 (2023).

43. A. Suryo Rahmanto, C. J. Blum, C. Scalera, J. B. Heidelberger, M. Mesitov, D. Horn-Ghetko, J. F. Gräf, I. Mikicic, R. Hobrecht, A. Orekhova, M. Ostermaier, S. Ebersberger, M. M. Möckel, N. Krapoth, N. Da Silva Fernandes, A. Mizi, Y. Zhu, J.-X. Chen, C. Choudhary, A. Papantonis, H. D. Ulrich, B. A. Schulman, J. König, P. Beli, K6-linked ubiquitylation marks formaldehyde-induced RNA-protein crosslinks for resolution. *Mol. Cell* **83**, 4272–4289.e10 (2023).

44. M. Olivieri, T. Cho, A. Álvarez-Quilón, K. Li, M. J. Schellenberg, M. Zimmermann, N. Hustedt, S. E. Rossi, S. Adam, H. Melo, A. M. Heijink, G. Sastre-Moreno, N. Moatti, R. K. Szilard, A. McEwan, A. K. Ling, A. Serrano-Benitez, T. Ubhi, S. Feng, J. Pawling, I. Delgado-Sainz, M. W. Ferguson, J. W. Dennis, G. W. Brown, F. Cortés-Ledesma, R. S. Williams, A. Martin, D. Xu, D. Durocher, A genetic map of the response to DNA damage in human cells. *Cell* **182**, 481–496.e21 (2020).

45. H. Esterbauer, K. H. Cheeseman, M. U. Dianzani, G. Poli, T. F. Slater, Separation and characterization of the aldehydic products of lipid peroxidation stimulated by ADP-Fe^{2+} in rat liver microsomes. *Biochem. J.* **208**, 129–140 (1982).

46. J. Lippmann, K. Petri, S. Fulda, J. Liese, Redox modulation and induction of ferroptosis as a new therapeutic strategy in hepatocellular carcinoma. *Transl. Oncol.* **13**, 100785 (2020).

47. H. Ogiwara, K. Takahashi, M. Sasaki, T. Kuroda, H. Yoshida, R. Watanabe, A. Maruyama, H. Makinoshima, F. Chiwaki, H. Sasaki, T. Kato, A. Okamoto, T. Kohno, Targeting the vulnerability of glutathione metabolism in ARID1A-deficient cancers. *Cancer Cell* **35**, 177–190.e8 (2019).

48. L. B. Pontel, A. Bueno-Costa, A. E. Morellato, J. Carvalho Santos, G. Roué, M. Esteller, Acute lymphoblastic leukemia necessitates GSH-dependent ferroptosis defenses to overcome FSP1-epigenetic silencing. *Redox Biol.* **55**, 102408 (2022).

49. J. D. Hayes, A. T. Dinkova-Kostova, K. D. Tew, Oxidative stress in cancer. *Cancer Cell* **38**, 167–197 (2020).

50. C.-S. Shin, P. Mishra, J. D. Watrous, V. Carelli, M. D'Aurelio, M. Jain, D. C. Chan, The glutamate/cystine xCT antiporter antagonizes glutamine metabolism and reduces nutrient flexibility. *Nat. Commun.* **8**, 15074 (2017).

51. P. Koppula, Y. Zhang, L. Zhuang, B. Gan, Amino acid transporter SLC7A11/xCT at the crossroads of regulating redox homeostasis and nutrient dependency of cancer. *Cancer Commun.* **38**, 12 (2018).

52. Y. Yan, H. Teng, Q. Hang, L. Kondiparthi, G. Lei, A. Horbath, X. Liu, C. Mao, S. Wu, L. Zhuang, M. James You, M. V. Poyurovsky, L. Ma, K. Olszewski, B. Gan, SLC7A11 expression level dictates differential responses to oxidative stress in cancer cells. *Nat. Commun.* **14**, 3673 (2023).

53. T. Weiss-Sadan, M. Ge, M. Hayashi, M. Gohar, C.-H. Yao, A. de Groot, S. Harry, A. Carlin, H. Fischer, L. Shi, T.-Y. Wei, C. H. Adelmann, K. Wolf, T. Vornbäumen, B. R. Dürr, M. Takahashi, M. Richter, J. Zhang, T.-Y. Yang, V. Vijay, D. E. Fisher, A. N. Hata, M. C. Haigis, R. Mostoslavsky, N. Bardeesy, T. Papagiannakopoulos, L. Bar-Peled, NRF2 activation induces NADH-reductive stress, providing a metabolic vulnerability in lung cancer. *Cell Metab.* **35**, 487–503.e7 (2023).

54. C. Xia, X. Xing, W. Zhang, Y. Wang, X. Jin, M. Tian, X. Ba, F. Hao, Cysteine and homocysteine can be exploited by GPX4 in ferroptosis inhibition independent of GSH synthesis. *Redox Biol.* **69**, 102999 (2024).

55. P. Nowak-Sliwinska, T. Segura, M. L. Iruela-Arispe, The chicken chorioallantoic membrane model in biology, medicine and bioengineering. *Angiogenesis* **17**, 779–804 (2014).

56. I. S. Harris, A. E. Treloar, S. Inoue, M. Sasaki, C. Gorrini, K. C. Lee, K. Y. Yung, D. Brenner, C. B. Knobbe-Thomsen, M. A. Cox, A. Elia, T. Berger, D. W. Cescon, A. Adeoye, A. Brüstle, S. D. Molyneux, J. M. Mason, W. Y. Li, K. Yamamoto, A. Wakeham, H. K. Berman, R. Khokha,

S. J. Done, T. J. Kavanagh, C.-W. Lam, T. W. Mak, Glutathione and thioredoxin antioxidant pathways synergize to drive cancer initiation and progression. *Cancer Cell* **27**, 211–222 (2015).

57. E. M. Tacconi, X. Lai, C. Folio, M. Porru, G. Zonderland, S. Badie, J. Michl, I. Sechi, M. Rogier, V. Matía García, A. S. Batra, O. M. Rueda, P. Bouwman, J. Jonkers, A. Ryan, B. Reina-San-Martin, J. Hui, N. Tang, A. Bruna, A. Biroccio, M. Tarsounas, BRCA 1 and BRCA 2 tumor suppressors protect against endogenous acetaldehyde toxicity. *EMBO Mol. Med.* **9**, 1398–1414 (2017).

58. B. K. Tusi, S. L. Wolock, C. Weinreb, Y. Hwang, D. Hidalgo, R. Zilionis, A. Waisman, J. R. Huh, A. M. Klein, M. Socolovsky, Population snapshots predict early haematopoietic and erythroid hierarchies. *Nature* **555**, 54–60 (2018).

59. J. Chen, H. Yang, A. S. M. Teo, L. B. Amer, F. G. Sherbaf, C. Q. Tan, J. J. S. Alvarez, B. Lu, J. Q. Lim, A. Takano, R. Nahar, Y. Y. Lee, C. Z. J. Phua, K. P. Chua, L. Suteja, P. J. Chen, M. M. Chang, T. P. T. Koh, B.-H. Ong, D. Anantham, A. A. L. Hsu, A. Gogna, C. W. Too, Z. W. Aung, Y. F. Lee, L. Wang, T. K. H. Lim, A. Wilm, P. S. Choi, P. Y. Ng, C. K. Toh, W.-T. Lim, S. Ma, B. Lim, J. Liu, W. L. Tam, A. J. Skanderup, J. P. S. Yeong, E.-H. Tan, C. L. Creasy, D. S. W. Tan, A. M. Hillmer, W. Zhai, Genomic landscape of lung adenocarcinoma in East Asians. *Nat. Genet.* **52**, 177–186 (2020).

60. H. B. Lengel, B. Mastrogiacomo, J. G. Connolly, K. S. Tan, Y. Liu, C. N. Fick, E. G. Dunne, D. He, M. B. Lankadasari, B. A. Satravada, Y. Sun, R. Kundra, C. Fong, S. Smith, G. J. Riely, C. M. Rudin, D. R. Gomez, D. B. Solit, M. F. Berger, B. T. Li, M. W. Mayo, I. Matei, D. C. Lyden, P. S. Adusumilli, N. Schultz, F. Sanchez-Vega, D. R. Jones, Genomic mapping of metastatic organotropism in lung adenocarcinoma. *Cancer Cell* **41**, 970–985.e3 (2023).
